# Highly chemo-, enantio-, and diastereoselective [4 + 2] cycloaddition of 5*H*-thiazol-4-ones with *N*-itaconimides

**DOI:** 10.3762/bjoc.12.222

**Published:** 2016-11-01

**Authors:** Shuai Qiu, Choon-Hong Tan, Zhiyong Jiang

**Affiliations:** 1 Key Laboratory of Natural Medicine and Immuno-Engineering of Henan Province, Henan University, Kaifeng, 475004, Henan, P. R. China; 2 Division of Chemistry and Biological Chemistry, Nanyang Technological University, 21 Nanyang Link, 637371, Singapore

**Keywords:** [4 + 2] annulation, asymmetric organocatalysis, dipeptide-based Brønsted bases, 5*H*-thiazol-4-ones, *N*-itaconimides

## Abstract

A dipeptide-based urea-amide tertiary amine (DP-UAA) was shown to be an effective Brønsted base catalyst for the first asymmetric annulation reaction between 5*H*-thiazol-4-ones and *N*-itaconimides. High levels of enantioselectivity (up to 99% ee) and diastereoselectivity (>19:1 dr) were obtained for a series of spirocyclic 1,4-sulfur-bridged piperidinone-based succinimides.

## Introduction

Sulfur-containing tetrasubstituted carbon stereocenters are widely present in a number of natural and non-natural products with significant biological activities [1]. In the past few decades, diverse competent asymmetric strategies have been established to access these chiral entities [1–4]. Among them, catalytic asymmetric functionalization of *S*-containing prochiral carbon centers can be considered to be one of the most efficient and expedient approach [1–4]. The development of novel *S*-containing substrates has therefore attracted the attention of chemists [1–4]. For example in 2013, Palomo and co-workers introduced 5*H*-thiazol-4-ones as a new class of sulfur-containing pro-nucleophiles in a highly enantio- and diastereoselective conjugate addition to nitroalkenes, providing α,α-disubstituted α-mercapto carboxylic acids [5]. Since then, several asymmetric variants using 5*H*-thiazol-4-ones as nucleophiles have been disclosed; such as amination [6], allylation [7], conjugate addition to enones [8], and γ-addition with allenoates [9]. All these examples focused on nucleophilic addition reactions of the C5 atom of 5*H*-thiazol-4-ones.

Recently, we described an organocatalytic asymmetric [4 + 2] cyclization of 5*H*-thiazol-4-ones with a series of activated alkenes, including nitroalkenes, 4-oxo-4-arylbutenones, 4-oxo-4-arylbutenoates and methyleneindolinones [10]. This work elucidated the feasibility of the electrophilic addition to the C2 position of 5*H*-thiazol-4-ones. More importantly, it provided a direct and convenient approach to synthesize three kinds of biologically important chiral 1,4-sulfur-bridged piperidinones and their related derivatives [10]. In order to develop novel chiral *S*-containing polycyclic scaffolds, the development of [4 + 2] annulations of 5*H*-thiazol-4-ones with unusual activated alkenes still remains highly desirable.

Succinimides are present in many biologically significant molecules and are investigated as potential pharmacophores in the research of drug discovery [11–12]. Our group has recently devised *N*-itaconimides for the assembly of succinimide frameworks [13–18]. As an extension of these works, herein, we report an asymmetric [4 + 2] annulation reaction of 5*H*-thiazol-4-ones with *N*-itaconimides. The method features excellent chemo-, enantio, and diastereoselectivities, thus leading to a series of chiral spirocyclic 1,4-sulfur-bridged piperidinone-based succinimides with excellent results.

## Results and Discussion

Our studies were initiated by examining a model reaction between 5*H*-thiazol-4-one **1a** and *N*-phenyl itaconimide **2a** (Table 1). The reaction was first evaluated in toluene at 25 °C and using *L*-*tert*-leucine-based thiourea−tertiary amine **I** as the catalyst (Table 1, entry 1), with excellent catalytic efficacy as demonstrated in a series of asymmetric reactions [18]. It was found that the reaction was completed after 48 hours, affording the desired [4 + 2] annulation adduct **3a** in 55% yield with 64% ee. A significant amount of conjugate addition adduct led to the unsatisfactory chemoselectivity and thus the moderate yield. When the H-bond donor was changed from thiourea to urea (catalyst **II**), it did not provide better results (Table 1, entry 2) [17,19]. In the [4 + 2] annulation of 5*H*-thiazol-4-ones with nitroalkenes, dipeptide-based thiourea−amide−tertiary amine **III** (DP-TAA) was devised and demonstrated as a competent catalyst to furnish excellent chemo- and stereoselectivity [10]. Therefore, we examined catalyst **III** for this reaction (Table 1, entry 3); annulation adduct **3a** was obtained in 60% yield with 70% ee. The increased in enantioselectivity indicates the potential of dipeptide-based tertiary amine for this type of reaction. By modifying the thiourea moiety of **III** to urea lead us to catalyst DP-UAA **IV**, which could further increase the enantioselectivity (Table 1, entry 4). Subsequently, we screened the solvent effect with **IV** as the catalyst (Table 1, entries 5–7), and the results revealed that chloroform was the best reaction medium regarding the enantioselectivity (Table 1, entry 7). By changing the reaction temperature (Table 1, entries 8 and 9), we found that the yield of **3a** was decreased to 60% but the ee value was increased to 92% at −10 °C (Table 1, entry 9). To improve the chemoselectivity, we synthesized a series of DP-UAAs through tuning of the substituent groups of the urea. We were pleased to find that the reaction rate could be tremendously increased by utilizing DP-UAA **V** as the catalyst, and **3a** was obtainable with high enantioselectivity, high chemoselectivity and excellent yield of 98% (Table 1, entry 10).

**Table 1:** Optimization of reaction conditions^a^.

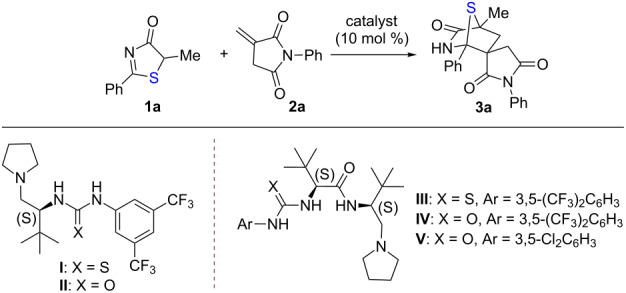						

Entry	Catalyst	Solvent	*T* (°C)	*t* (h)	Yield (%)^b^	ee (%)^c^

1	**I**	toluene	25	48	55	64
2	**II**	toluene	25	48	50	62
3	**III**	toluene	25	48	60	70
4	**IV**	toluene	25	48	62	74
5	**IV**	CH_2_Cl_2_	25	48	68	77
6	**IV**	Et_2_O	25	48	50	76
7	**IV**	CHCl_3_	25	48	72	86
8	**IV**	CHCl_3_	0	48	70	89
9	**IV**	CHCl_3_	−10	60	60	92
10	**V**	CHCl_3_	−10	18	98	93

^a^The reaction was performed in a 0.05 mmol scale; ^b^yield was isolated by flash column; ^c^ee was determined by HPLC.

**Scheme 1:**
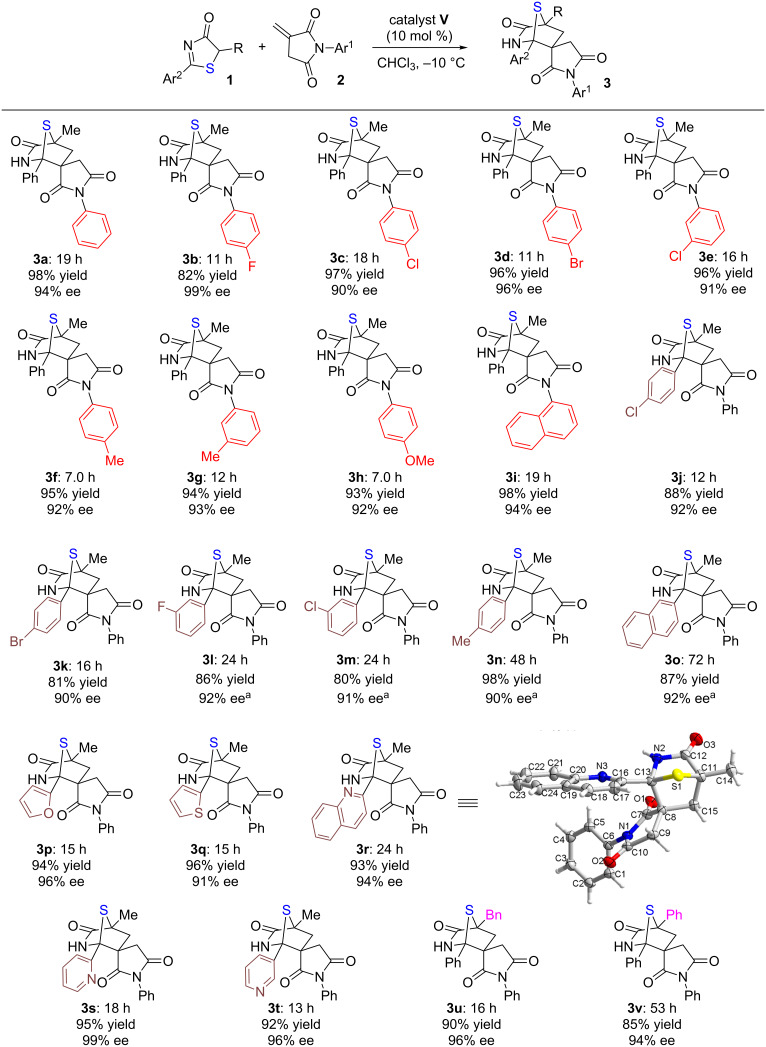
Substrate scope of the [4 + 2] annulation. Reaction conditions: **1** (0.1 mmol), **2** (0.15 mmol), **V** (0.01 mmol), CHCl_3_ (1.0 mL) at −10 °C. All drs are >20:1; ees were determined via chiral HPLC analysis. ^a^20 mol % of **V** was used, *T* = 0 °C.

With the optimal reaction conditions in hand, we examined the substrate scope of the enantioselective [4 + 2] cycloaddition between 5*H*-thiazol-4-ones **1** and *N*-itaconimides **2**, catalyzed by DP-UAA **V** (Scheme 1). Firstly, with **1a** as the model 5*H*-thiazol-4-one substrate, a series of *N*-itaconimides containing various *N*-aryl groups were transformed to the corresponding [4 + 2] annulation adducts **3a–i** in 82−98% yield with 90−96% ee. We then investigated substrates with diverse aromatic (**3j–o**) and heteroaromatic (**3p–t**) groups on the 2-position of 5*H*-thiazol-4-ones, and found the reactions completed within 12−72 hours, affording the corresponding annulation adducts **3j–t** in 80−98% yield with 90−99% ee. Altering the R group on 5*H*-thiazol-4-ones for a benzyl (**3u**) or phenyl (**3v**) substituent also presented **3u** and **3v** in high yields and excellent enantioselectivities. The absolute configurations of annulation adducts **3** were assigned based on X-ray crystallographic analysis of a single crystal of **3r** [20].

Through an analysis of the absolute configuration of adduct **3**, it is proposed that a plausible reaction mechanism should be similar to the DP-TAA-catalyzed [4 + 2] annulation between 5*H*-thiazol-4-ones and nitroalkenes [10]. In this stepwise process, the use of 3,5-dichlorophenyl as the substituent group of the urea in catalyst **V** would remarkably increase the free energy difference between *R*- and *S*-selection in the first Michael addition step, and also decreases the free energy of the second formal Mannich reaction, thus improving the reaction rate and chemoselectivity of the [4 + 2] cycloaddition.

**Scheme 2:**
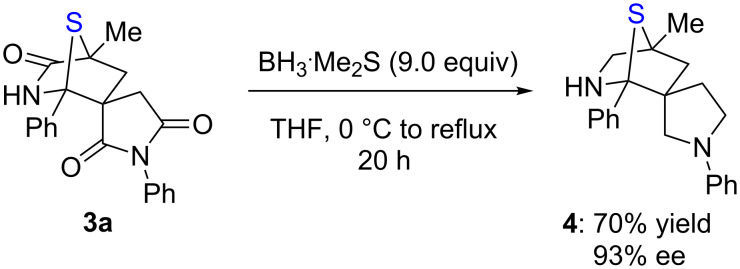
Transformation of adduct.

To extend the usefulness of this reaction, we demonstrate that the adduct can be efficiently reduced with borane. As shown in Scheme 2, **3a** was readily reduced to afford a spirocyclic sulfur-bridged *N*-heterocycle **4** in 70% yield and without compromising its ee value.

## Conclusion

In conclusion, we have developed the first asymmetric reaction of 5*H*-thiazol-4-ones with *N*-itaconimides. By employing a DP-UAA catalyst, the reaction undergoes a [4 + 2] annulation process with excellent chemoselectivity and with a broad substrate scope, affording a series of valuable chiral spirocyclic 1,4-sulfur-bridged piperidinone-based succinimides in high yields (up to 98%) and excellent enantio- and diastereoselectivities (up to 99% ee and >19:1 dr). Further investigations involving new [4 + 2] annulation of 5*H*-thiazol-4-ones using DP-TAAs and DP UAAs are currently ongoing and will be reported in due course.

## Experimental

**Representative procedure for the synthesis of 3a**: *N*-Phenyl itaconimide **2a** (0.15 mmol, 1.5 equiv) and catalyst **V** (0.01 mmol, 0.1 equiv) were dissolved in chloroform (1.0 mL) and stirred at −10 °C for 10 min. This is followed by the addition of 5*H*-thiazol-4-one **1a** (0.1 mmol, 1.0 equiv). The reaction mixture was stirred at −10 °C and monitored by TLC. Upon complete consumption of **1a**, the reaction mixture was directly loaded onto a short silica gel column, followed by gradient elution with DCM/MeOH mixture (500:1−200:1 ratio). Removing the solvent in vacuo, afforded product **3a**.

## Supporting Information

File 1 Experimental information and spectroscopic data.
