# Supplementary material for: Highly chemo-, enantio-, and diastereoselective [4 + 2] cycloaddition of 5H-thiazol-4-ones with N-itaconimides
Source: Beilstein J Org Chem. 2016 Nov 1;12:2293–7. doi: 10.3762/bjoc.12.222 (PMC5238619; doi:10.3762/bjoc.12.222)
Supplement: File 1 — Experimental information and spectroscopic data. [file Beilstein_J_Org_Chem-12-2293-s001.pdf]

Supporting Information

for

Highly chemo-, enantio-, and diastereoselective [4 + 2] cycloaddition of 5*H*-thiazol-4-ones with *N*-itaconimides

Shuai Qiu[1], Choon-Hong Tan*[,2] and Zhiyong Jiang[*,1]

Address: [1]Key Laboratory of Natural Medicine and Immuno-Engineering of Henan Province, Henan University, Kaifeng, Henan, P. R. China, 475004 and [2]Division of Chemistry and Biological Chemistry, Nanyang Technological University, 21 Nanyang Link, Singapore, 637371

Email: Zhiyong Jiang - chmjzy@henu.edu.cn; Choon-Hong Tan - choonhong@ntu.edu.sg

*Corresponding author

Experimental information and spectroscopic data

Table of contents

1. General information S3–4

2. Characterization of adducts S5–21

3. Determination of the absolute configuration by X-ray crystallography S22

4. Copies of NMR spectra S23–45

1. General information

General Procedures and Methods

Experiments involving moisture and/or air sensitive components were performed under a positive pressure of nitrogen in oven-dried glassware equipped with a rubber septum inlet. Dried solvents and liquid reagents were transferred by oven-dried syringes or hypodermic syringe cooled to ambient temperature in a desiccator. Reactions mixtures were stirred in 10 mL sample vial with Teflon-coated magnetic stirring bars unless otherwise stated. Moisture in non-volatile reagents/compounds was removed in high *vacuo* by means of an oil pump and subsequent purging with nitrogen. Solvents were removed in vacuo under ~30 mmHg and heated with a water bath at 30–35 ^{o}C using Changcheng rotary evaporator with Changcheng aspirator. The condenser was cooled with running water at 0 ^{o}C.

All experiments were monitored by analytical thin layer chromatography (TLC). TLC was performed on pre-coated plates, 60 F_{254}. After elution, plate was visualized under UV illumination at 254 nm for UV active material. Further visualization was achieved by staining $KMnO_4$, ceric molybdate, or anisaldehyde solution. For those using the aqueous stains, the TLC plates were heated on a hot plate.

Columns for flash chromatography (FC) contained silica gel 200-300 mesh. Columns were packed as slurry of silica gel in petroleum ether and equilibrated solution using the appropriate solvent system. The elution was assisted by applying pressure of about 2 atm with an air pump.

Instrumentations

Proton nuclear magnetic resonance (^{1}H NMR) and carbon NMR (^{13}C NMR) were recorded in $CDCl_3$ otherwise stated. Chemical shifts are reported in parts per million (ppm), using the residual solvent signal as an internal standard: $CDCl_3$ (^{1}H NMR: δ 7.26, singlet; ^{13}C NMR: δ 77.0, triplet). Multiplicities were given as: *s* (singlet), *d* (doublet), *t* (triplet), *q* (quartet), *quintet*, *m* (multiplets), *dd* (doublet of doublets), *dt* (doublet of triplets), and *br* (broad). Coupling constants (*J*) were recorded in Hertz (Hz). The number of proton atoms (*n*) for a given resonance was indicated by *n*H. The number of carbon atoms (*n*) for a given resonance was indicated by *n*C. HRMS was reported in units of mass of charge ratio (m/z). Mass samples were dissolved in CH_3CN (HPLC Grade) unless otherwise stated. Optical rotations

were recorded on a polarimeter with a sodium lamp of wavelength 589 nm and reported as follows; $[\alpha]_{\lambda}^{T°C}$ (c = g/100 mL, solvent). Melting points were determined on a melting point apparatus.

Enantiomeric excesses were determined by chiral High Performance Liquid Chromatography (HPLC) analysis. UV detection was monitored at 254 nm, 230 nm and 210 nm at the same time. HPLC samples were dissolved in HPLC grade isopropanol (IPA) unless otherwise stated.

Materials

All commercial reagents were purchased with the highest purity grade. They were used without further purification unless specified. All solvents used, mainly petroleum ether (PE) and ethyl acetate (EtOAc) were distilled. Anhydrous DCM and $CHCl_3$ were freshly distilled from CaH_2 and stored under N_2 atmosphere. Et_2O and toluene were freshly distilled from sodium/benzophenone before use. Anhydrous methanol and etanol were distilled from Mg. All compounds synthesized were stored in a −20 °C freezer and light-sensitive compounds were protected with aluminium foil.

2. Characterization of adducts

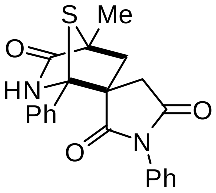

3a, white solid, Mp 101.7–102.5 °C; 37.1 mg (0.1 mmol), 98% yield; 94% *ee*; $[\alpha]_D^{26}$ 42.2 (*c* 1.0, $CHCl_3$); ^1H NMR (300 MHz, $CDCl_3$) δ 7.34–7.50 (m, 6H), 7.22 (d, *J* = 6.7 Hz, 2H), 7.06 (d, 2H), 6.87 (s, 1H), 3.05 (d, *J* = 18.9 Hz, 1H), 2.98 (d, *J* = 13.0 Hz, 1H), 2.89 (d, *J* = 18.9 Hz, 1H), 2.45 (d, *J* = 13.0 Hz, 1H); 13C NMR (75 MHz, $CDCl_3$) δ 177.4, 176.45, 172.7, 131.4, 130.8, 130.4, 129.3, 129.1, 128.7, 126.3, 126.0, 84.2, 61.4, 60.5, 51.5, 44.1, 14.9; HRMS (ESI) m/z 379.1124 ($M+H^+$), calc. for $C_{21}H_{19}N_2O_3S$ 379.1118.

The *ee* was determined by HPLC analysis. CHIRALPAK IB-3 (4.6 mm i.d. x 250 mm); Hexane/2-propanol = 70/30; flow rate 1.0 mL/min; 25 °C; 254 nm; retention time: 13.7 min (minor) and 27.4 min (major).

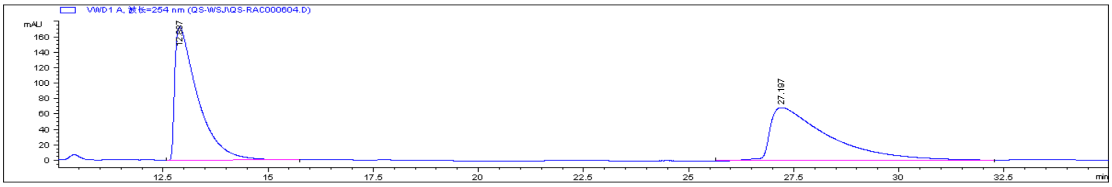

Entry	Retention Time	Area	Height	%Area
1	12.887	6530.6	172.8	48.994
2	27.197	6798.9	69	51.006

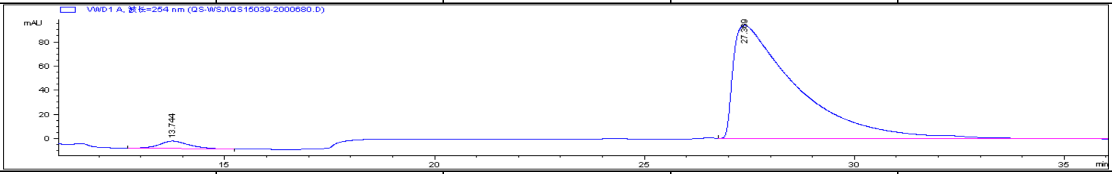

Entry	Retention Time	Area	Height	%Area
1	13.744	319.4	6.3	3.094
2	27.369	10005.5	94.2	96.906

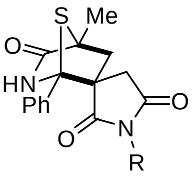

R = 4-FPh

3b, white solid, Mp 108.9–109.9 °C; 32.5 mg (0.1 mmol), 82% yield; 99% ee; $[\alpha]_D^{26}$ 17.66 (*c* 1.0, $CHCl_3$); ^1H NMR (300 MHz, $CDCl_3$) δ 7.51–7.40 (m, 3H), 7.20 (dd, *J* = 8.0, 1.3 Hz, 2H), 7.13–7.02 (m, 4H), 6.93 (s, 1H), 3.06 (d, *J* = 18.9 Hz, 1H), 2.97 (d, *J* = 13.0 Hz, 1H), 2.88 (d, *J* = 19.0 Hz, 1H), 2.44 (d, *J* = 13.0 Hz, 1H), 1.76 (s, 3H); ^{13}C NMR (75 MHz, $CDCl_3$) δ 177.4, 176.4, 172.6, 163.9, 160.5, 130.8, 130.4, 129.3, 127.9. 127.8, 127.3 (two peaks), 126.2, 116.3, 116.0, 84.2, 61.5, 60.5, 51.4, 44.1, 14.9; HRMS (ESI) m/z 397.1028 ($M+H^+$), calc. for $C_{21}H_{18}N_2O_3SF$ 397.1022.

The *ee* was determined by HPLC analysis. CHIRALPAK IB-3 (4.6 mm i.d. x 250 mm);

Hexane/2-propanol = 70/30; flow rate 1.0 mL/min; 25 °C; 254 nm; retention time: 12.7 min (minor) and 18.8 min (major).

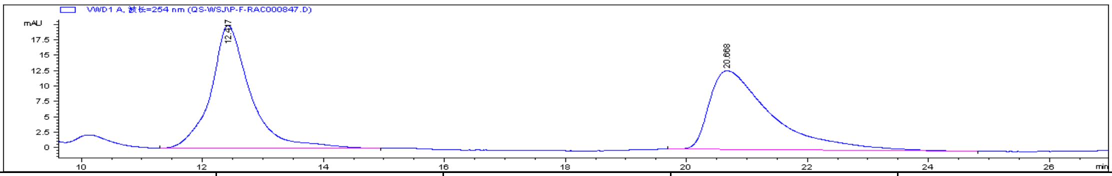

Entry	Retention Time	Area	Height	%Area
1	12.417	898.9	19.9	49.603
2	20.668	913.3	12.8	50.397

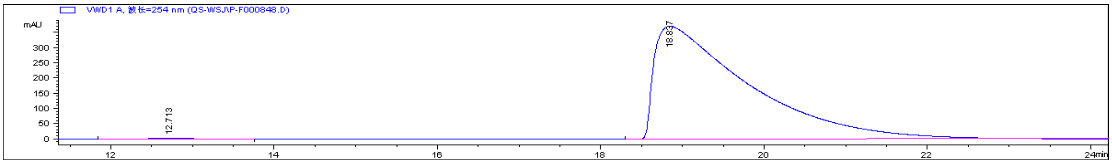

Entry	Retention Time	Area	Height	%Area
1	12.71	138.7	3.3	0.458
2	18.839	30133.5	371.9	99.542

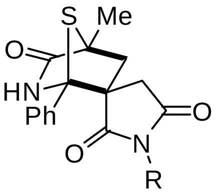

R = 4-ClPh

3c, white solid, Mp 122.0−122.5 °C; 40.0 mg (0.1 mmol), 97% yield; 90% *ee*; $[\alpha]_D^{26}$ 61.2 (*c* 1.0, $CHCl_3$); ^{1}H NMR (300 MHz, $CDCl_3$) δ 7.41–7.49 (m, *J* = 11.5, 10.1, 7.4 Hz, 3H), 7.37 (d, *J* = 8.7 Hz, 2H), 7.17–7.21 (m, 3H), 7.02 (d, *J* = 8.8 Hz, 2H), 3.05 (d, *J* = 18.9 Hz, 1H), 2.97 (d, *J* = 13.0 Hz, 1H), 2.88 (d, *J* = 19.0 Hz, 1H), 2.42 (d, *J* = 13.0 Hz, 1H), 1.74 (s, 3H); ^{13}C NMR (75 MHz, $CDCl_3$) δ 177.4, 176.1, 172.4, 134.4, 130.7, 130.4, 129.8, 129.3, 129.2, 127.2, 126.2, 84.2, 61.5, 60.5, 51.2, 44.1, 14.9; HRMS (ESI) m/z 413.0727 (M+H$^+$), calc. for $C_{21}H_{18}N_2O_3SCl$ 413.0727.

The *ee* was determined by HPLC analysis. CHIRALPAK IB-3 (4.6 mm i.d. x 250 mm); Hexane/2-propanol = 70/30; flow rate 1.0 mL/min; 25 °C; 254 nm; retention time: 14.1min (minor) and 20.3 min (major).

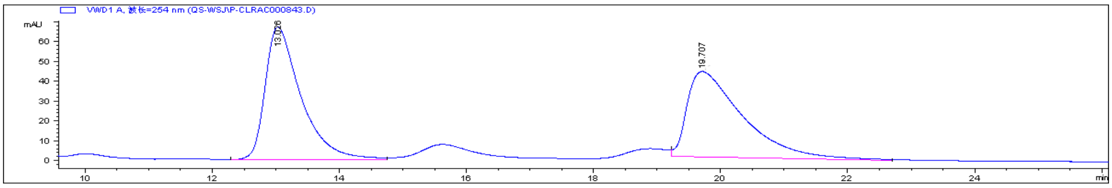

Entry	Retention Time	Area	Height	%Area
1	13.026	2644.8	67	49.586
2	19.707	2689	43.3	50.414

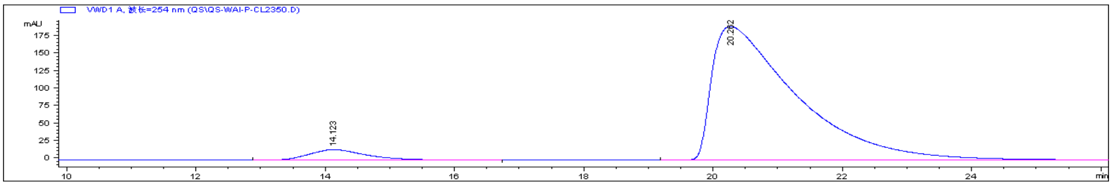

Entry	Retention Time	Area	Height	%Area
1	14.123	940.2	15.1	5.119
2	20.262	17424.8	190.9	94.881

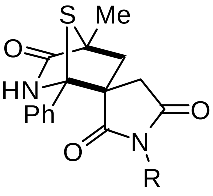

R = 4-BrPh

3d, white solid, Mp 204.9−205.8 °C; 43.8 mg (0.1 mmol), 96% yield; 96% *ee*; $[\alpha]_D^{26}$ 143.8 (*c* 1.0, $CHCl_3$); ^{1}H NMR (300 MHz, $CDCl_3$) δ 7.53 (d, *J* = 8.7 Hz, 2H), 7.39–7.47 (m, 3H), 7.19 (d, *J* = 7.0 Hz, 2H), 7.04 (s, 1H), 6.96 (d, *J* = 8.7 Hz, 2H), 3.05 (d, *J* = 18.9 Hz, 1H), 2.97 (d, *J* = 13.0 Hz, 1H), 2.88 (d, *J* = 18.9 Hz, 1H), 2.43 (d, *J* = 13.1 Hz, 1H), 1.75 (s, 3H); ^{13}C NMR (75 MHz, $CDCl_3$) δ 177.5, 176.0, 172.3, 132.2, 130.7, 130.4, 130.3, 129.3, 127.5, 126.2, 122.5, 84.2, 61.6, 60.5, 51.2, 44.1, 14.8; HRMS (ESI) m/z 457.0226 (M+H$^+$), calc. for $C_{21}H_{18}N_2O_3SBr$ 457.0222.

The *ee* was determined by HPLC analysis. CHIRALPAK IB-3 (4.6 mm i.d. x 250 mm); Hexane/2-propanol = 70/30; flow rate 1.0 mL/min; 25 °C; 254 nm; retention time: 13.9min (minor) and 19.6 min (major).

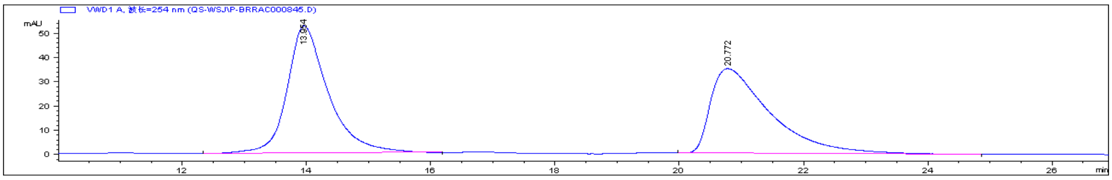

Entry	Retention Time	Area	Height	%Area
1	13.954	2422.6	52.3	50.981
2	20.772	2329.4	34.8	49.019

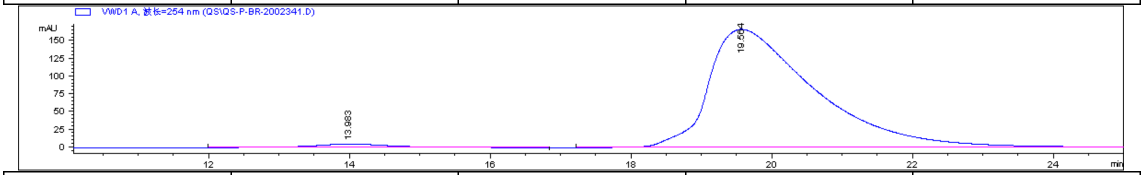

Entry	Retention Time	Area	Height	%Area
1	13.983	380.4	5.1	2.124
2	19.564	17527	166.6	97.876

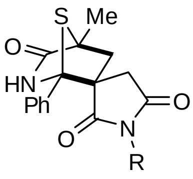

R = 3-ClPh

3e, white solid, Mp 133.1−134.8 °C; 39.6 mg (0.1 mmol), 96% yield; 91% *ee*; $[\alpha]_D^{26}$ 26.2 (*c* 1.0, $CHCl_3$); ^{1}H NMR (300 MHz, $CDCl_3$) δ 7.51–7.40 (m, 3H), 7.34 (dd, *J* = 3.9, 1.5 Hz, 2H), 7.21 (dd, *J* = 8.0, 1.3 Hz, 2H), 7.13 (s, 1H), 7.08 (s, 1H), 6.99–6.96 (m, 1H), 3.06 (d, *J* = 18.9 Hz, 1H), 2.98 (d, *J* = 13.0 Hz, 1H), 2.88 (d, *J* = 19.0 Hz, 1H), 2.42 (d, *J* = 13.0 Hz, 1H), 1.75 (s, 3H); ^{13}C NMR (75 MHz, $CDCl_3$) δ 177.4, 176.0, 172.2, 134.6, 132.4, 130.7, 130.5, 130.0, 129.3, 128.9, 126.3, 126.2, 124.2, 84.2, 61.6, 60.5, 51.2, 44.1, 14.9; HRMS (ESI) m/z 413.0723 (M+H$^+$), calc. for $C_{21}H_{18}N_2O_3SCl$ 413.0727.

The *ee* was determined by HPLC analysis. CHIRALPAK IB-3 (4.6 mm i.d. x 250 mm); Hexane/2-propanol = 70/30; flow rate 1.0 mL/min; 25 °C; 254 nm; retention time: 12.2min (minor) and 17.7 min (major).

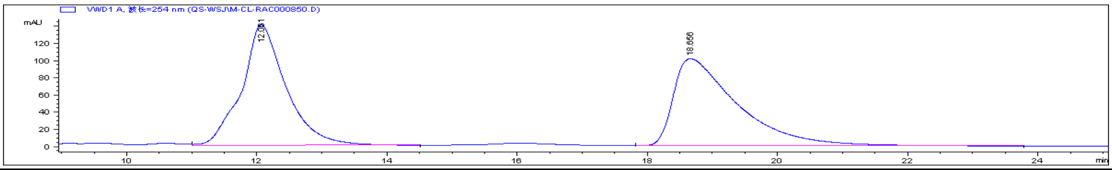

Entry	Retention Time	Area	Height	%Area
1	12.061	6723.3	140.6	49.211
2	18.656	6938.9	101.4	50.789

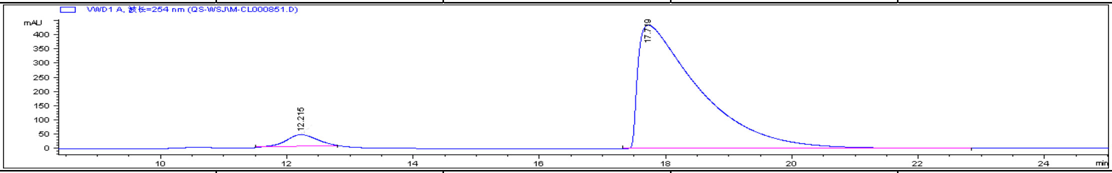

Entry	Retention Time	Area	Height	%Area
1	12.215	1452.4	42.3	4.734
2	17.719	29226.8	434.3	95.266

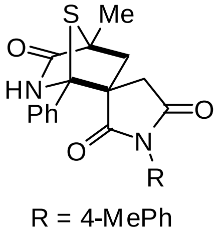

3f, white solid, Mp 228.0–229.8 °C; 37.3 mg (0.1 mmol), 95% yield; 92% *ee*; $[\alpha]_D^{26}$ 44.6 (*c* 1.0, $CHCl_3$); ^{1}H NMR (300 MHz, $CDCl_3$) δ 7.38–7.49 (m, 3H), 7.19–7.23 (m, 4H), 6.99 (s, 1H), 6.93 (d, *J* = 8.3 Hz, 2H), 3.03 (d, *J* = 18.9 Hz, 1H), 2.96 (d, *J* = 13.0 Hz, 1H), 2.87 (d, *J* = 18.9 Hz, 1H), 2.43 (d, *J* = 13.0 Hz, 1H), 2.36 (s, 3H), 1.75 (s, 3H); ^{13}C NMR (75 MHz, $CDCl_3$) δ 177.4, 176.6, 172.9, 138.8, 130.9, 130.3, 129.7, 129.3, 128.8, 126.3, 125.9, 84.2, 61.4, 60.5, 51.5, 44.1, 21.2, 14.9; HRMS (ESI) m/z 393.1270 ($M+Na^+$), calc. for $C_{22}H_{21}N_2O_3S$ 393.1273.

The *ee* was determined by HPLC analysis. CHIRALPAK IB-3 (4.6 mm i.d. x 250 mm); Hexane/2-propanol = 70/30; flow rate 1.0 mL/min; 25 °C; 254 nm; retention time: 15.3min (major) and 24.3 min (minor).

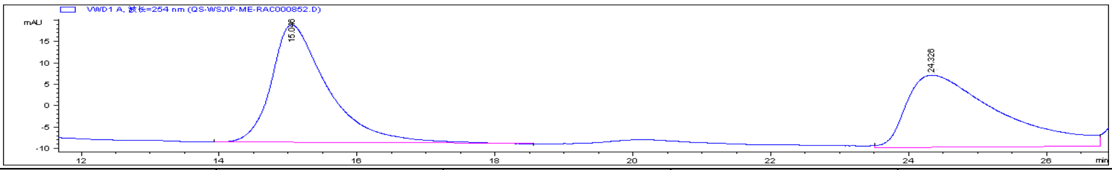

Entry	Retention Time	Area	Height	%Area
1	15.046	1567	27.5	49.703
2	24.326	1585.7	17	50.297

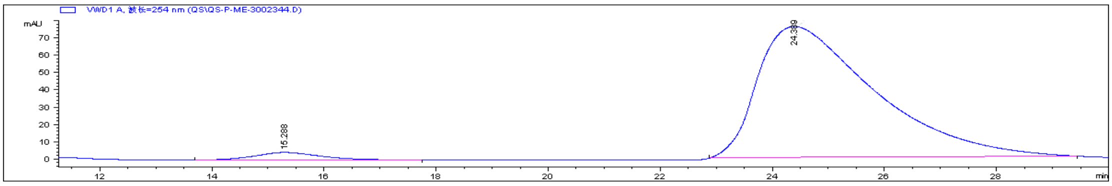

Entry	Retention Time	Area	Height	%Area
1	15.288	359.4	4.4	3.400
2	24.389	10211.3	74.2	96.600

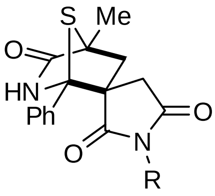

R = 3-MePh

3g, white solid, Mp 123.9−124.8 °C; 36.8 mg (0.1 mmol), 94% yield; 93% *ee*; $[\alpha]_D^{26}$ 100.1 (*c* 1.0, $CHCl_3$); ^1H NMR (300 MHz, $CDCl_3$) δ 7.48–7.40 (m, 3H), 7.30 (t, *J* = 7.8 Hz, 1H), 7.24–7.17 (m, *J* = 13.3, 7.2 Hz, 3H), 6.85–6.82 (m, *J* = 9.6 Hz, 3H), 3.04 (d, *J* = 18.9 Hz, 1H), 2.97 (d, *J* = 13.0 Hz, 1H), 2.87 (d, *J* = 18.9 Hz, 1H), 2.44 (d, *J* = 13.0 Hz, 1H), 2.36 (s, 3H), 1.76 (s, 3H); ^{13}C NMR (75 MHz, $CDCl_3$) δ 177.4, 176.6, 172.8, 139.1, 131.3, 130.9, 130.3, 129.6, 129.3, 128.9, 126.7, 126.3, 123.1, 84.2, 61.4, 60.5, 51.5, 44.1, 21.3, 14.9; HRMS (ESI) m/z 393.1272 ($M+H^+$), calc. for $C_{22}H_{21}N_2O_3S$ 393.1273.

The ee was determined by HPLC analysis. CHIRALPAK IB-3 (4.6 mm i.d. x 250 mm); Hexane/2-propanol = 70/30; flow rate 1.0 mL/min; 25 °C; 254 nm; retention time: 11.1 min (minor) and 21.5 min (major).

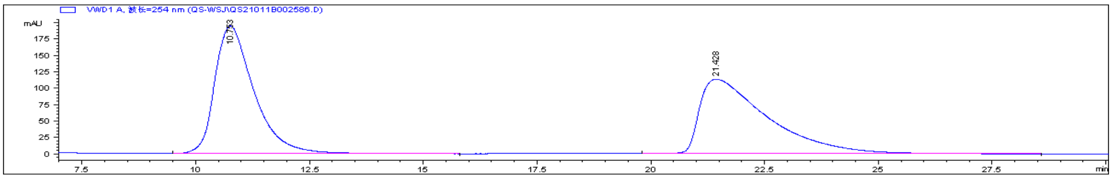

Entry	Retention Time	Area	Height	%Area
1	10.753	11634.4	194.6	49.556
2	21.428	11843	113.6	50.444

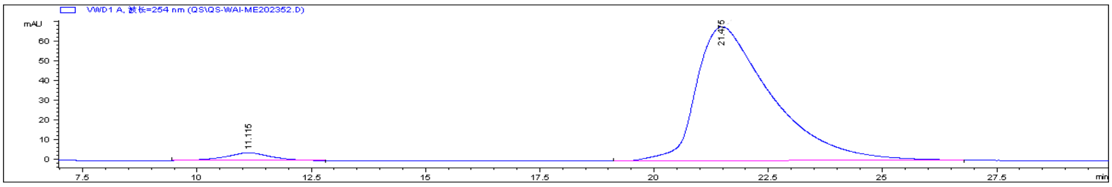

Entry	Retention Time	Area	Height	%Area
1	11.115	274	4	3.362
2	21.475	7877.2	68.4	96.638

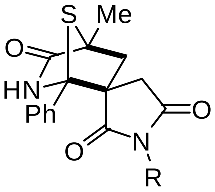

R = 4-OMePh

3h, white solid, Mp 211.1−211.9 °C; 38.0 mg (0.1 mmol), 93% yield; 92% *ee*; $[\alpha]_D^{26}$ 59.5 (*c* 1.0, $CHCl_3$); ^1H NMR (300 MHz, $CDCl_3$) δ 7.40–7.49 (m, 3H), 7.22 (d, *J* = 6.7 Hz, 2H), 6.90–6.98 (td, *J* = 9.1, 4.2

Hz, 5H), 3.80 (s, 3H), 3.03 (d, *J* = 18.9 Hz, 1H), 2.96 (d, *J* = 13.0 Hz, 1H), 2.86 (d, *J* = 18.9 Hz, 1H), 2.43 (d, *J* = 13.0 Hz, 1H), 1.75 (s, 3H); ^{13}C NMR (75 MHz, $CDCl_3$) δ 177.4, 176.7, 173.0, 159.5, 130.9, 130.4, 129.3, 127.2, 126.3, 124.0, 114.4, 84.2, 61.3, 60.5, 55.5, 51.5, 44.1, 14.9; HRMS (ESI) m/z 409.1218 (M+H$^+$), calc. for $C_{22}H_{21}N_2O_4S$ 409.1222.

The *ee* was determined by HPLC analysis. CHIRALPAK IB-3 (4.6 mm i.d. x 250 mm); Hexane/2-propanol = 70/30; flow rate 1.0 mL/min; 25°C; 254 nm; retention time: 18.8 min (major) and 30.3 min (minor).

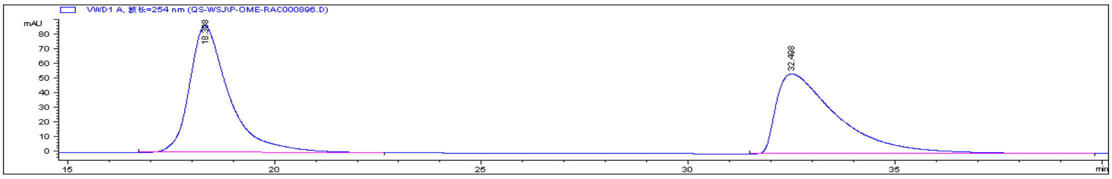

Entry	Retention Time	Area	Height	%Area
1	18.308	5775.9	86.8	50.527
2	32.498	5655.3	54.8	49.473

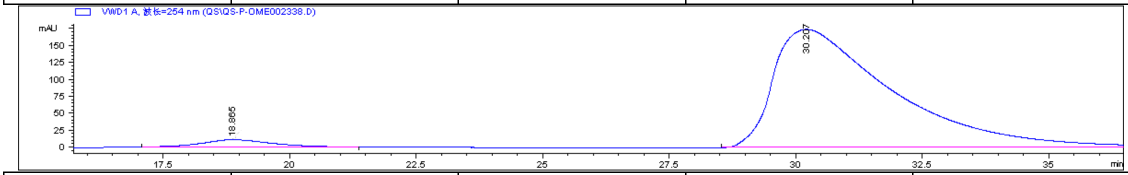

Entry	Retention Time	Area	Height	%Area
1	18.865	1051.2	10.8	3.535
2	30.207	28688.5	174.2	96.465

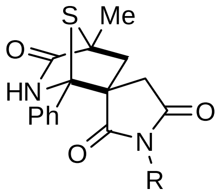

R = 1-naphthyl

3i, white solid, Mp 214.3−215.8 °C; 42.1 mg (0.1 mmol), 98% yield; 94% *ee*; $[\alpha]_D^{26}$ 43.7 (*c* 1.0, $CHCl_3$); ^{1}H NMR (300 MHz, Acetone) δ 8.32 (s, 1H), 7.99–7.91 (m, *J* = 13.3, 7.9 Hz, 3H), 7.66 (s, 1H), 7.58–7.51 (m, 7H), 7.24 (dd, *J* = 8.8, 1.8 Hz, 1H), 3.25–2.93 (m, 3H), 2.62 (d, *J* = 13.2 Hz, 1H), 1.67 (s, 3H); ^{13}C NMR (75 MHz, DMSO) δ 177.6, 176.0, 173.3, 132.6, 132.3, 131.0, 130.0, 129.7, 128.8, 128.7, 128.0, 127.8, 127.1, 127.0, 125.3, 124.3, 84.9, 61.9, 59.9, 49.4, 43.7, 14.9; HRMS (ESI) m/z 429.1276 (M+H$^+$), calc. for $C_{25}H_{21}N_2O_3S$ 429.1273.

The *ee* was determined by HPLC analysis. CHIRALPAK IB-3 (4.6 mm i.d. x 250 mm); Hexane/2-propanol = 70/30; flow rate 1.0 mL/min; 25 °C; 254 nm; retention time: 16.4min (minor) and 25.3 min (major).

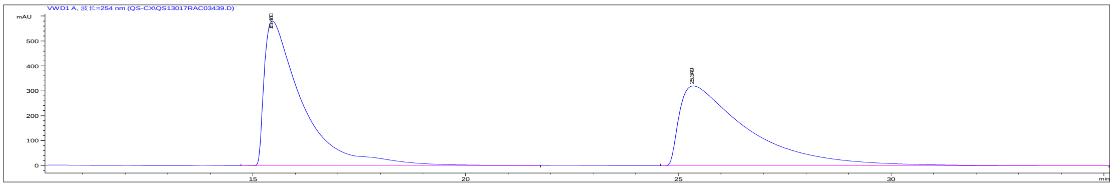

Entry	Retention Time	Area	Height	%Area
1	15.46	35951.5	580.3	50.124
2	25.349	35773.2	320	49.876

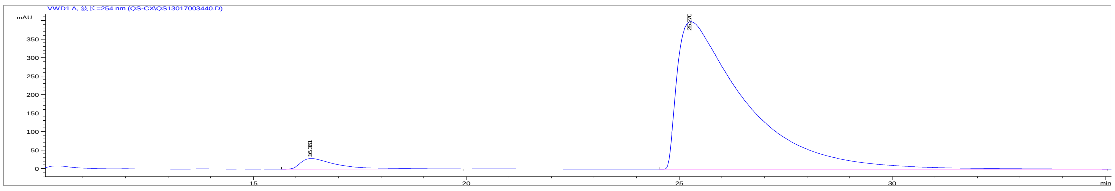

Entry	Retention Time	Area	Height	%Area
1	16.361	1672.8	28.6	3.635
2	25.27	44350.7	398.8	96.365

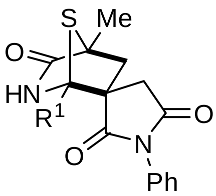

R^1 = 4-ClPh

3j, white solid, Mp 112.4−112.9 °C; 36.3 mg (0.1 mmol), 88% yield; 92% *ee*; $[\alpha]_D^{26}$ 89.7 (*c* 1.0, $CHCl_3$); ^1H NMR (300 MHz, $CDCl_3$) δ 7.47–7.38 (m, 5H), 7.17 (d, *J* = 8.6 Hz, 2H), 7.09–7.06 (m, 2H), 6.89 (s, 1H), 3.03–2.87 (m, 3H), 2.45 (d, *J* = 13.0 Hz, 1H), 1.76 (s, 3H); ^{13}C NMR (75 MHz, $CDCl_3$) δ 177.2, 176.5, 172.4, 136.7, 131.3, 129.6, 129.5, 129.2, 128.9, 127.8, 126.0, 83.5, 61.3, 60.8, 51.8, 44.1, 14.9; HRMS (ESI) m/z 413.0729 ($M+H^+$), calc. for $C_{21}H_{18}N_2O_3SCl$ 413.0727.

The *ee* was determined by HPLC analysis. CHIRALPAK IB-3 (4.6 mm i.d. x 250 mm); Hexane/2-propanol = 70/30; flow rate 1.5 mL/min; 25 °C; 254 nm; retention time: 13.6min (minor) and 25.5 min (major).

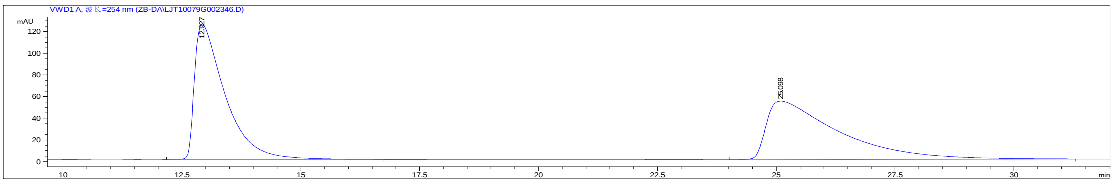

Entry	Retention Time	Area	Height	%Area
1	12.927	5578.5	124.8	49.588
2	25.098	5671.2	53.9	50.412

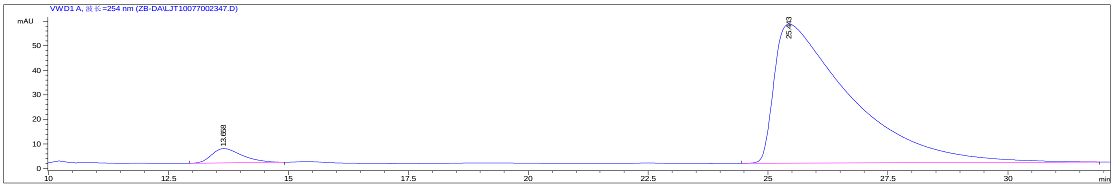

Entry	Retention Time	Area	Height	%Area
1	13.658	258.3	5.9	3.969
2	25.443	6249.4	56.7	96.031

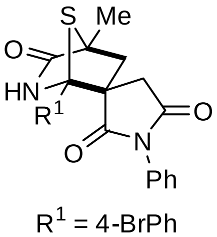

R^1 = 4-BrPh

3k, white solid, Mp 212.3−213.1 °C; 36.9 mg (0.1 mmol), 81% yield; 90% *ee*; $[\alpha]_D^{26}$ 118.3 (*c* 1.0, $CHCl_3$); ^{1}H NMR (300 MHz, $CDCl_3$) δ 7.53 (d, *J* = 8.5 Hz, 2H), 7.45–7.34 (m, 3H), 7.27 (s, 1H), 7.09 (d, *J* = 8.6 Hz, 2H), 7.07–7.04 (m, *J* = 8.0, 1.5 Hz, 2H), 3.02–2.86 (m, 3H), 2.43 (d, *J* = 13.0 Hz, 1H), 1.74 (s, 3H); ^{13}C NMR (75 MHz, $CDCl_3$) δ 177.3, 176.4, 172.5, 132.5, 131.3, 130.0, 129.2, 128.8, 128.0, 126.0, 124.7, 83.6, 61.3, 60.7, 51.7, 44.1, 14.8; HRMS (ESI) m/z 457.0223 (M+H$^+$), calc. for $C_{21}H_{18}N_2O_3SBr$ 457.0222.

The *ee* was determined by HPLC analysis. CHIRALPAK IB-3 (4.6 mm i.d. x 250 mm); Hexane/2-propanol = 70/30; flow rate 1.0 mL/min; 25 °C; 254 nm; retention time: 13.7 min (minor) and 25.4 min (major).

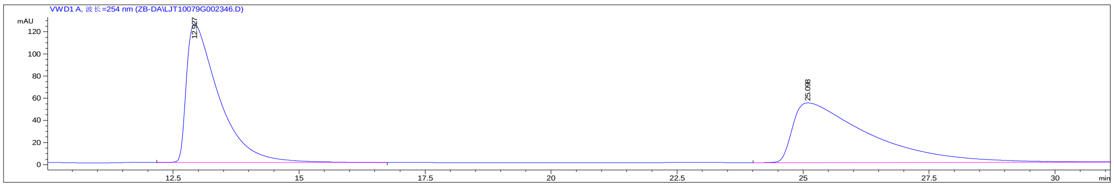

Entry	Retention Time	Area	Height	%Area
1	12.927	5578.5	124.8	49.588
2	25.098	5671.2	53.9	50.412

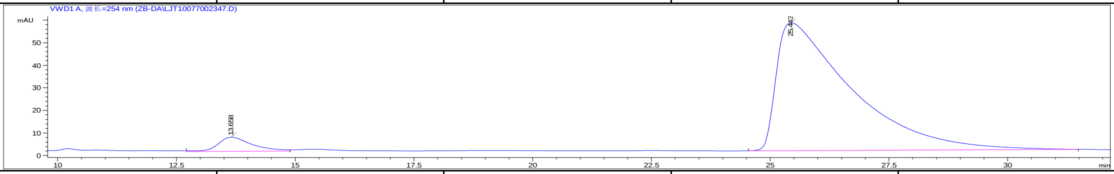

Entry	Retention Time	Area	Height	%Area
1	13.658	315.2	6.3	4.815
2	25.443	6230.6	56.7	95.185

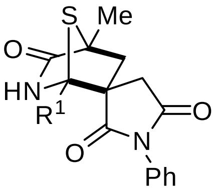

R^1 = *m*-FPh

3l, white solid, Mp 137.4−137.9 °C; 34.1 mg (0.1 mmol), 86% yield; 92% ee; $[\alpha]_D^{26}$ 50.2 (*c* 1.0, $CHCl_3$); ^{1}H NMR (300 MHz, $CDCl_3$) δ 7.45–7.34 (m, 4H), 7.21–7.16 (m, 2H), 7.08–6.97 (m, 4H), 3.06–2.87 (m, 3H), 2.44 (d, *J* = 13.0 Hz, 1H), 1.75 (s, *J* = 2.0 Hz, 3H); ^{13}C NMR (75 MHz, $CDCl_3$) δ 177.3, 176.3, 172.6, 164.4, 133.4, 133.3, 131.3, 131.1, 129.2, 128.9, 126.0, 122.2(two peaks), 117.7, 117.4, 114.1, 113.8, 83.4, 61.3, 60.7, 51.5, 44.0, 14.8; HRMS (ESI) m/z 397.1017 (M+H$^+$), calc. for $C_{21}H_{18}N_2O_3SF$ 397.1022.

The *ee* was determined by HPLC analysis. CHIRALPAK IB-3 (4.6 mm i.d. x 250 mm); Hexane/2-propanol = 70/30; flow rate 1.0 mL/min; 25 °C; 254 nm; retention time: 10.5 min (minor) and 16.9 min (major).

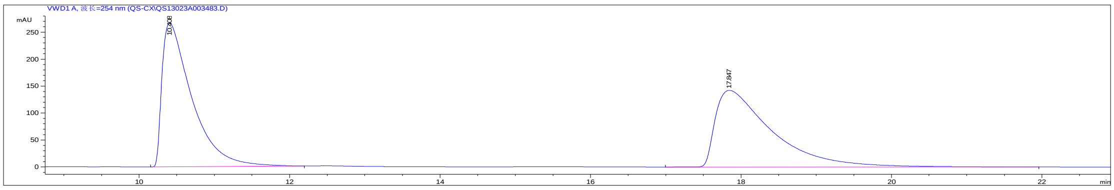

Entry	Retention Time	Area	Height	%Area
1	10.408	7329.2	266.4	49.623
2	17.847	7440.6	142.2	50.377

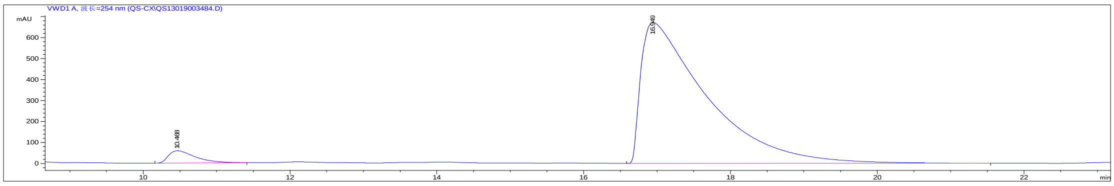

Entry	Retention Time	Area	Height	%Area
1	10.468	1464.2	58.2	3.395
2	16.949	41667.9	670.1	96.605

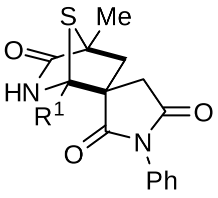

R^1 = 3-ClPh

3m, white solid, Mp 125.6−126.3 °C; 33.0 mg (0.1 mmol), 80% yield; 91% *ee*; $[\alpha]_D^{26}$ 101.2 (*c* 1.0, $CHCl_3$); ^{1}H NMR (300 MHz, $CDCl_3$) δ 7.47–7.35 (m, 5H), 7.25 (t, *J* = 1.9 Hz, 1H), 7.15–7.07 (m, 3H), 7.03 (s, 1H), 3.00 (dd, *J* = 15.9, 13.8 Hz, 2H), 2.90 (d, *J* = 19.0 Hz, 1H), 2.44 (d, *J* = 13.0 Hz, 1H), 1.76 (s, 3H); ^{13}C NMR (75 MHz, $CDCl_3$) δ 177.2, 176.3, 172.5, 135.6, 133.0, 131.2, 130.7, 130.6, 129.2, 128.9, 126.5, 126.2, 124.8, 83.4, 61.3, 60.7, 51.6, 44.1, 14.8; HRMS (ESI) m/z 413.0728 (M+H$^+$), calc. for $C_{21}H_{18}N_2O_3SCl$ 413.0727.

The *ee* was determined by HPLC analysis. CHIRALPAK IB-3 (4.6 mm i.d. x 250 mm); Hexane/2-propanol = 70/30; flow rate 1.0 mL/min; 25 °C; 254 nm; retention time: 11.9 min (minor) and 19.6 min (major).

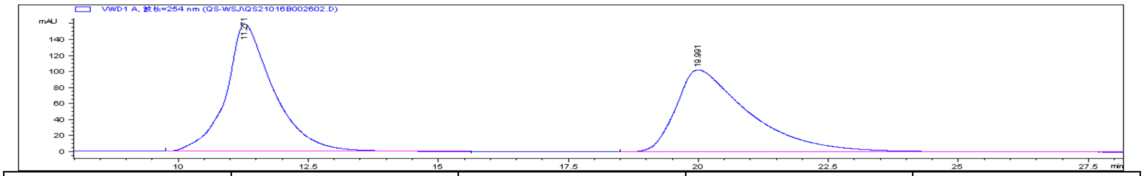

Entry	Retention Time	Area	Height	%Area
1	11.271	10139.6	159	50.176
2	19.991	10068.6	102.4	49.824

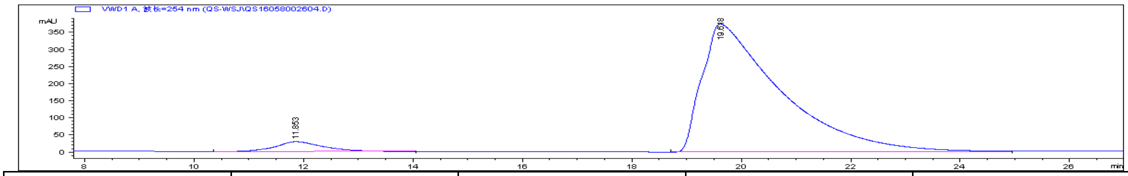

Entry	Retention Time	Area	Height	%Area
1	11.853	1847.2	29.1	4.677
2	19.618	37649.2	374	95.323

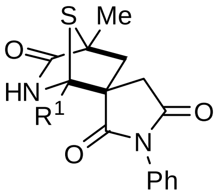

R^1 = p-MePh

3n, white solid, Mp 221.5−222.7 °C; 38.4 mg (0.1 mmol), 98% yield; 90% *ee*; $[\alpha]^{26}_{D}$ 154.0 (*c* 1.0, $CHCl_3$); ^{1}H NMR (300 MHz, $CDCl_3$) δ 7.45–7.34 (m, 3H), 7.21 (d, *J* = 8.0 Hz, 2H), 7.11–7.06 (m, 4H), 6.88 (s, 1H), 3.04 (d, *J* = 18.9 Hz, 1H), 2.96 (d, *J* = 13.0 Hz, 1H), 2.87 (d, *J* = 18.9 Hz, 1H), 2.43 (d, *J* = 13.0 Hz, 1H), 2.37 (s, 3H), 1.74 (s, 3H); ^{13}C NMR (75 MHz, $CDCl_3$) δ 177.4, 176.6, 172.8, 140.6, 131.5, 129.9, 129.1, 128.7, 127.9, 126.2, 126.1, 84.2, 61.3, 60.5, 51.7, 44.2, 21.2, 14.9; HRMS (ESI) m/z 393.1270 ($M+H^+$), calc. for $C_{22}H_{21}N_2O_3S$ 393.1273.

The *ee* was determined by HPLC analysis. CHIRALPAK IB-3 (4.6 mm i.d. x 250 mm); Hexane/2-propanol = 70/30; flow rate 1.0 mL/min; 25 °C; 254 nm; retention time: 14.6min (minor) and 22.1 min (major).

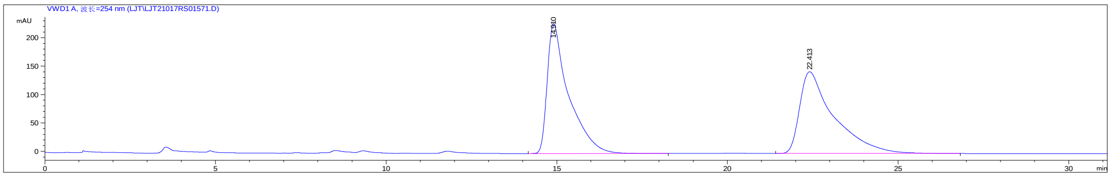

Entry	Retention Time	Area	Height	%Area
1	14.91	9967.4	229.2	49.779
2	22.413	10056	144	50.221

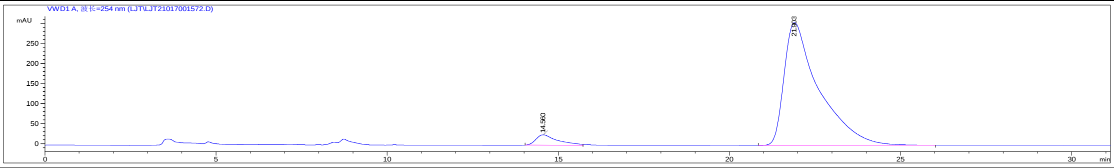

Entry	Retention Time	Area	Height	%Area
1	14.56	1119.4	25.6	4.934
2	21.903	21566.7	306.1	95.066

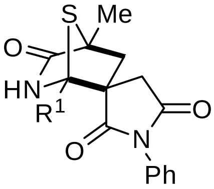

R^1 = 2-naphthyl

3o, white solid, Mp 218.8−220.0 °C; 37.2 mg (0.1 mmol), 87% yield; 92% *ee*; $[\alpha]^{26}_{D}$ 154.6 (*c* 1.0, $CHCl_3$); ^{1}H NMR (300 MHz, $CDCl_3$) δ 7.88 (d, *J* = 8.6 Hz, 2H), 7.77 (t, 1H), 7.70 (s, 1H), 7.59–7.56 (m, 2H), 7.39–7.36 (m, 3H), 7.29 (d, *J* = 1.9 Hz, 1H), 7.05 (s, 1H), 6.99 (dd, *J* =

7.6, 1.9 Hz, 2H), 3.12 (d, J = 18.9 Hz, 1H), 3.03 (d, J = 13.0 Hz, 1H), 2.90 (d, J = 18.8, 7.0 Hz, 1H), 2.50 (d, J = 13.0 Hz, 1H), 1.79 (s, 3H); ^{13}C NMR (75 MHz, $CDCl_3$) δ 177.3, 176.7, 172.7, 133.6, 132.7, 131.4, 129.4, 129.1, 128.8, 127.9, 127.7, 127.5, 126.4, 126.1, 122.7, 84.4, 61.5, 60.6, 51.8, 44.2, 14.9; HRMS (ESI) m/z 429.1275 (M+H$^+$), calc. for $C_{25}H_{21}N_2O_3S$ 429.1273.

The *ee* was determined by HPLC analysis. CHIRALPAK IB-3 (4.6 mm i.d. x 250 mm); Hexane/2-propanol = 70/30; flow rate 1.0 mL/min; 25 °C; 254 nm; retention time: 13.6 min (minor) and 25.5 min (major).

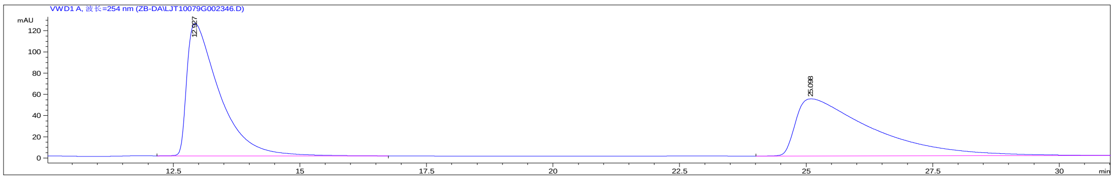

Entry	Retention Time	Area	Height	%Area
1	12.927	5578.5	124.8	49.588
2	25.098	5671.2	53.9	50.412

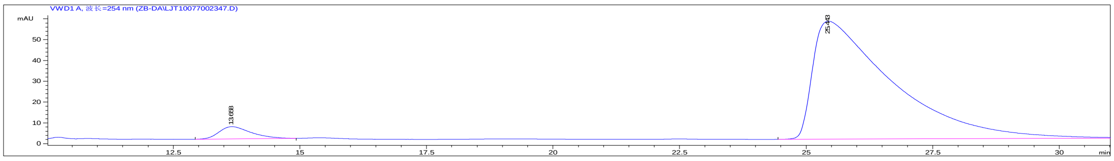

Entry	Retention Time	Area	Height	%Area
1	13.658	258.3	5.9	3.969
2	25.443	6249.4	56.7	96.031

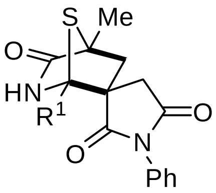

R^1 = 2-furyl

3p, white solid, Mp 99.1−100.0 °C; 34.6 mg (0.1 mmol), 94% yield; 96% *ee*; $[\alpha]_D^{26}$ 85.5 (c 1.0, $CHCl_3$); ^{1}H NMR (300 MHz, $CDCl_3$) δ 7.47–7.38 (m, 4H), 7.18–7.15 (m, 2H), 6.65 (s, 1H), 6.53 (d, J = 3.3 Hz, 1H), 6.45–6.43 (dd, J = 3.3, 1.9 Hz, 1H), 3.20 (d, J = 18.8 Hz, 1H), 2.99 (d, 1H), 2.90 (d, J = 13.1 Hz, 1H), 2.38 (d, J = 13.0 Hz, 1H), 1.73 (s, 3H); ^{13}C NMR (75 MHz, $CDCl_3$) δ 176.6, 176.0, 172.9, 145.0, 144.2, 131.6, 129.1, 128.7, 126.0, 111.4, 111.3, 77.1, 61.8, 60.9, 50.6, 44.5, 14.8; HRMS (ESI) m/z 369.0904 (M+H$^+$), calc. for $C_{19}H_{17}N_2O_4S$ 369.0909.

The *ee* was determined by HPLC analysis. CHIRALPAK IB-3 (4.6 mm i.d. x 250 mm); Hexane/2-propanol = 70/30; flow rate 1.0 mL/min; 25 °C; 254 nm; retention time: 13.0min (minor) and 18.3 min (major).

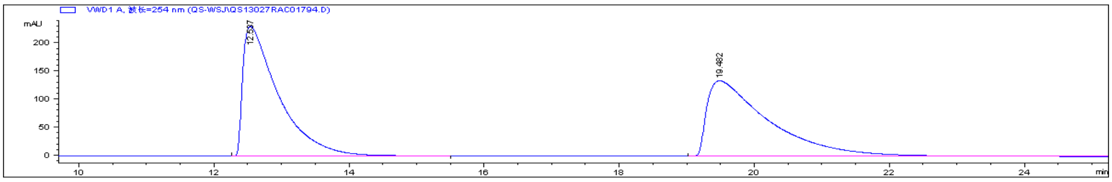

Entry	Retention Time	Area	Height	%Area
1	12.537	8505.6	231.3	49.638
2	19.482	8629.6	134.4	50.362

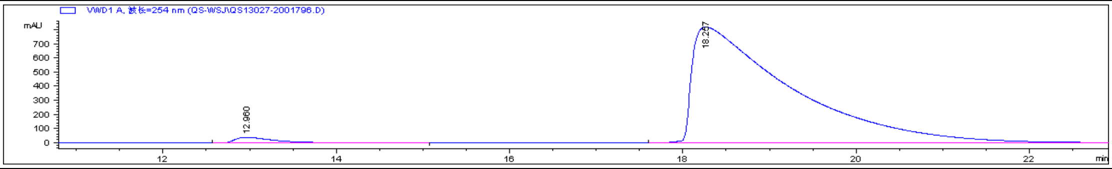

Entry	Retention Time	Area	Height	%Area
1	12.962	1259.8	39.1	1.891
2	18.26	65350.4	822.1	98.109

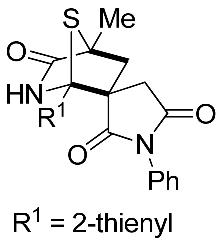

3q, white solid, Mp 98.6−100.1 °C; 36.9 mg (0.1 mmol), 96% yield; 91% *ee*; $[\alpha]_D^{26}$ 57.2 (*c* 1.0, $CHCl_3$); ^1H NMR (300 MHz, $CDCl_3$) δ 7.46–7.35 (m, 4H), 7.19–7.16 (m, 2H), 7.07–7.03 (m, 2H), 6.80 (s, 1H), 3.19 (d, *J* = 19.0 Hz, 1H), 2.94 (dd, *J* = 16.0, 6.9 Hz, 2H), 2.45 (d, *J* = 13.0 Hz, 1H), 1.74 (s, 3H); ^{13}C NMR (75 MHz, $CDCl_3$) δ 176.7, 176.2, 172.8, 133.2, 131.5, 129.1, 128.7, 127.9, 127.8, 127.6, 126.1, 79.8, 61.7, 61.6, 51.9, 44.6, 14.8; HRMS (ESI) m/z 385.0684 $(M+H^+)$, calc. for $C_{19}H_{17}N_2O_3S_2$ 385.0681.

The *ee* was determined by HPLC analysis. CHIRALPAK IB-3 (4.6 mm i.d. x 250 mm); Hexane/2-propanol = 70/30; flow rate 1.0 mL/min; 25 °C; 254 nm; retention time: 16.2 min (minor) and 29.0 min (major).

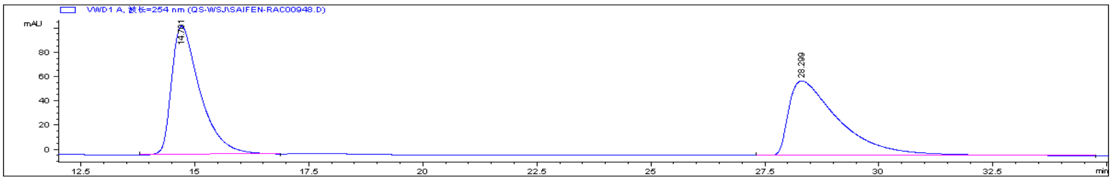

Entry	Retention Time	Area	Height	%Area
1	14.701	4597.2	105.8	49.282
2	28.299	4731.2	61.5	50.718

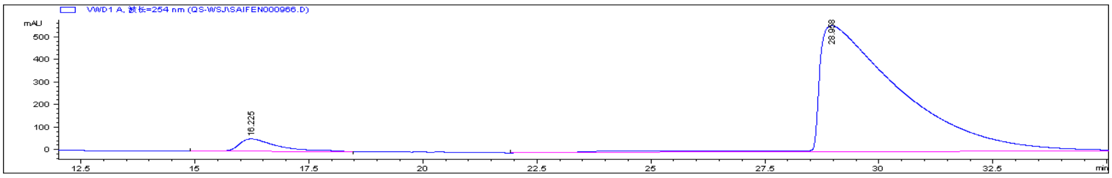

Entry	Retention Time	Area	Height	%Area
1	16.225	3221.7	54.7	4.280
2	28.958	72044.7	560.1	95.720

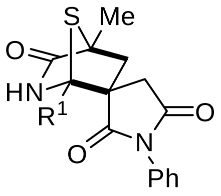

R1 = 2-quinolyl

3r, white solid, Mp 230.1−230.7 °C; 39.9 mg (0.1 mmol), 93% yield; 94% *ee*; $[\alpha]_D^{26}$ 112.1 (*c* 1.0, $CHCl_3$); ^{1}H NMR (300 MHz, $CDCl_3$) δ 8.27 (d, *J* = 8.5 Hz, 1H), 7.86–7.83 (m, *J* = 8.3 Hz, 2H), 7.72–7.57 (m, 3H), 7.45–7.34 (m, 3H), 7.25–7.22 (m, 2H), 6.76 (s, 1H), 3.41 (d, *J* = 18.7 Hz, 1H), 2.99 (dd, *J* = 15.8, 10.9 Hz, 2H), 2.47 (d, *J* = 13.0 Hz, 1H), 1.81 (s, 3H); ^{13}C NMR (75 MHz, $CDCl_3$) δ 177.3, 177.1, 174.1, 151.7, 146.7, 138.3, 132.0, 130.7, 129.3, 129.0, 128.6, 128.0, 127.8, 127.7, 126.3, 120.5, 84.2, 62.0, 59.6, 52.6, 45.7, 15.1; HRMS (ESI) m/z 430.1224 (M+H$^+$), calc. for $C_{24}H_{20}N_3O_3S$ 430.1225.

The *ee* was determined by HPLC analysis. CHIRALPAK IC (4.6 mm i.d. x 250 mm); Hexane/2-propanol = 70/30; flow rate 1.0 mL/min; 25 °C; 254 nm; retention time: 16.7min (minor) and 20.5 min (major).

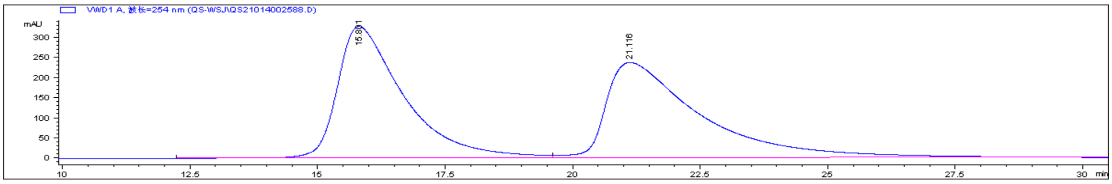

Entry	Retention Time	Area	Height	%Area
1	15.801	29544.8	326.7	49.142
2	21.116	30576.9	236.7	50.858

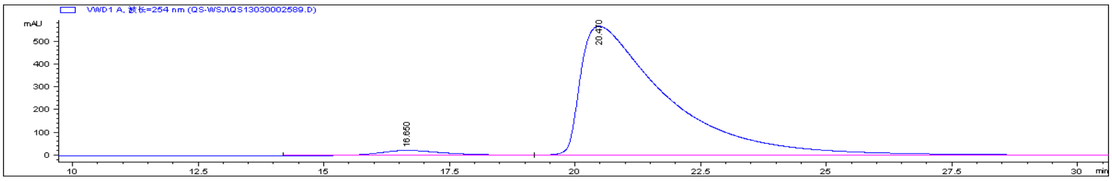

Entry	Retention Time	Area	Height	%Area
1	16.65	2136.9	22.6	3.032
2	20.47	68347	569.2	96.968

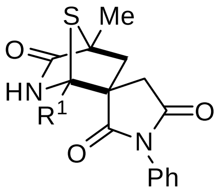

R1 = 2-pyridyl

3s, white solid, Mp 143.2−144.6 °C; 36.0 mg (0.1 mmol), 95% yield; 99% *ee*; $[\alpha]_D^{26}$ 98.1 (*c* 1.0, $CHCl_3$); ^{1}H NMR (300 MHz, $CDCl_3$) δ 8.71 (d, *J* = 4.2 Hz, 1H), 8.58 (s, 1H), 7.59–7.56 (m, 1H), 7.52 (s, 1H), 7.44–7.33 (m, 4H), 7.07 (dd, *J* = 8.1, 1.3 Hz, 2H), 2.99 (d, *J* = 13.1 Hz, 1H), 2.94 (d, *J* = 1.6 Hz, 2H), 2.44 (d, *J* = 13.0 Hz, 1H), 1.76 (s, 3H); ^{13}C NMR (75 MHz, $CDCl_3$) δ 177.5, 176.1, 172.3, 151.5, 147.5, 134.2, 131.2, 129.2, 128.8, 127.1, 126.0, 123.6, 82.3, 61.4, 60.8, 51.4, 44.0, 14.8; HRMS (ESI) m/z 380.1060 (M+H$^+$), calc. for $C_{20}H_{18}N_3O_3S$ 380.1059.

The ee was determined by HPLC analysis. CHIRALPAK IB-3 (4.6 mm i.d. x 250 mm);

Hexane/2-propanol = 70/30; flow rate 1.0 mL/min; 25 °C; 254 nm; retention time: 26.5min (minor) and 36.01 min (major).

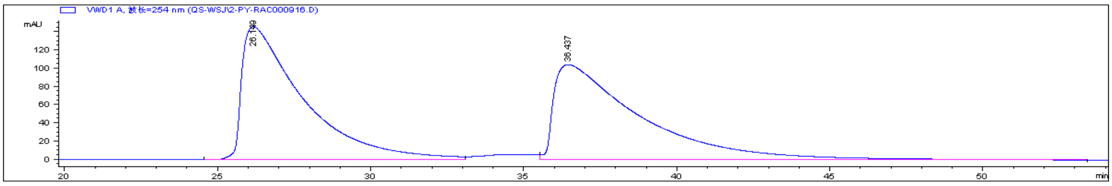

Entry	Retention Time	Area	Height	%Area
1	26.149	20358.3	145.8	49.740
2	36.437	20571.1	104.3	50.260

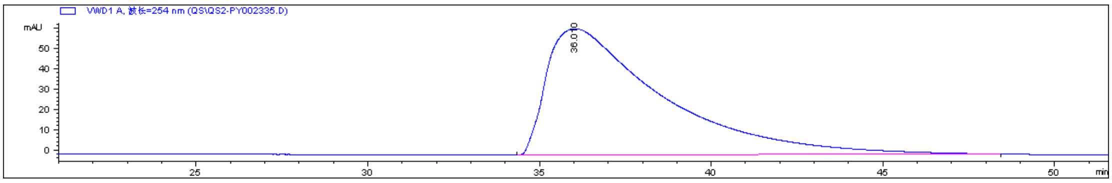

Entry	Retention Time	Area	Height	%Area
1	36.01	14595	62.1	100.000

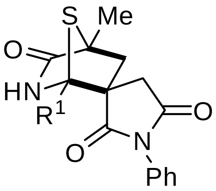

R1 = 3-pyridyl

3t, white solid, Mp 227.3–228.3 °C; 34.9 mg (0.1 mmol), 92% yield; 96% *ee*; $[\alpha]^{26}_{D}$ 116.3 (*c* 1.0, $CHCl_3$); ^1H NMR (300 MHz, $CDCl_3$) δ 8.53 (d, *J* = 4.4 Hz, 1H), 7.83–7.77 (td, *J* = 7.7, 1.4 Hz, 1H), 7.51–7.43 (m, *J* = 15.0, 7.8 Hz, 3H), 7.40–7.32 (m, 2H), 7.28 (s, 1H), 6.73 (s, 1H), 3.05 (d, *J* = 18.7 Hz, 1H), 2.95–2.89 (m, 2H), 2.42 (d, *J* = 12.9 Hz, 1H), 1.78 (s, 3H); ^{13}C NMR (75 MHz, $CDCl_3$) δ 176.7, 173.2, 151.2, 149.3, 137.8, 132.0, 129.0, 128.5, 126.1, 124.7, 123.2, 84.0, 61.9, 59.8, 51.8, 44.6, 15.0; HRMS (ESI) m/z 380.1062 ($M+H^+$), calc. for $C_{20}H_{18}N_3O_3S$ 380.1069.

The *ee* was determined by HPLC analysis. CHIRALPAK IF (4.6 mm i.d. x 250 mm); Hexane/2-propanol = 70/30; flow rate 1.0 mL/min; 25 °C; 254 nm; retention time: 17.1 min (minor) and 24.1 min (major).

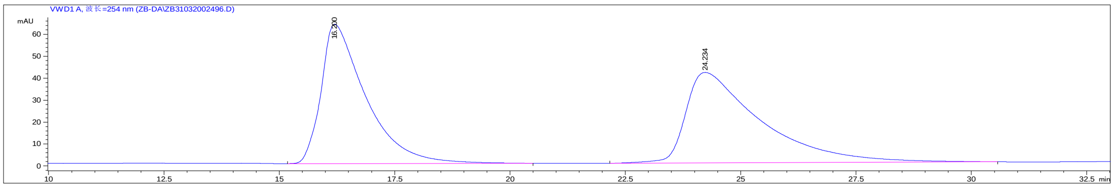

Entry	Retention Time	Area	Height	%Area
1	16.2	4276.4	63.5	47.926
2	24.234	4646.5	41.3	52.074

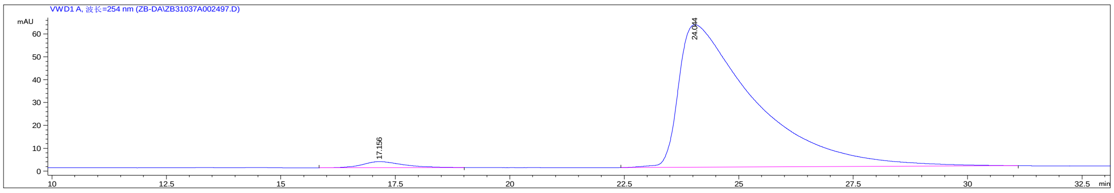

Entry	Retention Time	Area	Height	%Area
1	17.156	162.9	2.7	2.201
2	24.044	7239.6	62.4	97.799

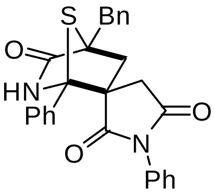

3u, white solid, Mp 110.2−111.9 °C; 40.9 mg (0.1 mmol), 90% yield; 96% *ee*; $[\alpha]_D^{26}$ 16.9 (*c* 1.0, $CHCl_3$); ^1H NMR (300 MHz, $CDCl_3$) δ 7.46–7.37 (m, *J* = 7.9, 6.8, 4.7 Hz, 6H), 7.31–7.29 (m, 5H), 7.20 (d, *J* = 6.8 Hz, 2H), 7.05 (d, *J* = 6.9 Hz, 2H), 6.91 (s, 1H), 3.61 (d, *J* = 14.5 Hz, 1H), 3.28 (d, *J* = 14.5 Hz, 1H), 2.95–2.87 (m, *J* = 15.9, 8.5 Hz, 2H), 2.71 (d, *J* = 19.0 Hz, 1H), 2.42 (d, *J* = 13.0 Hz, 1H); ^{13}C NMR (75 MHz, $CDCl_3$) δ 176.8, 176.6, 172.5, 136.9, 131.4, 130.7, 130.4, 129.6, 129.3, 129.1, 128.8, 128.6, 127.3, 126.3, 126.0, 83.2, 66.8, 61.1, 49.0, 44.0, 34.9; HRMS (ESI) m/z 455.1427 ($M+H^+$), calc. for $C_{27}H_{23}N_2O_3S$ 455.1429.

The *ee* was determined by HPLC analysis. CHIRALPAK IB-3 (4.6 mm i.d. x 250 mm); Hexane/2-propanol = 70/30; flow rate 1.0 mL/min; 25 °C; 254 nm; retention time: 18.2 min (minor) and 42.7 min (major).

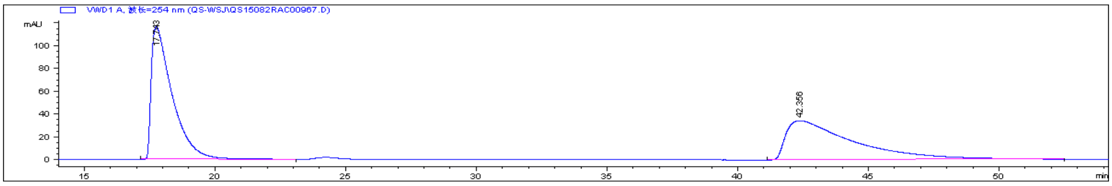

Entry	Retention Time	Area	Height	%Area
1	17.743	6568.8	116.5	51.351
2	42.356	6223.2	34.5	48.649

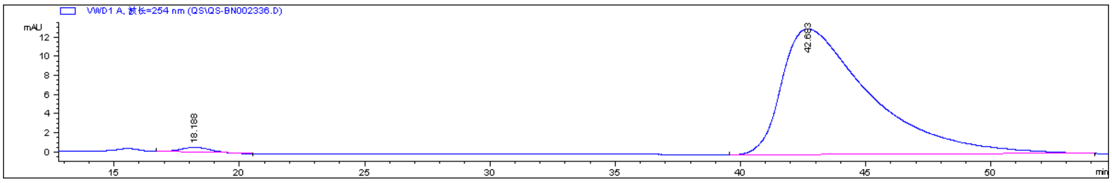

Entry	Retention Time	Area	Height	%Area
1	18.188	46.9	5.6E-1	1.428
2	42.683	3235.1	13.1	98.572

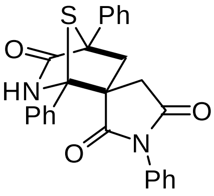

3v, white solid, Mp 216.3−217.4 °C; 37.4 mg (0.1 mmol), 85% yield; 94% *ee*; $[\alpha]_D^{26}$ −12.9 (*c* 1.0, $CHCl_3$); ^1H NMR (300 MHz, $CDCl_3$) δ

7.53–7.39 (m, 11H), 7.31–7.28 (m, *J* = 8.0, 1.4 Hz, 2H), 7.11–7.07 (m, *J* = 8.1, 1.4 Hz, 2H), 3.52 (d, *J* = 12.8 Hz, 1H), 3.14 (d, *J* = 18.9 Hz, 1H), 2.95 (d, *J* = 19.0 Hz, 1H), 2.87 (d, *J* = 12.9 Hz, 1H); ^{13}C NMR (75 MHz, $CDCl_3$) δ 176.3, 175.9, 172.6, 133.2, 131.5, 130.7, 130.5, 129.4, 129.1, 128.8, 128.6, 128.1, 126.2, 126.1, 83.0, 67.4, 61.6, 50.1, 44.3; HRMS (ESI) m/z 441.1270 ($M+H^+$), calc. for $C_{22}H_{21}N_2O_3S$ 441.1273.

The *ee* was determined by HPLC analysis. CHIRALPAK IB-3 (4.6 mm i.d. x 250 mm); Hexane/2-propanol = 70/30; flow rate 1.0 mL/min; 25 °C; 254 nm; retention time: 42.7min (major) and 88.1 min (minor).

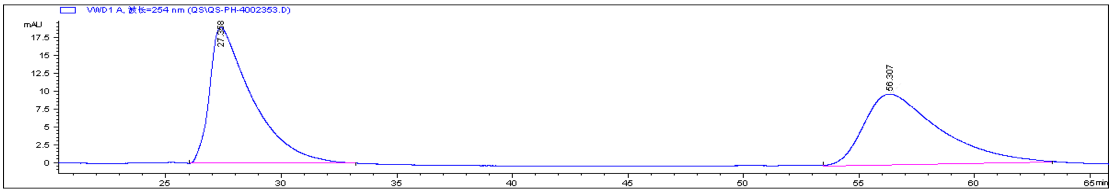

Entry	Retention Time	Area	Height	%Area
1	27.358	2428.9	19	51.665
2	56.307	2272.4	9.9	48.335

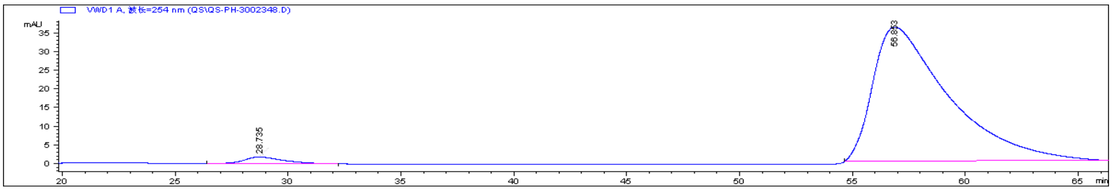

Entry	Retention Time	Area	Height	%Area
1	28.735	256.1	1.9	2.954
2	56.853	8414.8	35.9	97.046

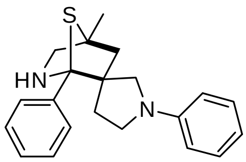

4, white solid, Mp 120.4−120.9 °C; 23.5 mg (0.1 mmol), 70% yield; 93% *ee*; $[\alpha]_D^{26}$ 77.3 (*c* 1.0, $CHCl_3$); ^{1}H NMR (300 MHz, $CDCl_3$) δ 7.24 (d, 2H), 7.12–7.02 (m, *J* = 16.4, 11.3, 7.0 Hz, 5H), 6.55 (t, *J* = 7.2 Hz, 1H), 6.18 (d, *J* = 8.0 Hz, 2H), 3.89 (d, *J* = 10.0 Hz, 1H), 3.46 (s, 1H), 3.19–2.97 (m, *J* = 37.8, 19.5, 6.6 Hz, 3H), 2.83–2.77 (m, *J* = 13.4, 4.4 Hz, 1H), 2.09–1.87 (m, 4H), 1.80 (s, 1H), 1.66 (s, 3H); ^{13}C NMR (75 MHz, $CDCl_3$) δ 146.7, 140.7, 140.5, 128.7, 127.8, 127.5, 114.7, 110.9, 70.8, 63.0, 54.7, 52.5, 47.5, 44.7, 43.6, 37.1, 27.8; HRMS (ESI) m/z 337.1739 ($M+H^+$), calc. for $C_{21}H_{25}N_2S$ 337.1738.

The *ee* was determined by HPLC analysis. CHIRALPAK IC (4.6 mm i.d. x 250 mm); Hexane/2-propanol = 70/30; flow rate 2.0 mL/min; 25 °C; 254 nm; retention time: 6.0 min (major) and 10.4 min (minor).

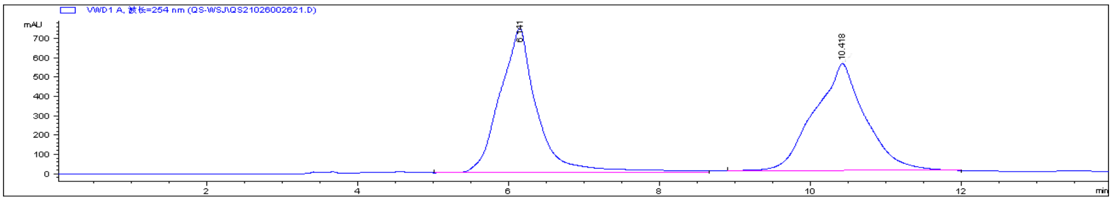

Entry	Retention Time	Area	Height	%Area
1	6.141	25897.5	750.9	49.760
2	10.418	26147.1	554	50.240

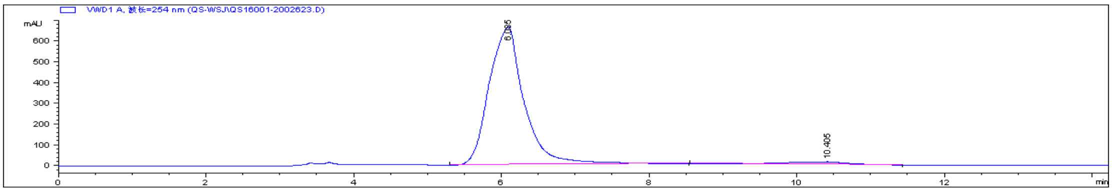

Entry	Retention Time	Area	Height	%Area
1	6.085	21961.2	664.8	96.478
2	10.405	801.6	13.1	3.522

3. Determination of the absolute configuration by *X*-ray crystallography

Absolute configurations of **3** and the derivative **4** are determined by X-ray structure analysis of the product **3r** (CCDC 1495751).

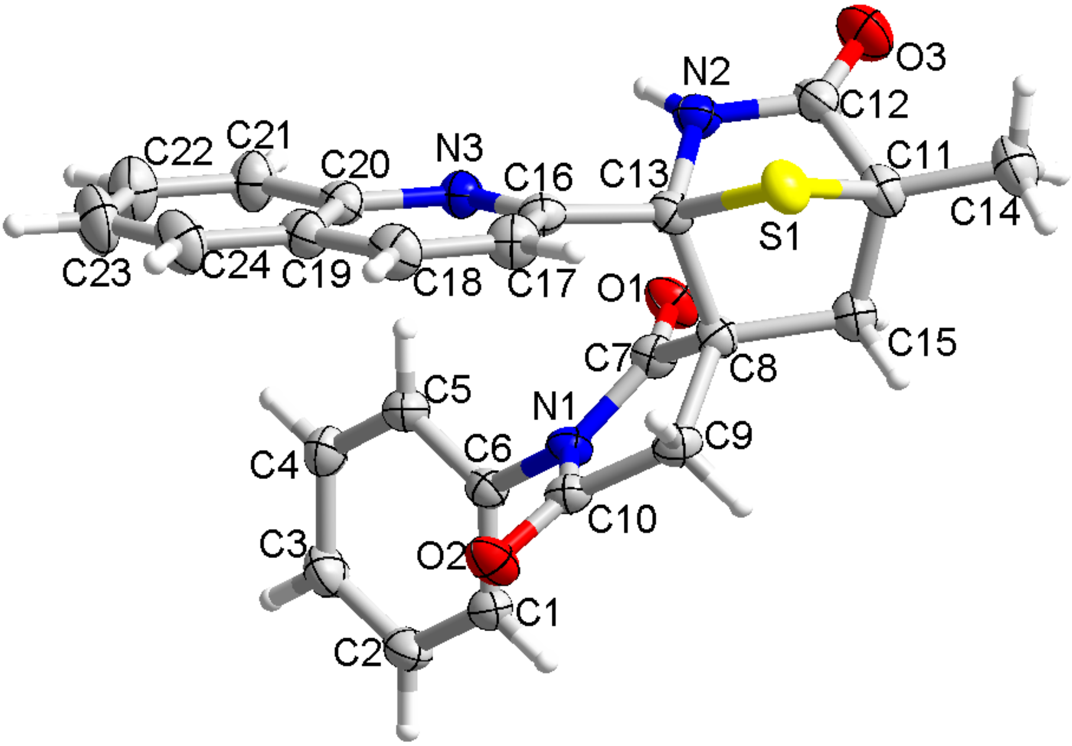

Displacement ellipsoids are drawn at the 30% probability level.

4. Copies of NMR spectra

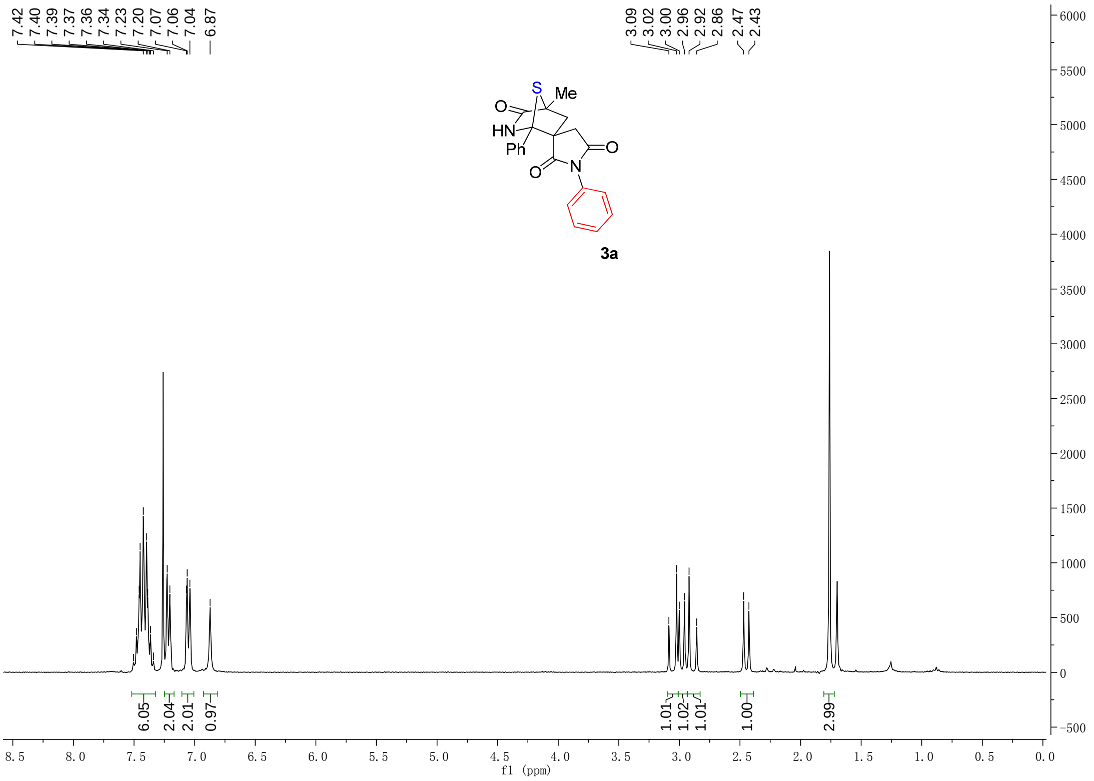

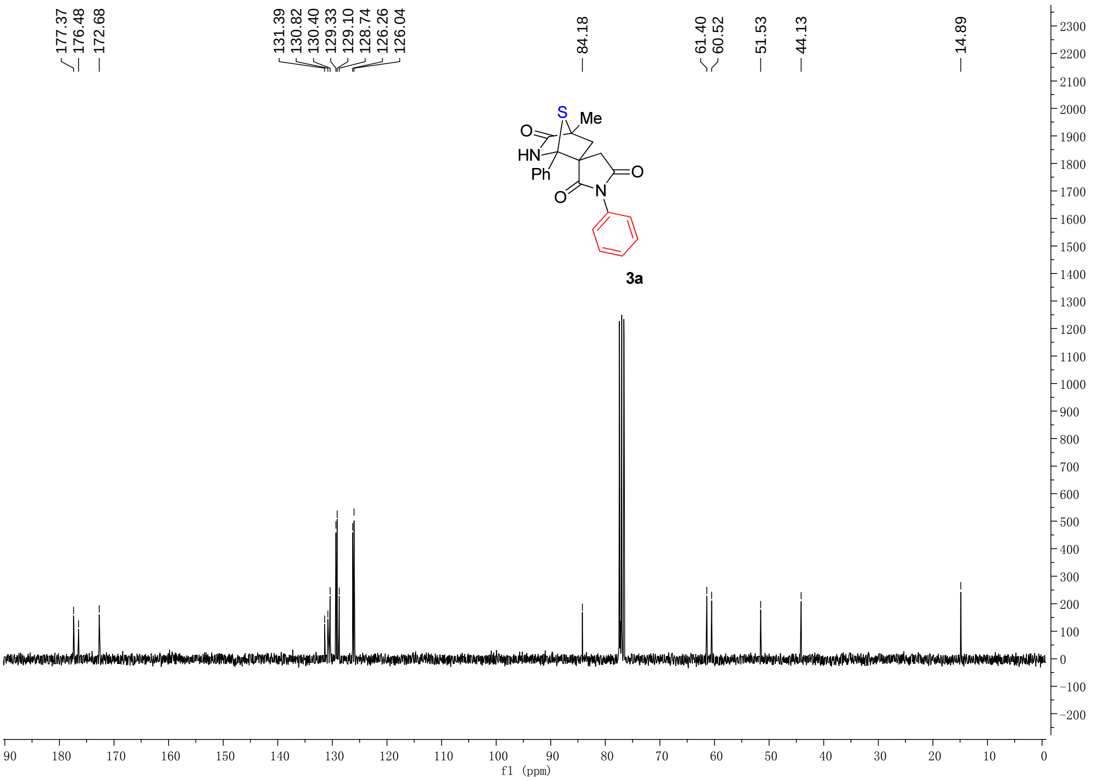

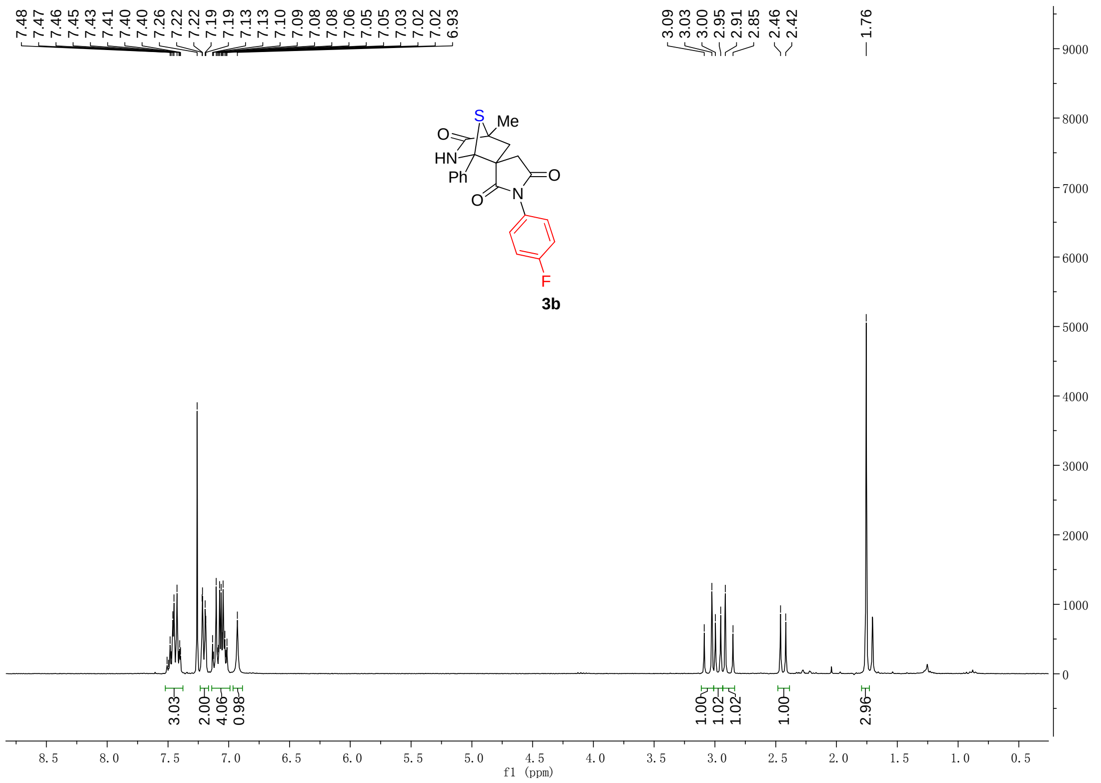
7.48
7.47
7.46
7.45
7.43
7.41
7.40
7.40
7.26
7.22
7.22
7.19
7.19
7.13
7.13
7.10
7.09
7.08
7.08
7.06
7.05
7.05
7.03
7.02
7.02
6.93
3.09
3.03
3.00
2.95
2.91
2.85
2.46
2.42
1.76
3.03
2.00
4.06
0.98
1.00
1.02
1.02
1.00
2.96
S
Me
O
HN
Ph
O
N
O
F
3b
f1 (ppm)

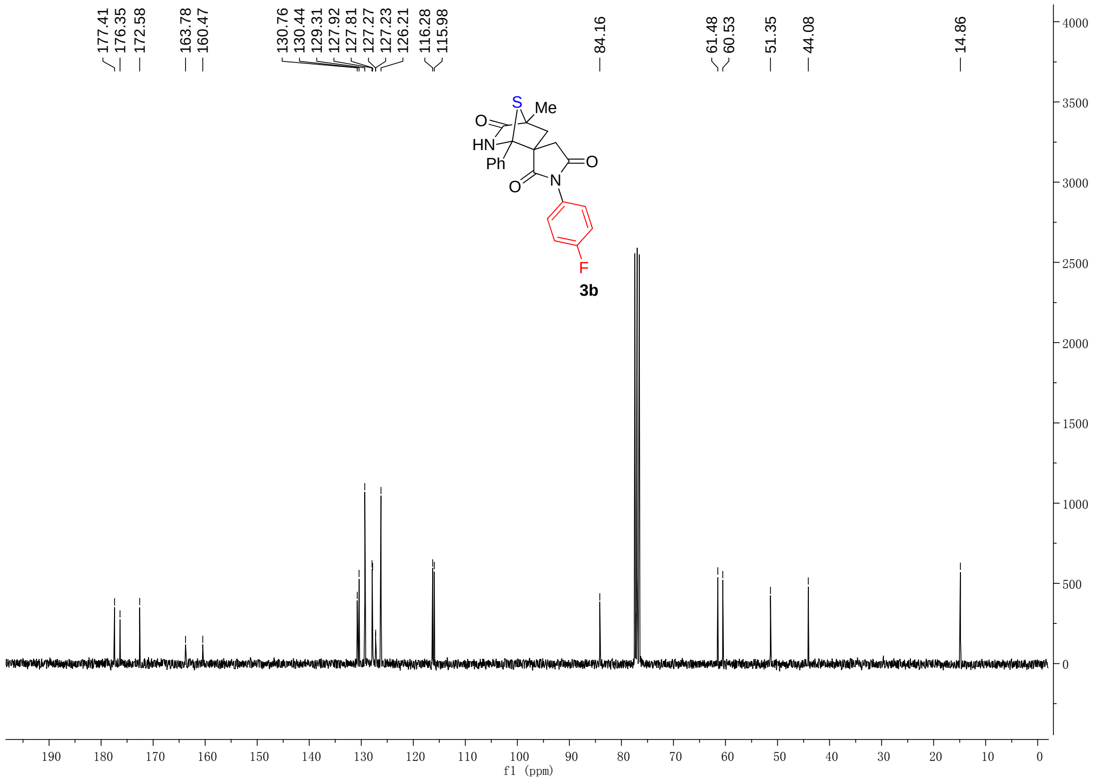
177.41
176.35
172.58
163.78
160.47
130.76
130.44
129.31
127.92
127.81
127.27
127.23
126.21
116.28
115.98
84.16
61.48
60.53
51.35
44.08
14.86
S
Me
O
HN
Ph
O
N
O
F
3b
f1 (ppm)

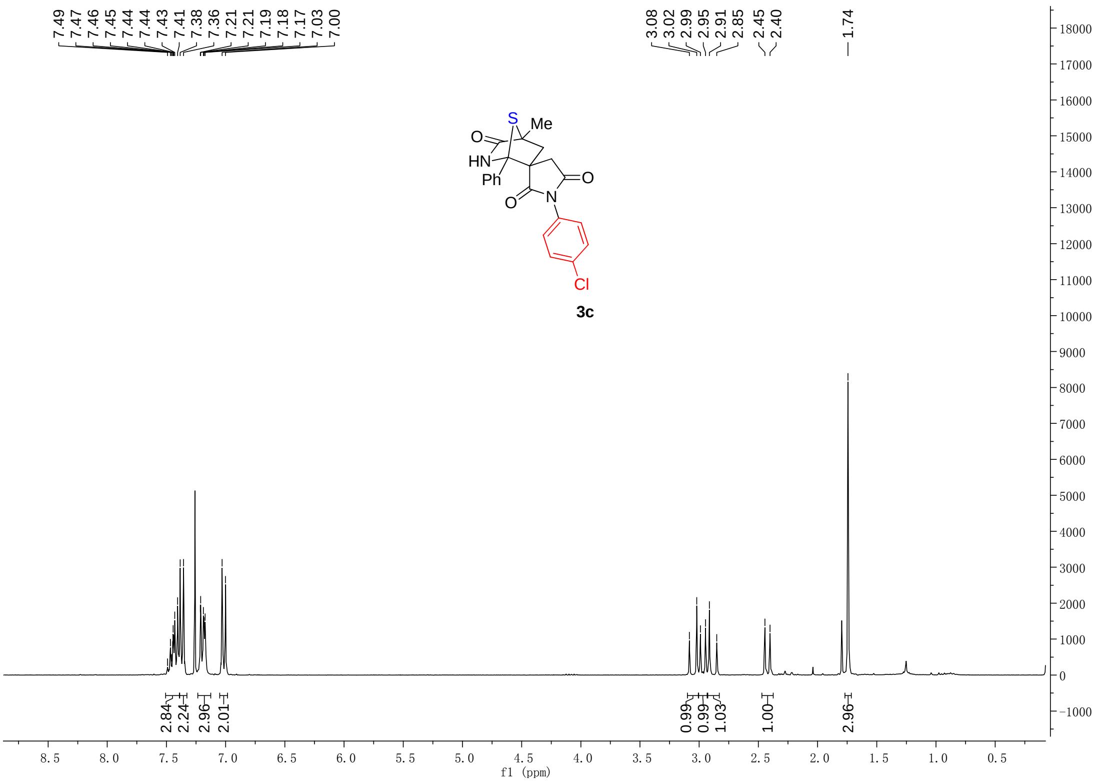
7.49
7.47
7.46
7.45
7.44
7.44
7.43
7.41
7.38
7.36
7.21
7.21
7.19
7.18
7.17
7.03
7.00
3.08
3.02
2.99
2.95
2.91
2.85
2.45
2.40
1.74
S
Me
O
HN
Ph
O
N
O
Cl
3c
2.84
2.24
2.96
2.01
0.99
0.99
1.03
1.00
2.96
f1 (ppm)

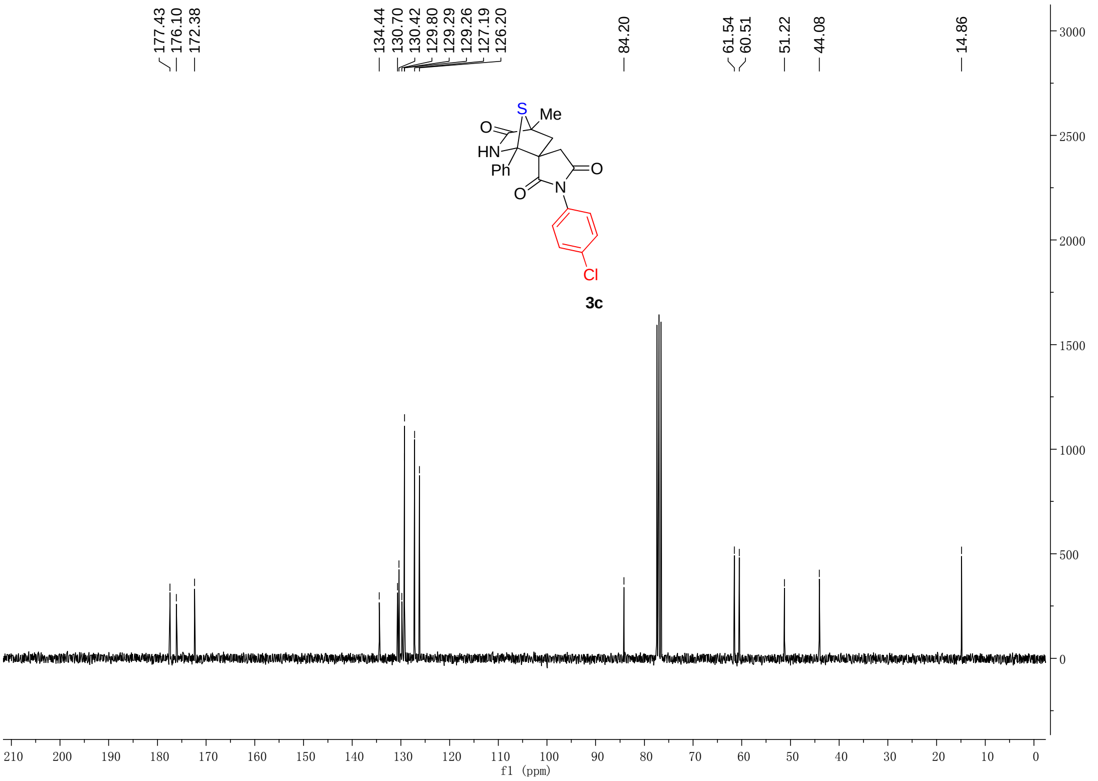
177.43
176.10
172.38
134.44
130.70
130.42
129.80
129.29
129.26
127.19
126.20
84.20
61.54
60.51
51.22
44.08
14.86
S
Me
O
HN
Ph
O
N
O
Cl
3c
f1 (ppm)

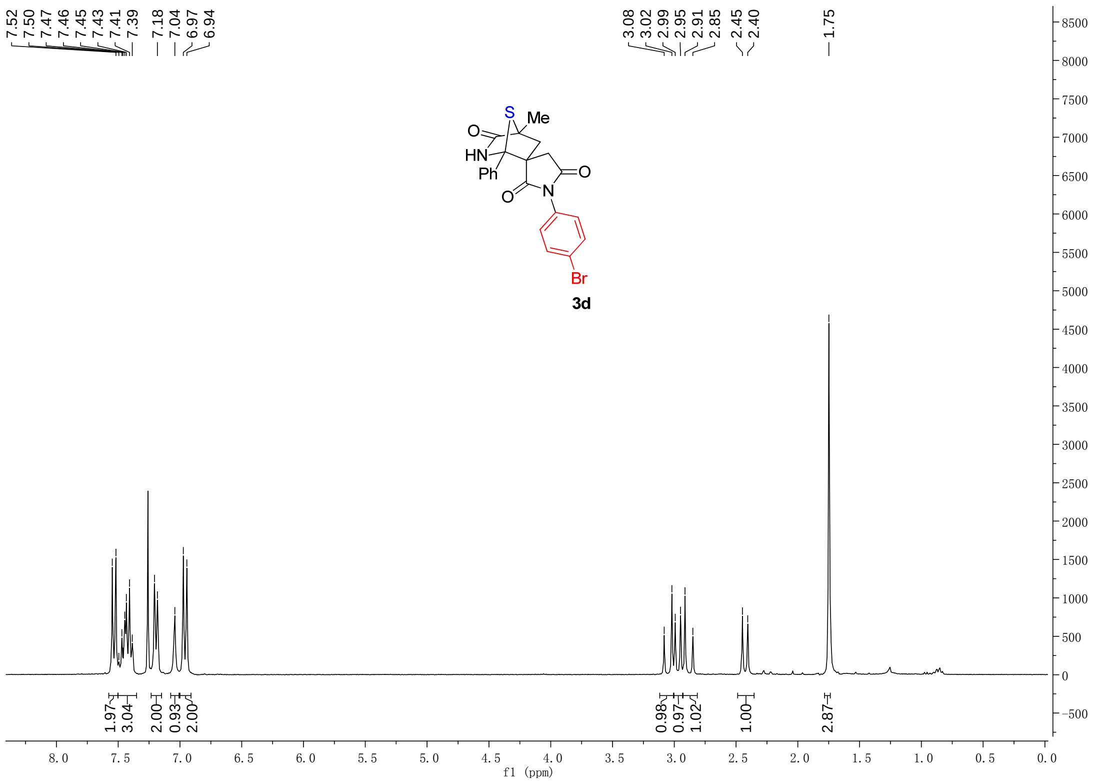
7.52
7.50
7.47
7.46
7.45
7.43
7.41
7.39
7.18
7.04
6.97
6.94
3.08
3.02
2.99
2.95
2.91
2.85
2.45
2.40
1.75
S
Me
O
HN
Ph
O
N
O
Br
3d
1.97
3.04
2.00
0.93
2.00
0.98
0.97
1.02
1.00
2.87
f1 (ppm)

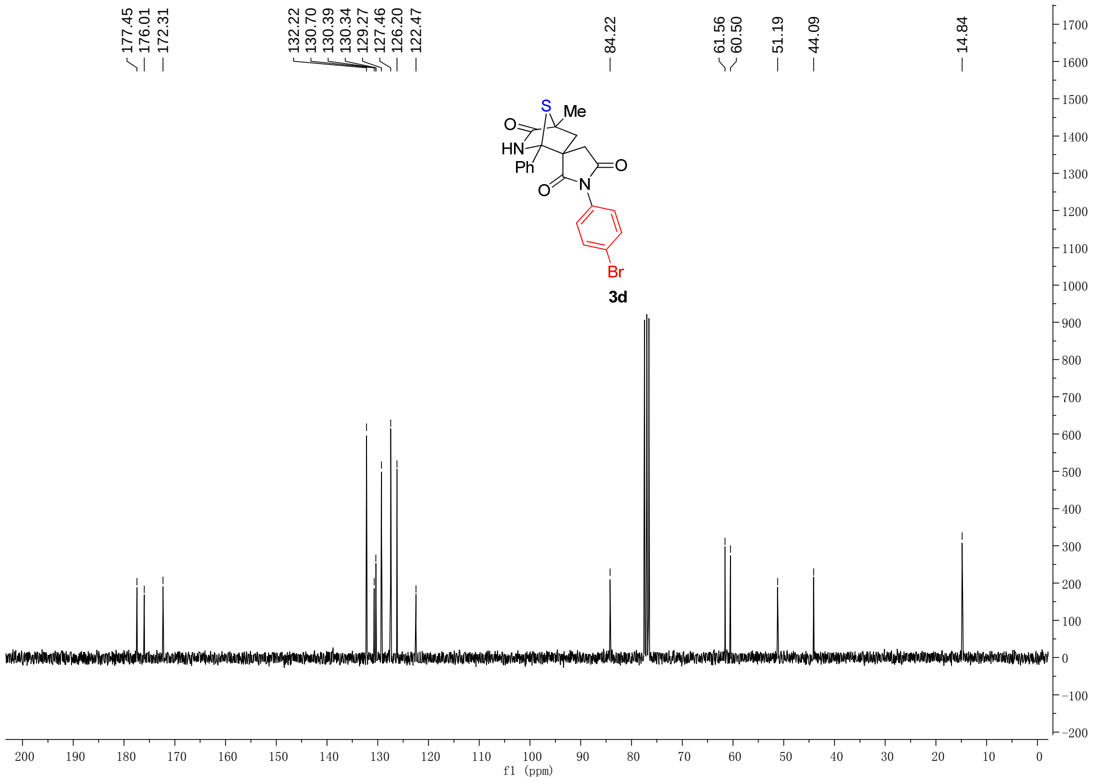
177.45
176.01
172.31
132.22
130.70
130.39
130.34
129.27
127.46
126.20
122.47
84.22
61.56
60.50
51.19
44.09
14.84
S
Me
O
HN
Ph
O
N
O
Br
3d
f1 (ppm)

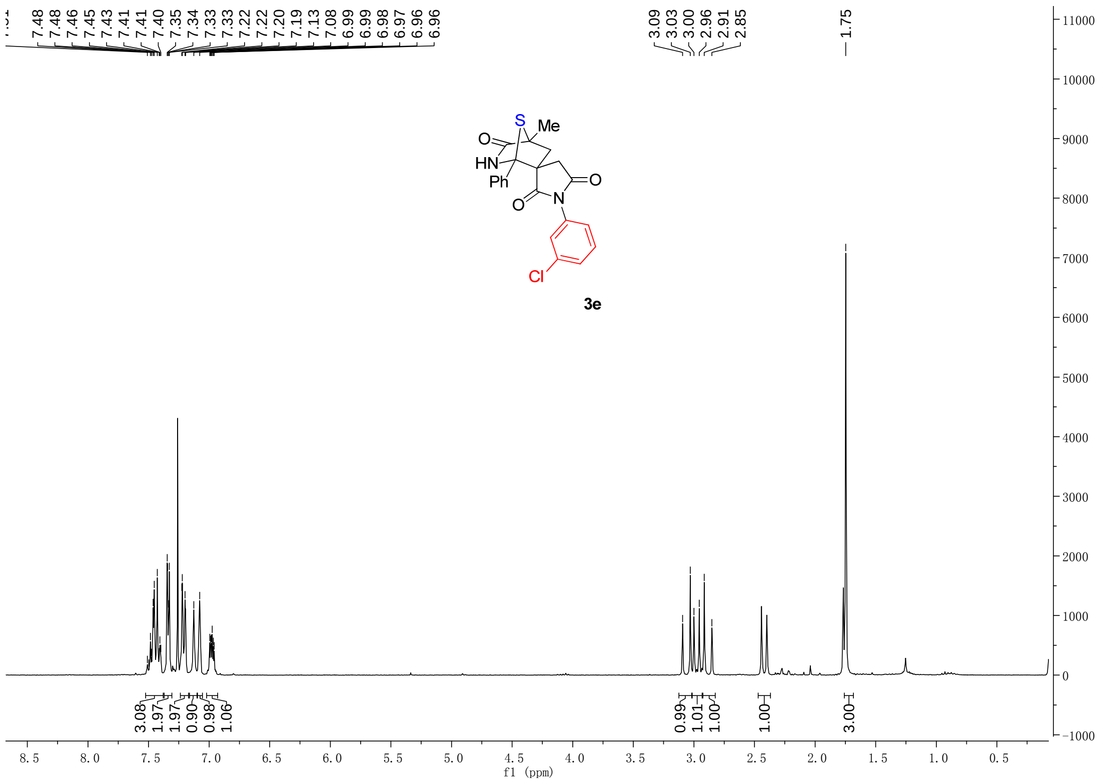
7.48
7.48
7.46
7.45
7.43
7.41
7.40
7.35
7.34
7.33
7.33
7.22
7.22
7.20
7.19
7.13
7.08
6.99
6.99
6.98
6.97
6.96
6.96
3.09
3.03
3.00
2.96
2.91
2.85
1.75
S
Me
O
HN
Ph
O
N
O
Cl
3e
3.08
1.97
1.97
0.90
0.98
1.06
0.99
1.01
1.00
1.00
3.00
f1 (ppm)

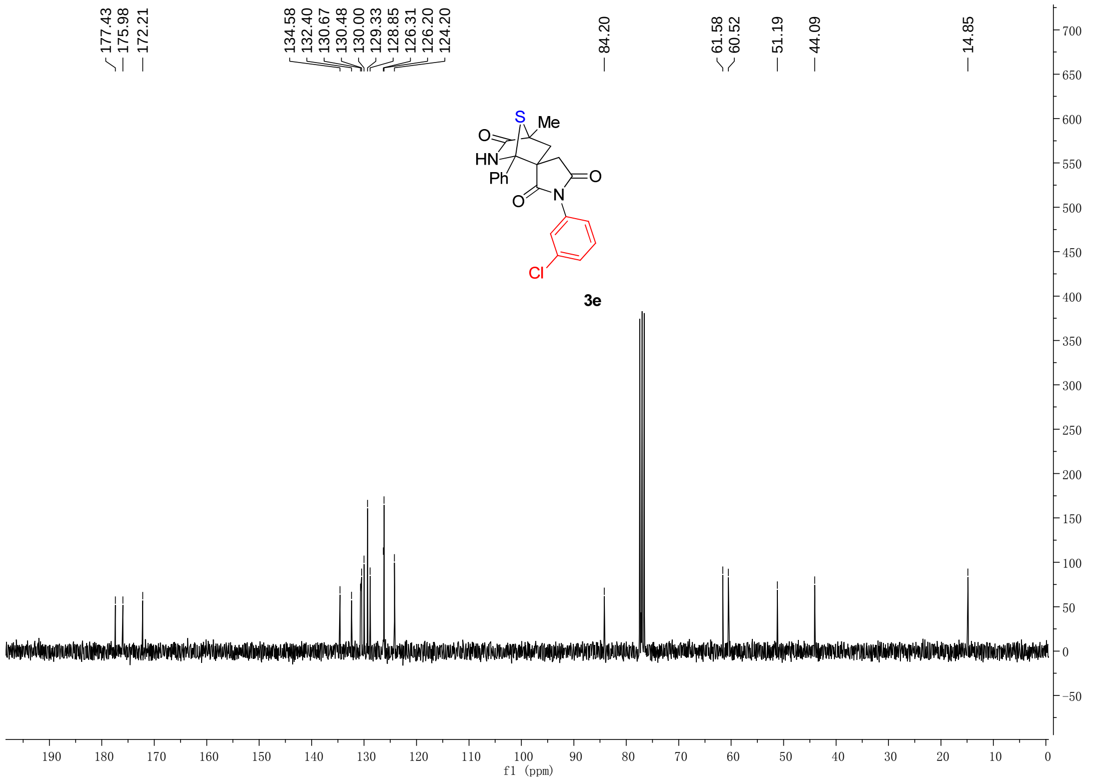
177.43
175.98
172.21
134.58
132.40
130.67
130.48
130.00
129.33
128.85
126.31
126.20
124.20
84.20
61.58
60.52
51.19
44.09
14.85
S
Me
O
HN
Ph
O
N
O
Cl
3e
f1 (ppm)

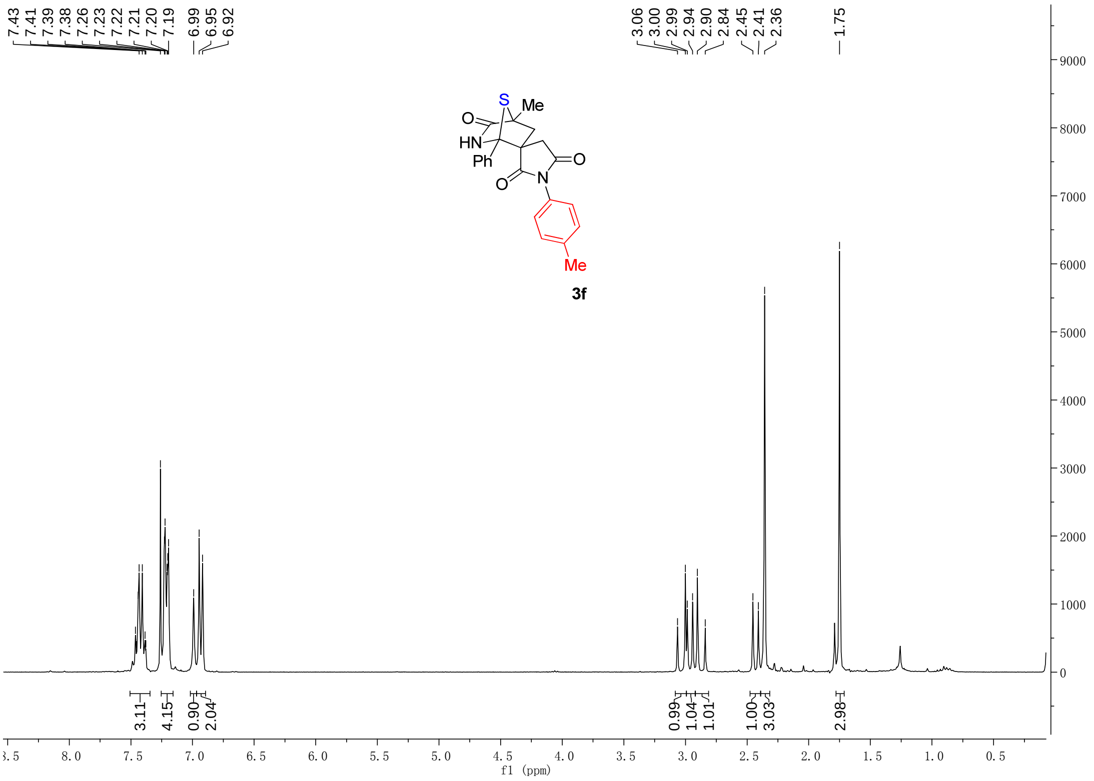
7.43
7.41
7.39
7.38
7.26
7.23
7.22
7.21
7.20
7.19
6.99
6.95
6.92
3.06
3.00
2.99
2.94
2.90
2.84
2.45
2.41
2.36
1.75
S
Me
O
HN
Ph
O
N
O
Me
3f
3.11
4.15
0.90
2.04
0.99
1.04
1.01
1.00
3.03
2.98
f1 (ppm)

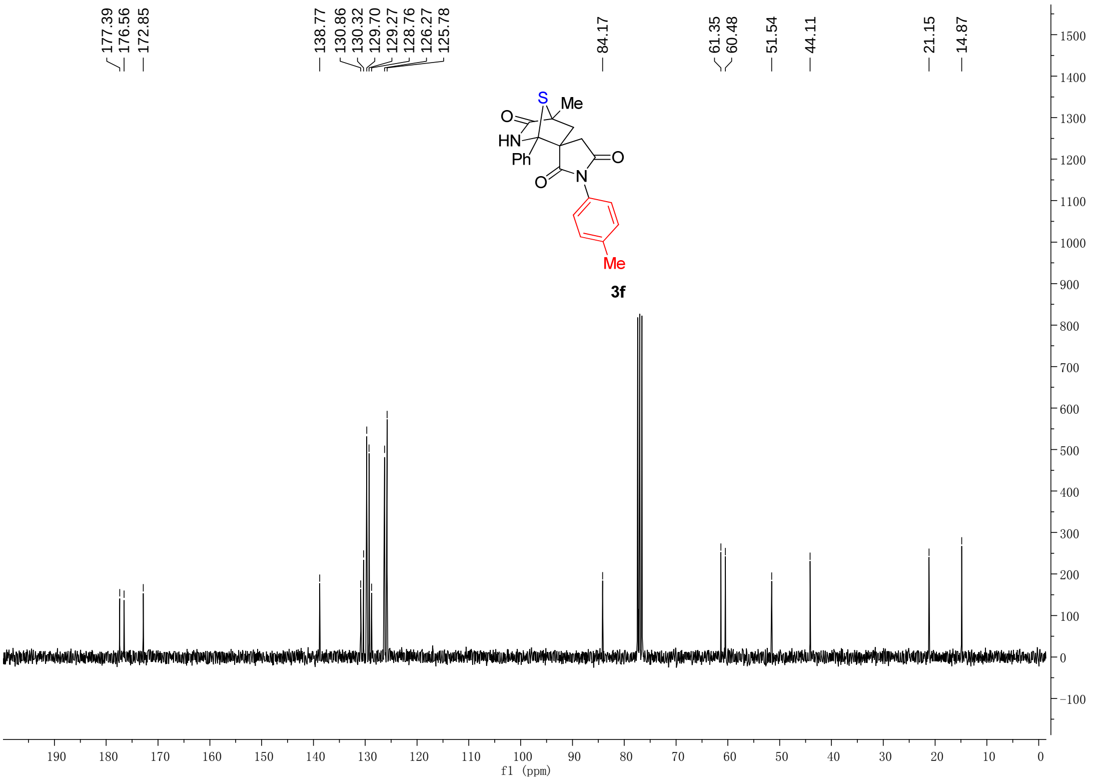
177.39
176.56
172.85
138.77
130.86
130.32
129.70
129.27
128.76
126.27
125.78
84.17
61.35
60.48
51.54
44.11
21.15
14.87
S
Me
O
HN
Ph
O
N
O
Me
3f
f1 (ppm)

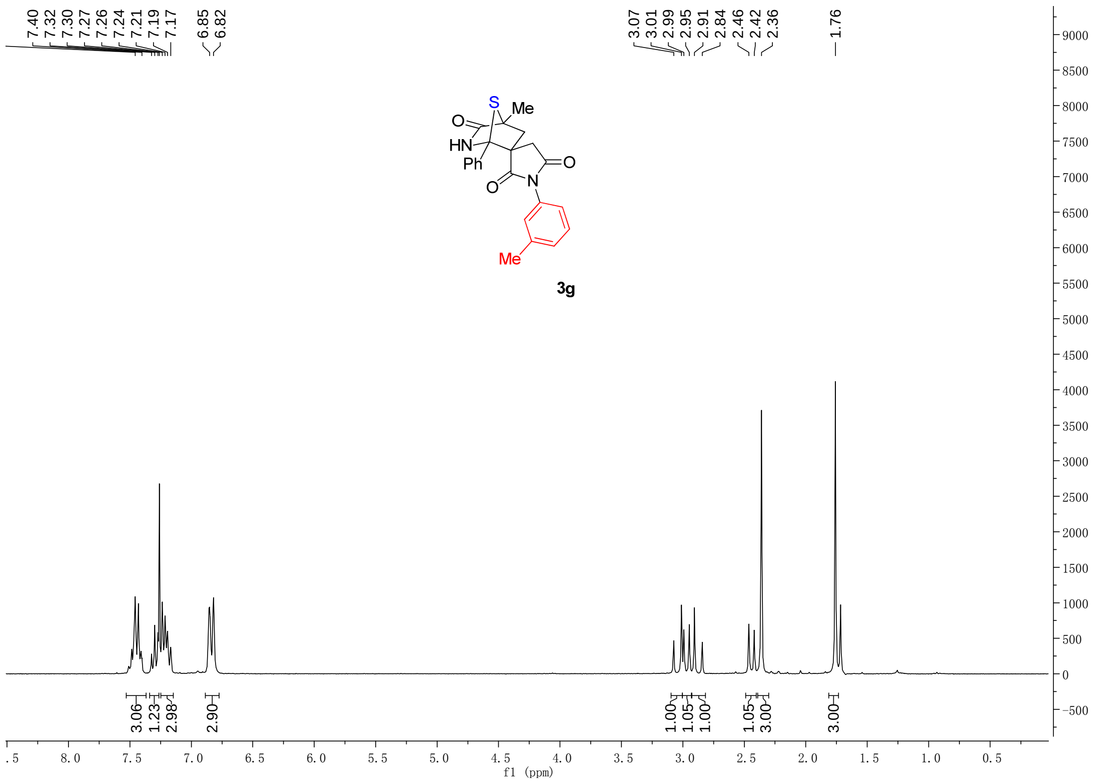

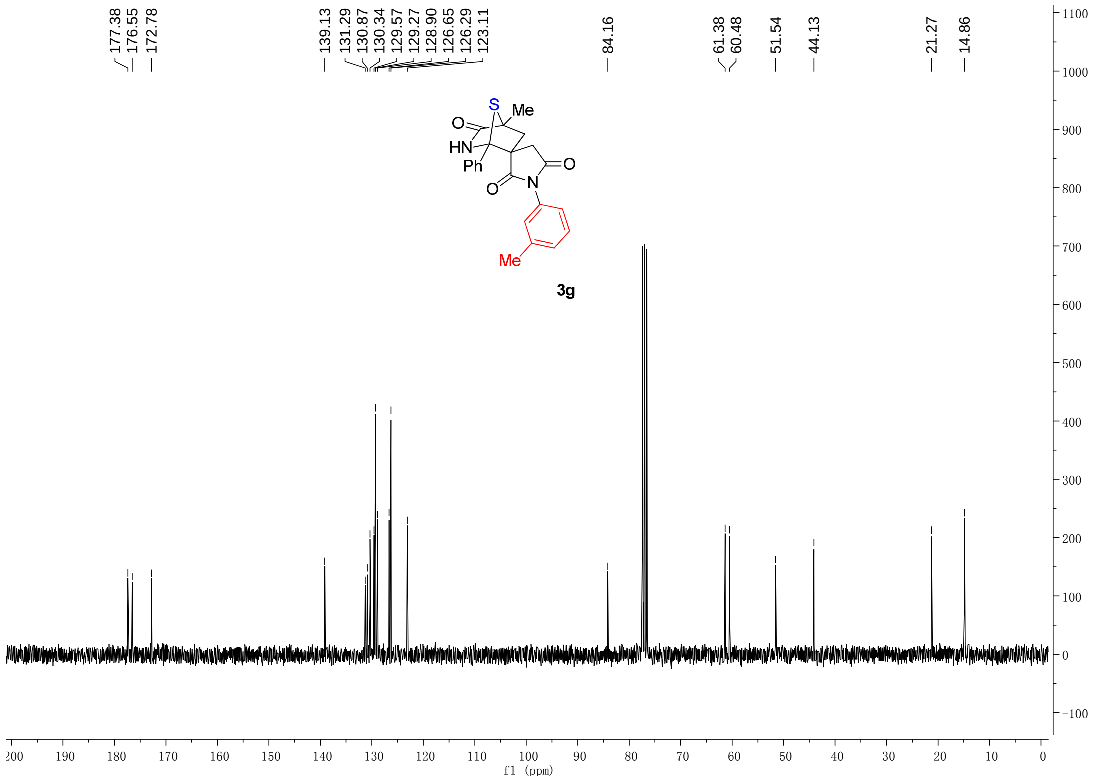

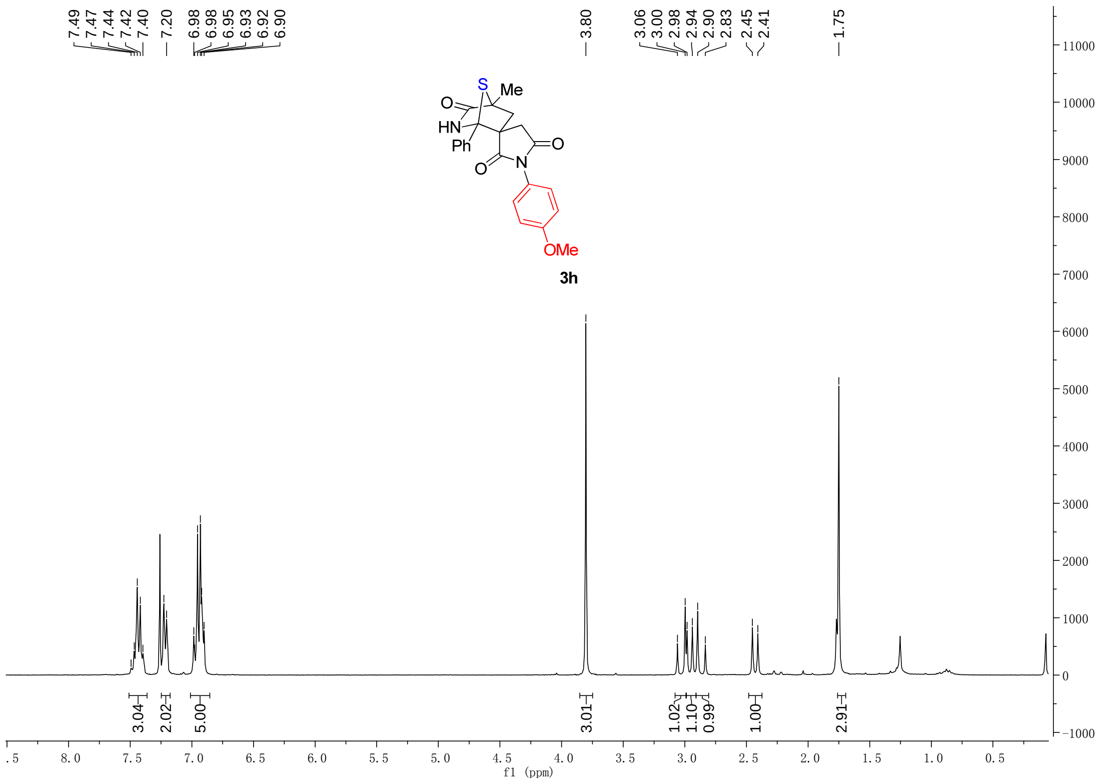
7.49
7.47
7.44
7.42
7.40
7.20
6.98
6.98
6.95
6.93
6.92
6.90
3.80
3.06
3.00
2.98
2.94
2.90
2.83
2.45
2.41
1.75
S
Me
O
HN
Ph
O
N
O
OMe
3h
3.04
2.02
5.00
3.01
1.02
1.10
0.99
1.00
2.91
f1 (ppm)

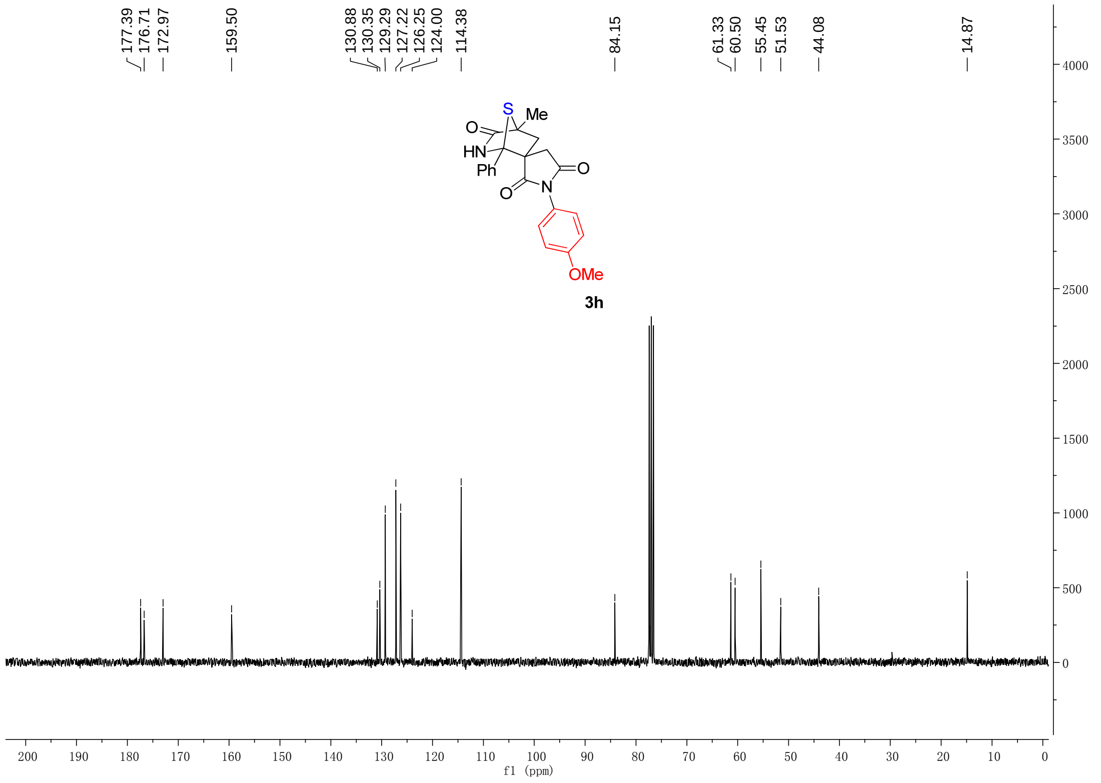
177.39
176.71
172.97
159.50
130.88
130.35
129.29
127.22
126.25
124.00
114.38
84.15
61.33
60.50
55.45
51.53
44.08
14.87
S
Me
O
HN
Ph
O
N
O
OMe
3h
f1 (ppm)

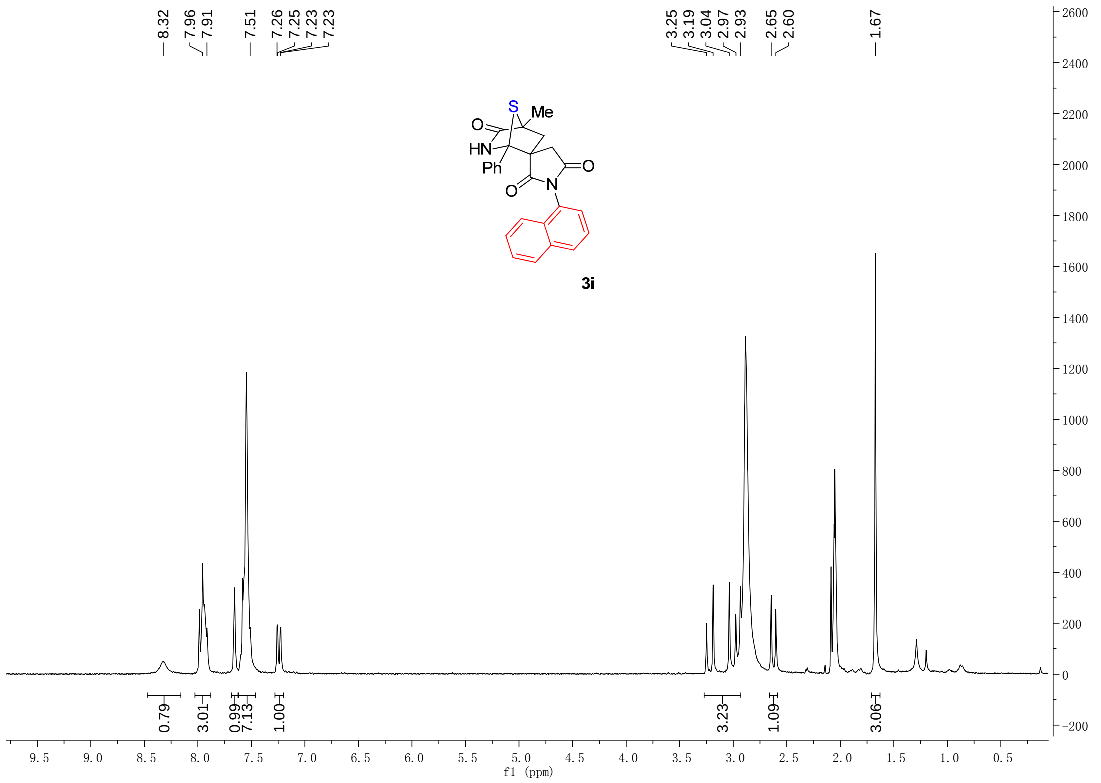
8.32
7.96
7.91
7.51
7.26
7.25
7.23
7.23
3.25
3.19
3.04
2.97
2.93
2.65
2.60
1.67
S
Me
O
HN
Ph
O
N
O
3i
0.79
3.01
0.99
7.13
1.00
3.23
1.09
3.06
f1 (ppm)

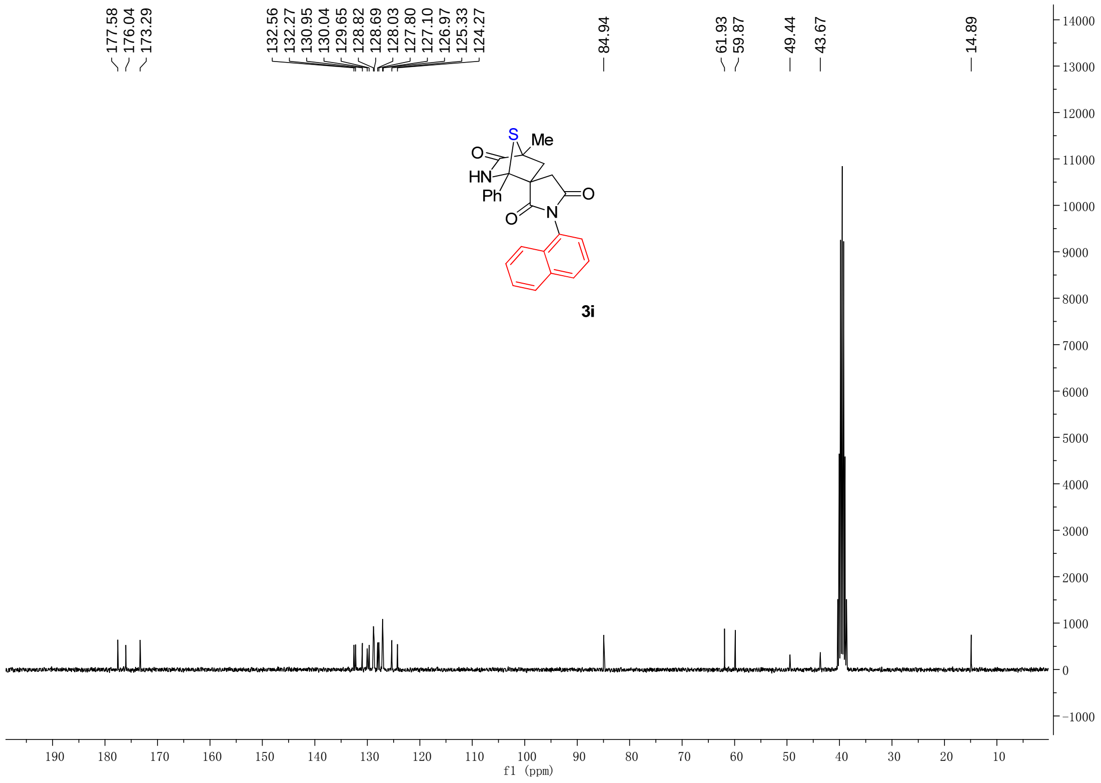
177.58
176.04
173.29
132.56
132.27
130.95
130.04
129.65
128.82
128.69
128.03
127.80
127.10
126.97
125.33
124.27
84.94
61.93
59.87
49.44
43.67
14.89
S
Me
O
HN
Ph
O
N
O
3i
f1 (ppm)

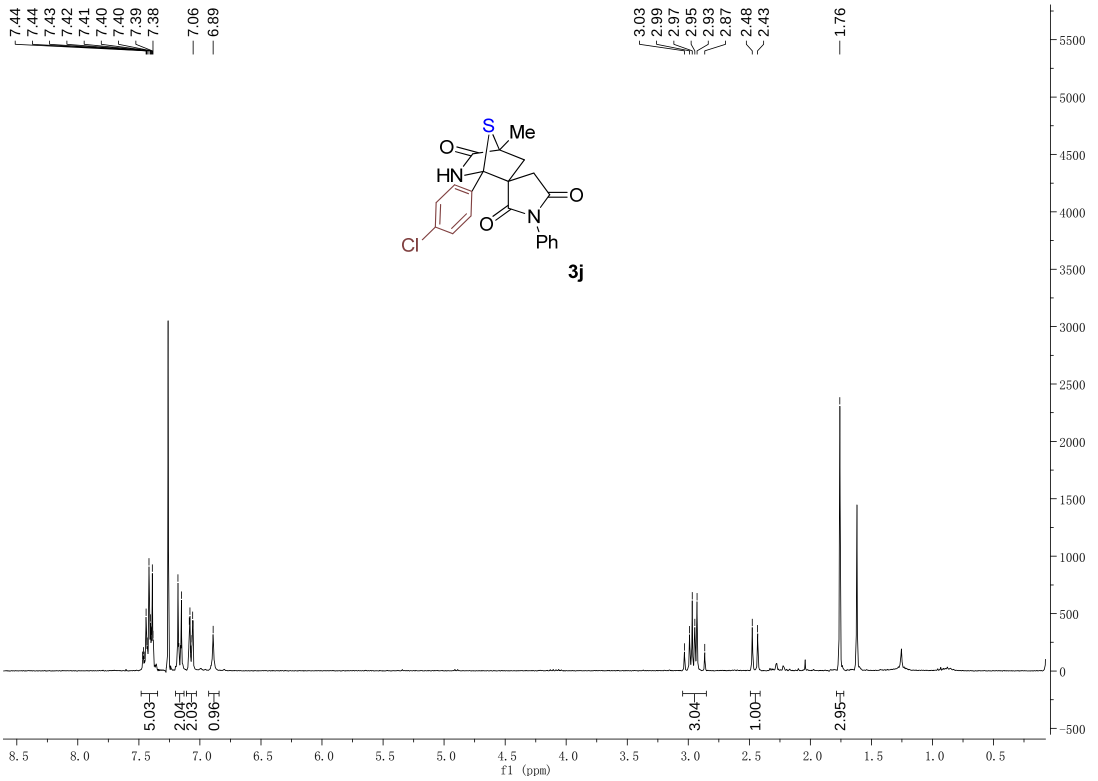
7.44 7.44 7.43 7.42 7.41 7.40 7.40 7.39 7.38 7.06 6.89
3.03 2.99 2.97 2.95 2.93 2.87 2.48 2.43 1.76
S
Me
O
HN
O
N
Cl
O
Ph
3j
5.03
2.04
2.03
0.96
3.04
1.00
2.95
f1 (ppm)

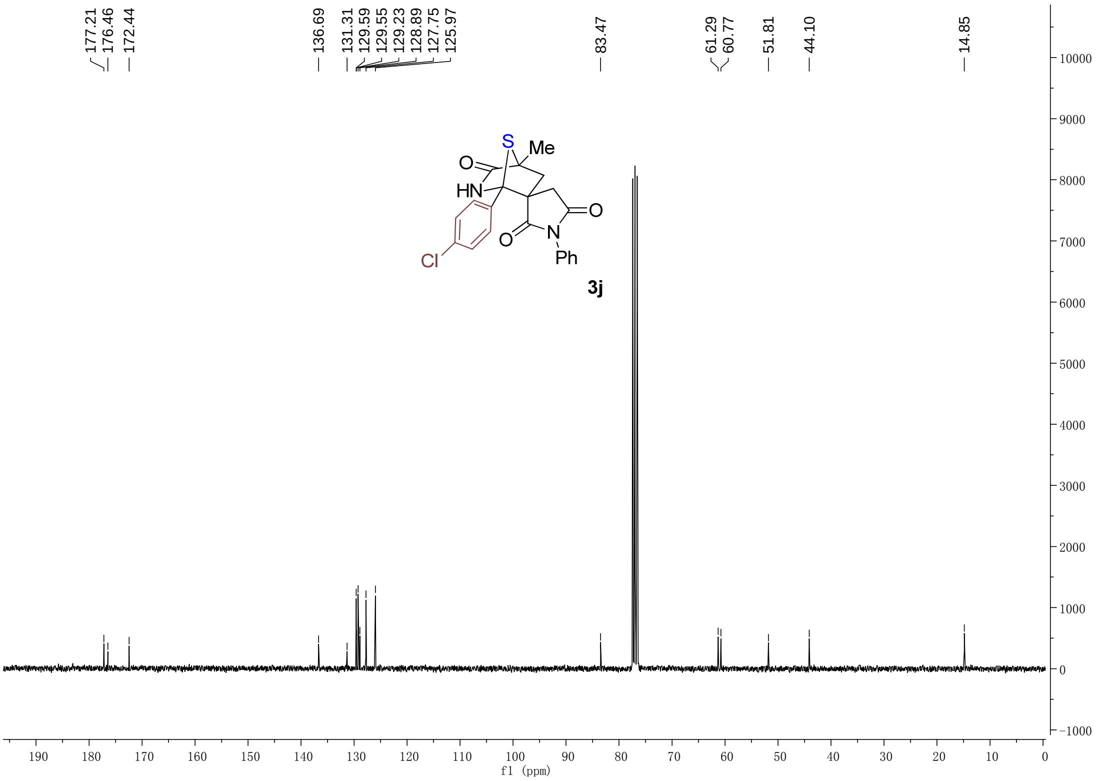
177.21 176.46 172.44
136.69 131.31 129.59 129.55 129.23 128.89 127.75 125.97
83.47
61.29 60.77 51.81 44.10
14.85
S
Me
O
HN
O
N
Cl
O
Ph
3j
f1 (ppm)

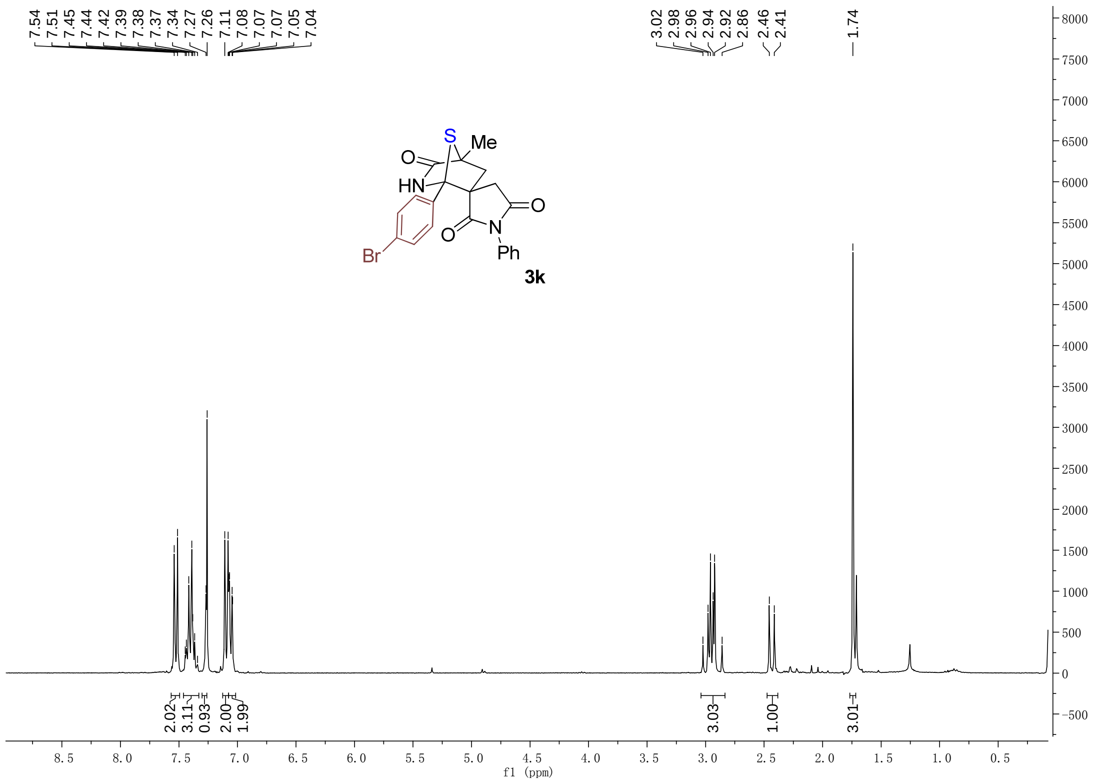
7.54 7.51 7.45 7.44 7.42 7.39 7.38 7.37 7.34 7.27 7.26 7.11 7.08 7.07 7.07 7.05 7.04
3.02 2.98 2.96 2.94 2.92 2.86 2.46 2.41 1.74
S Me O HN O N O Br Ph
3k
2.02 3.11 0.93 2.00 1.99 3.03 1.00 3.01
f1 (ppm)

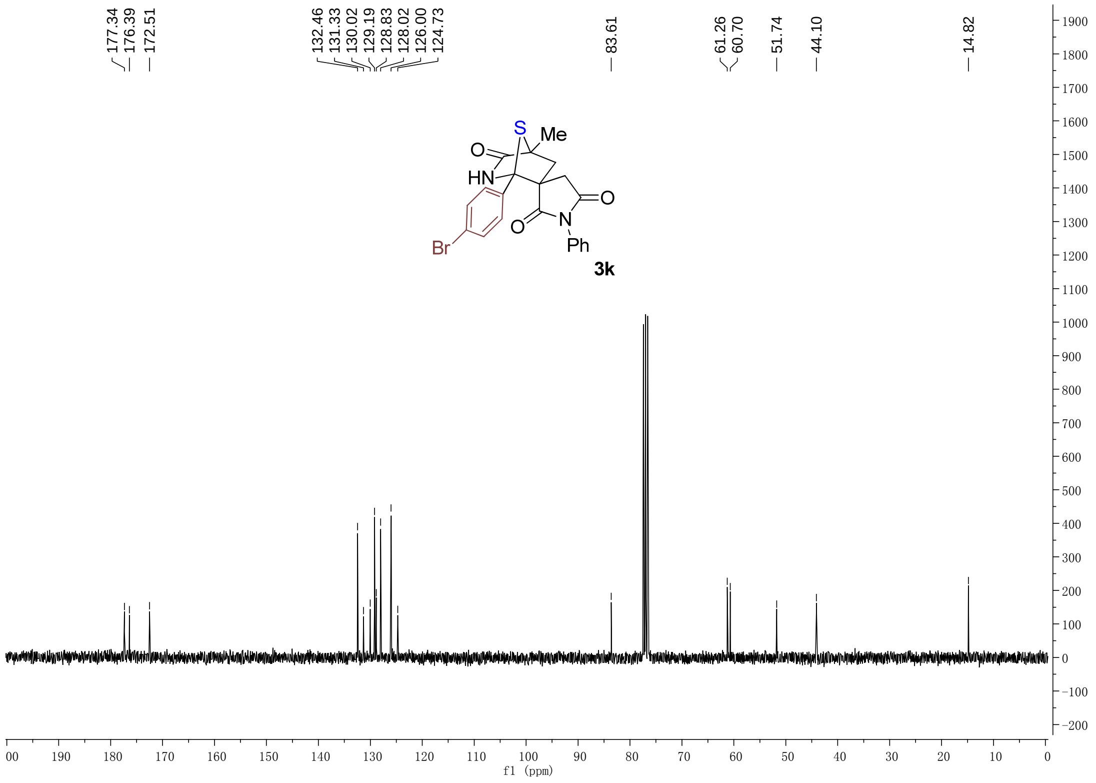
177.34 176.39 172.51
132.46 131.33 130.02 129.19 128.83 128.02 126.00 124.73
83.61
61.26 60.70 51.74 44.10 14.82
S Me O HN O N O Br Ph
3k
f1 (ppm)

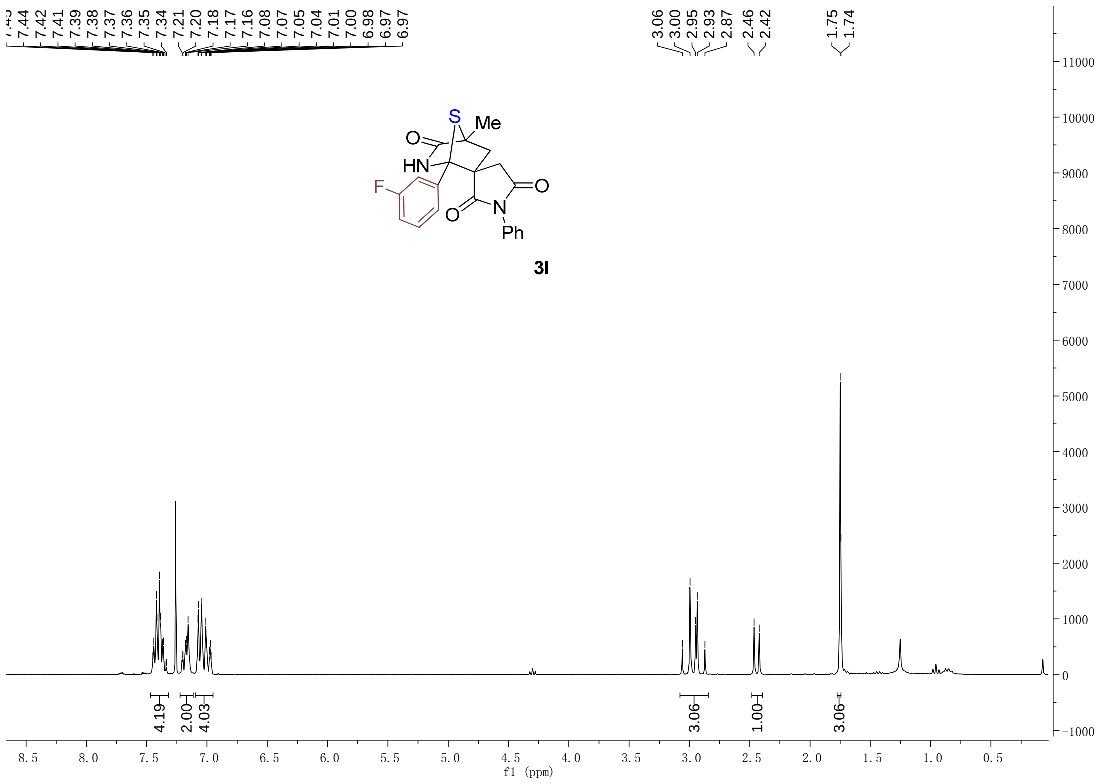
7.44 7.42 7.41 7.39 7.38 7.37 7.36 7.35 7.34 7.21 7.20 7.18 7.17 7.16 7.08 7.07 7.05 7.04 7.01 7.00 6.98 6.97 6.97
3.06 3.00 2.95 2.93 2.87 2.46 2.42
1.75 1.74
4.19 2.00 4.03 3.06 1.00 3.06
3l
f1 (ppm)

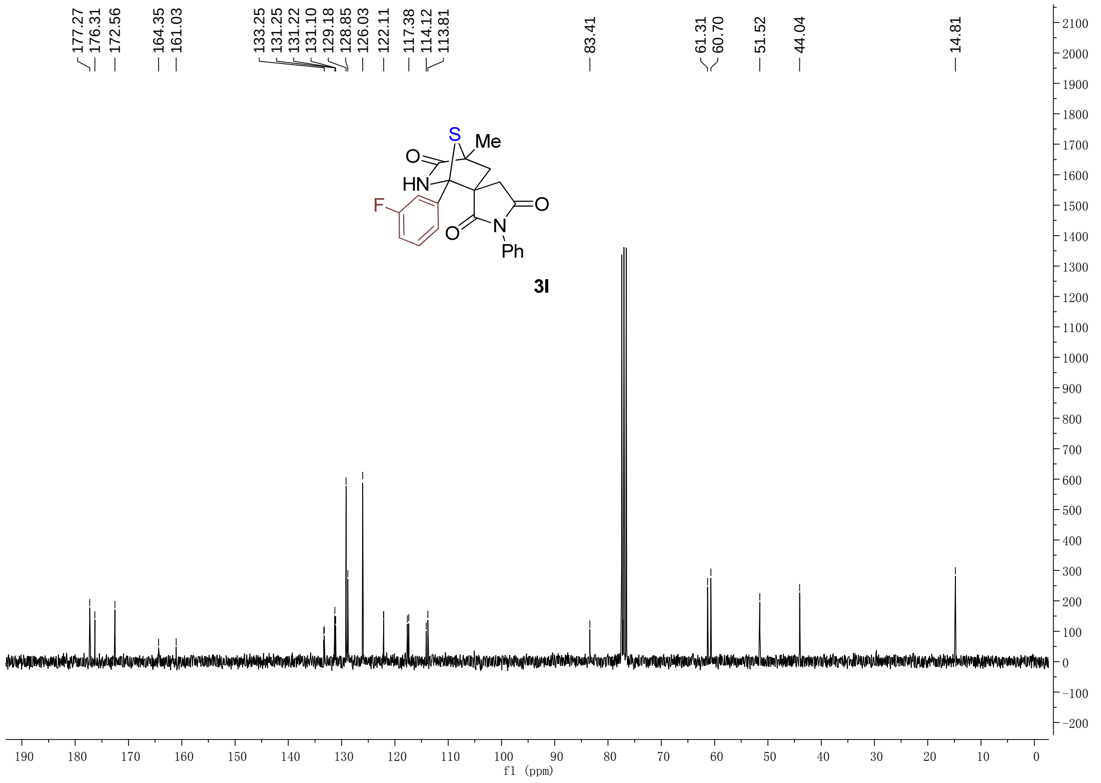
177.27 176.31 172.56 164.35 161.03
133.25 131.25 131.22 131.10 129.18 128.85 126.03 122.11 117.38 114.12 113.81
83.41
61.31 60.70 51.52 44.04
14.81
3l
f1 (ppm)

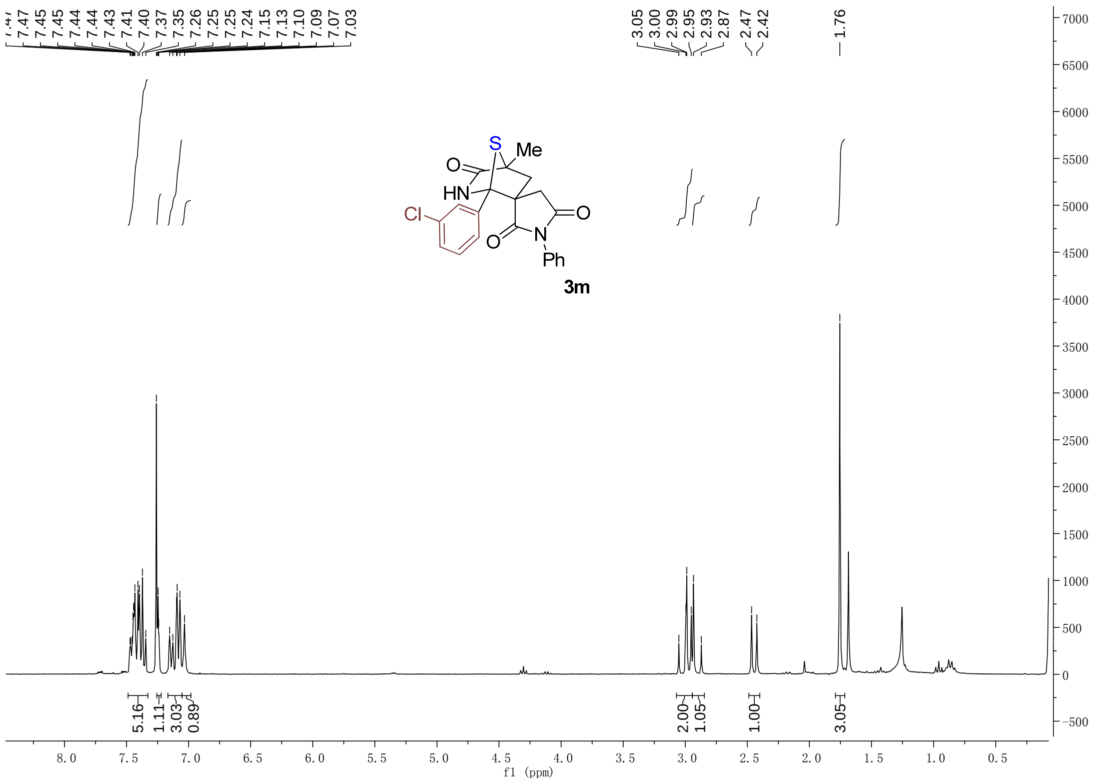
7.47
7.45
7.45
7.44
7.44
7.43
7.41
7.40
7.37
7.35
7.26
7.25
7.25
7.24
7.15
7.13
7.10
7.09
7.07
7.03
3.05
3.00
2.99
2.95
2.93
2.87
2.47
2.42
1.76
S
Me
O
HN
Cl
O
N
O
Ph
3m
5.16
1.11
3.03
0.89
2.00
1.05
1.00
3.05
f1 (ppm)

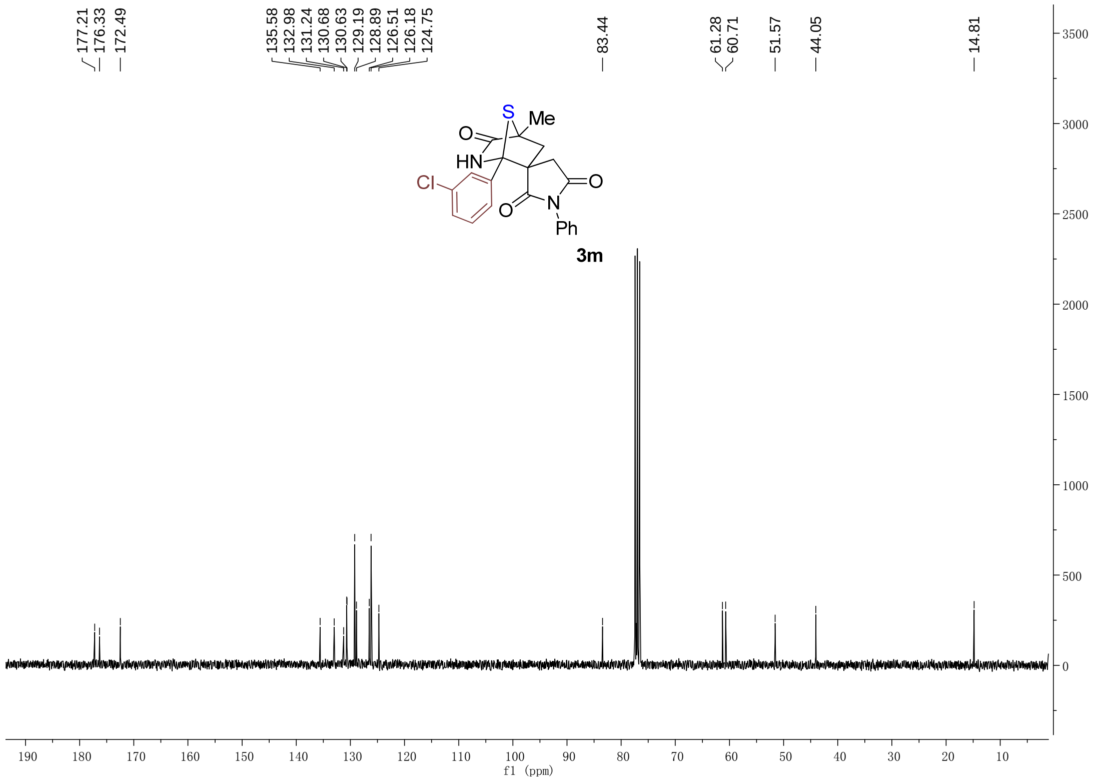
177.21
176.33
172.49
135.58
132.98
131.24
130.68
130.63
129.19
128.89
126.51
126.18
124.75
83.44
61.28
60.71
51.57
44.05
14.81
S
Me
O
HN
Cl
O
N
O
Ph
3m
f1 (ppm)

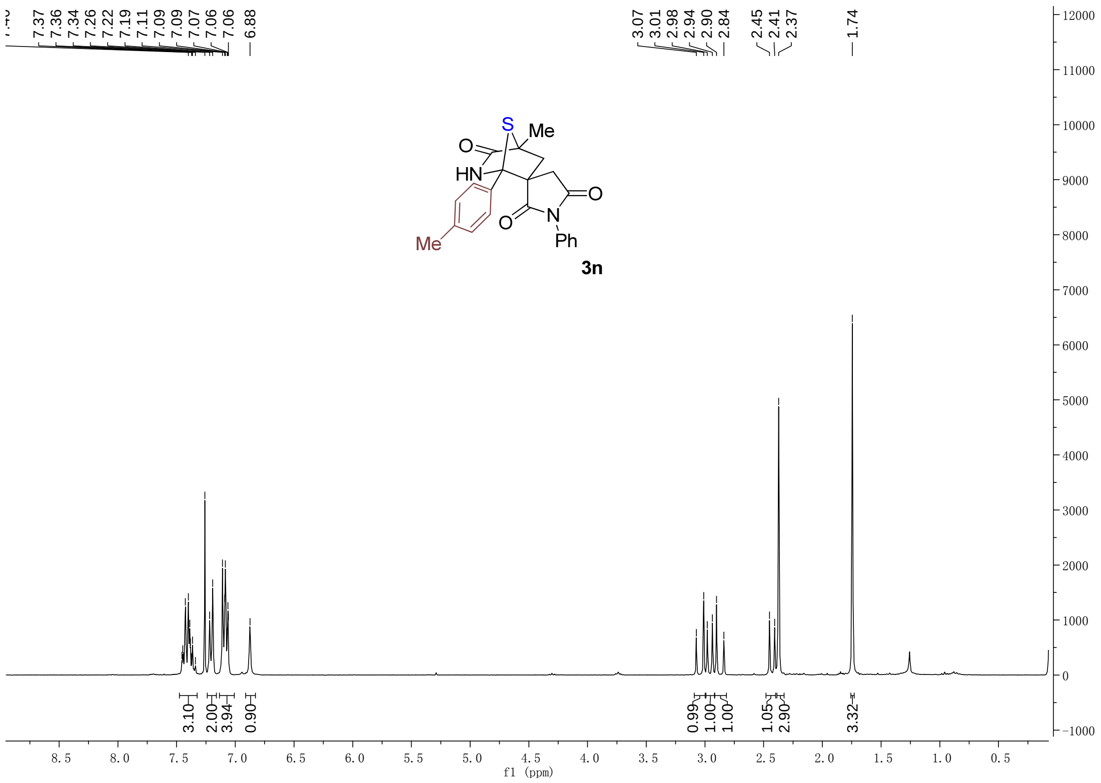
7.37 7.36 7.34 7.26 7.22 7.19 7.11 7.09 7.09 7.07 7.06 7.06 6.88
3.07 3.01 2.98 2.94 2.90 2.84
2.45 2.41 2.37
1.74
S
Me
O
HN
O
O
N
Ph
Me
3n
3.10 2.00 3.94 0.90
0.99 1.00 1.00
1.05 2.90
3.32
f1 (ppm)

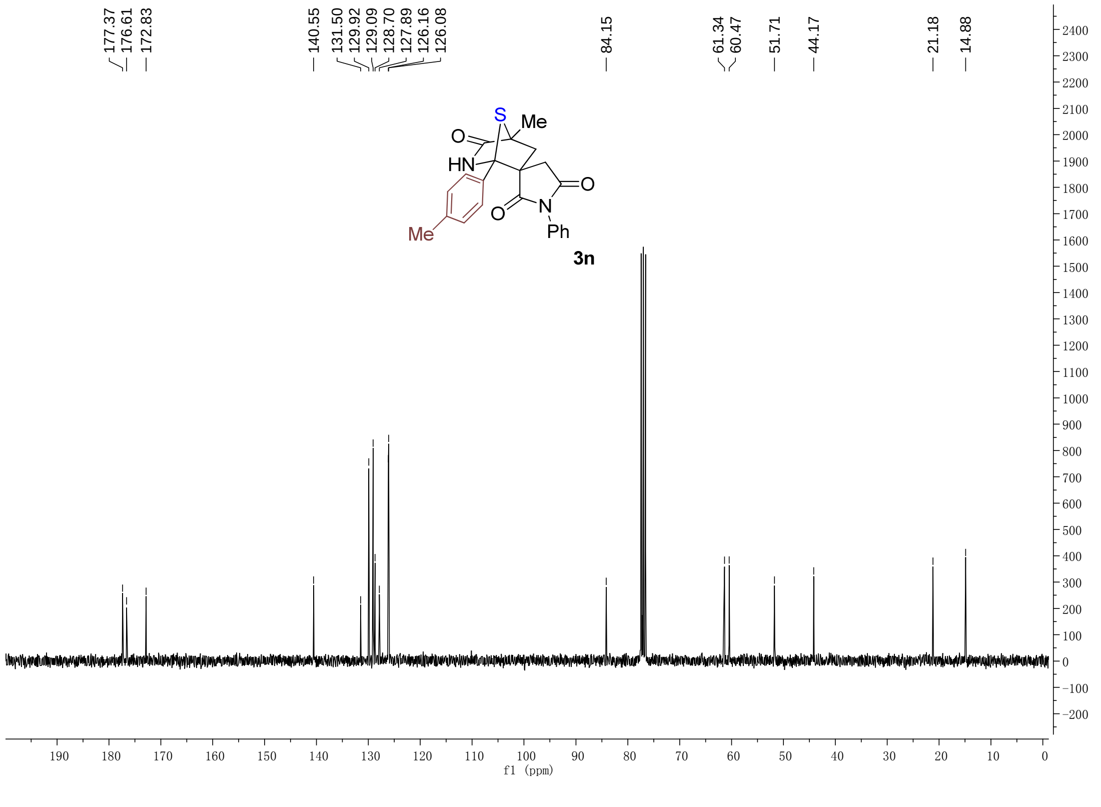
177.37 176.61 172.83
140.55 131.50 129.92 129.09 128.70 127.89 126.16 126.08
84.15
61.34 60.47
51.71
44.17
21.18
14.88
S
Me
O
HN
O
O
N
Ph
Me
3n
f1 (ppm)

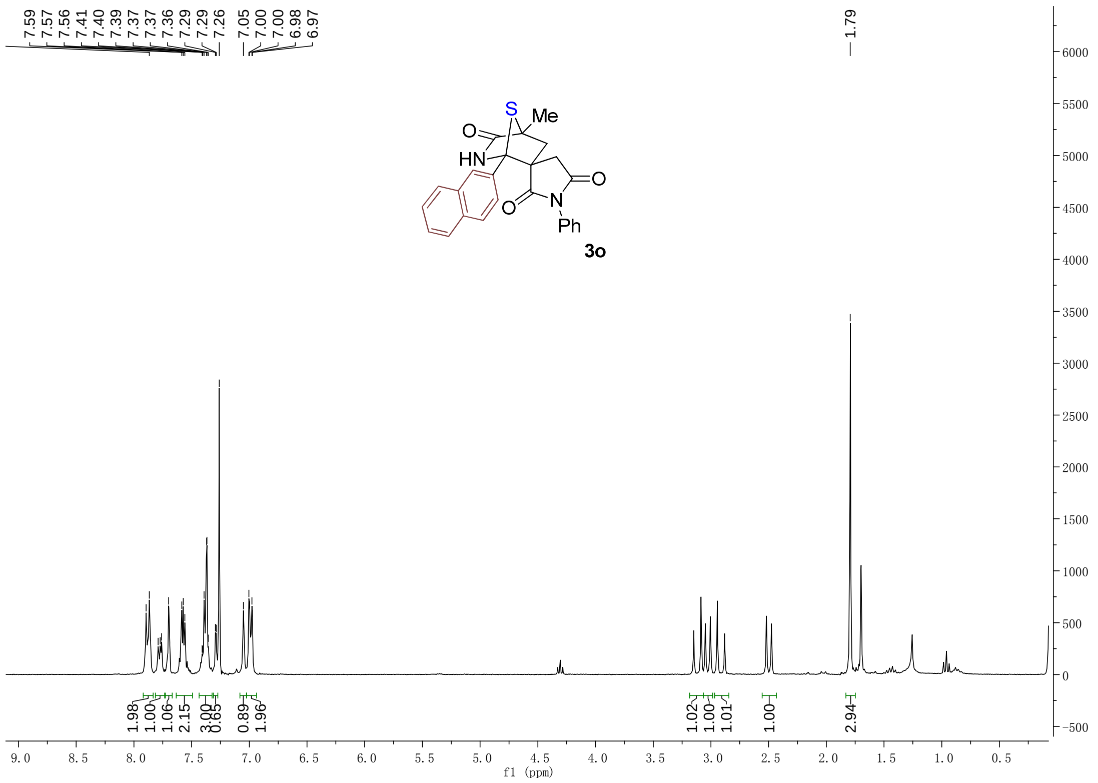
7.59 7.57 7.56 7.41 7.40 7.39 7.37 7.37 7.36 7.29 7.29 7.26 7.05 7.00 7.00 6.98 6.97
1.79
S
Me
O
HN
N
O
O
Ph
3o
1.98 1.00 1.06 2.15 3.00 0.65 0.89 1.96
1.02 1.00 1.01 1.00 2.94
f1 (ppm)

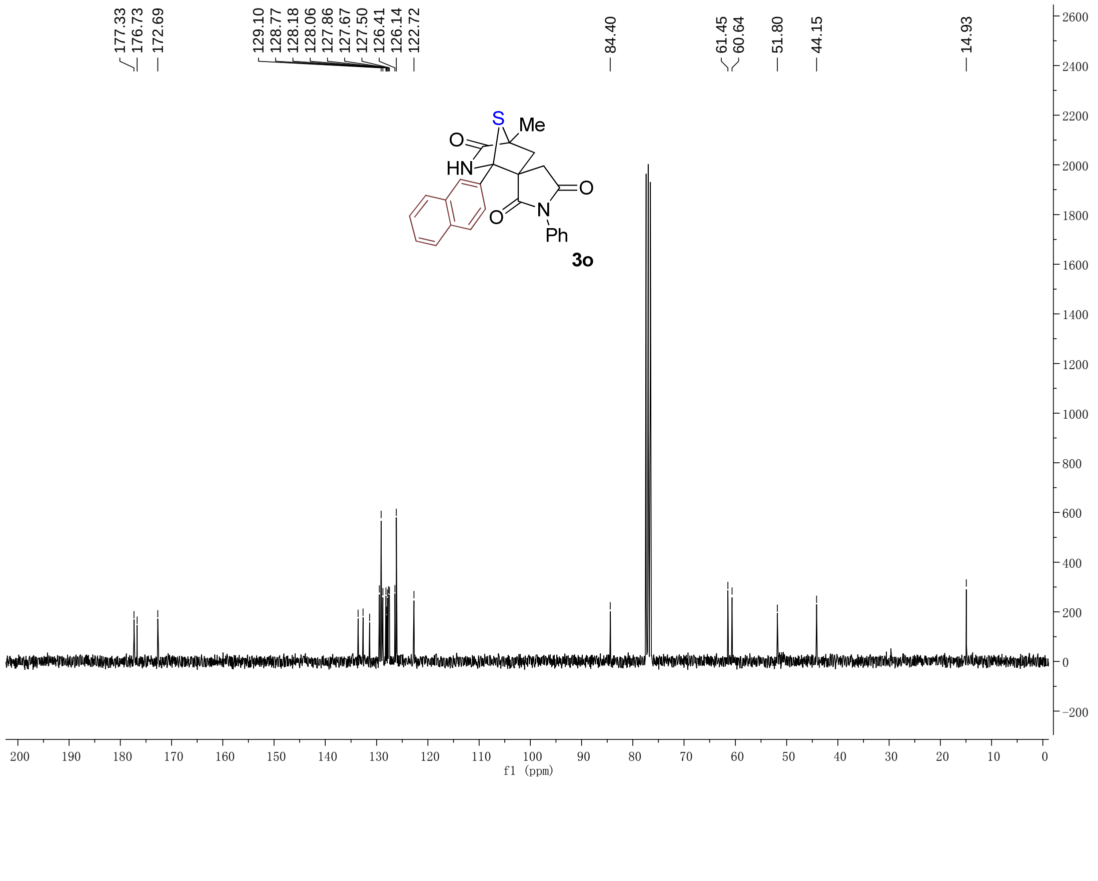
177.33 176.73 172.69
129.10 128.77 128.18 128.06 127.86 127.67 127.50 126.41 126.14 122.72
84.40
61.45 60.64 51.80 44.15
14.93
S
Me
O
HN
N
O
O
Ph
3o
f1 (ppm)

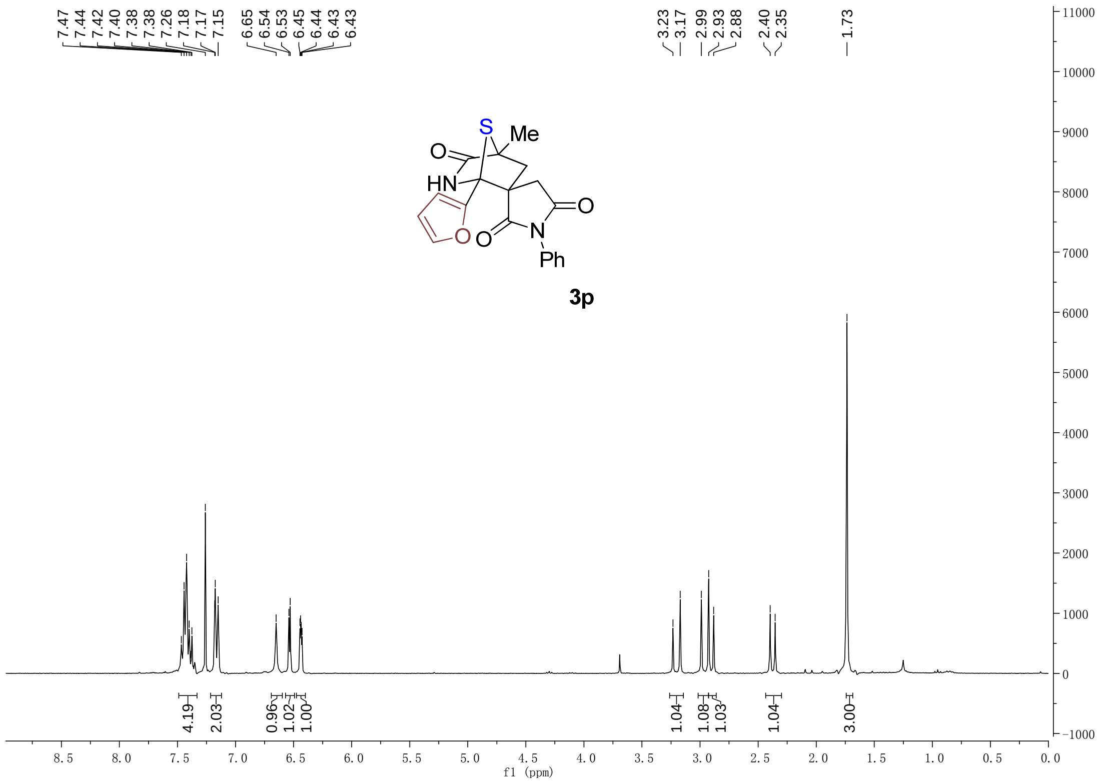
7.47
7.44
7.42
7.40
7.38
7.38
7.26
7.18
7.17
7.15
6.65
6.54
6.53
6.45
6.44
6.43
6.43
3.23
3.17
2.99
2.93
2.88
2.40
2.35
1.73
S
Me
O
HN
O
N
O
O
Ph
3p
4.19
2.03
0.96
1.02
1.00
1.04
1.08
1.03
1.04
3.00
f1 (ppm)

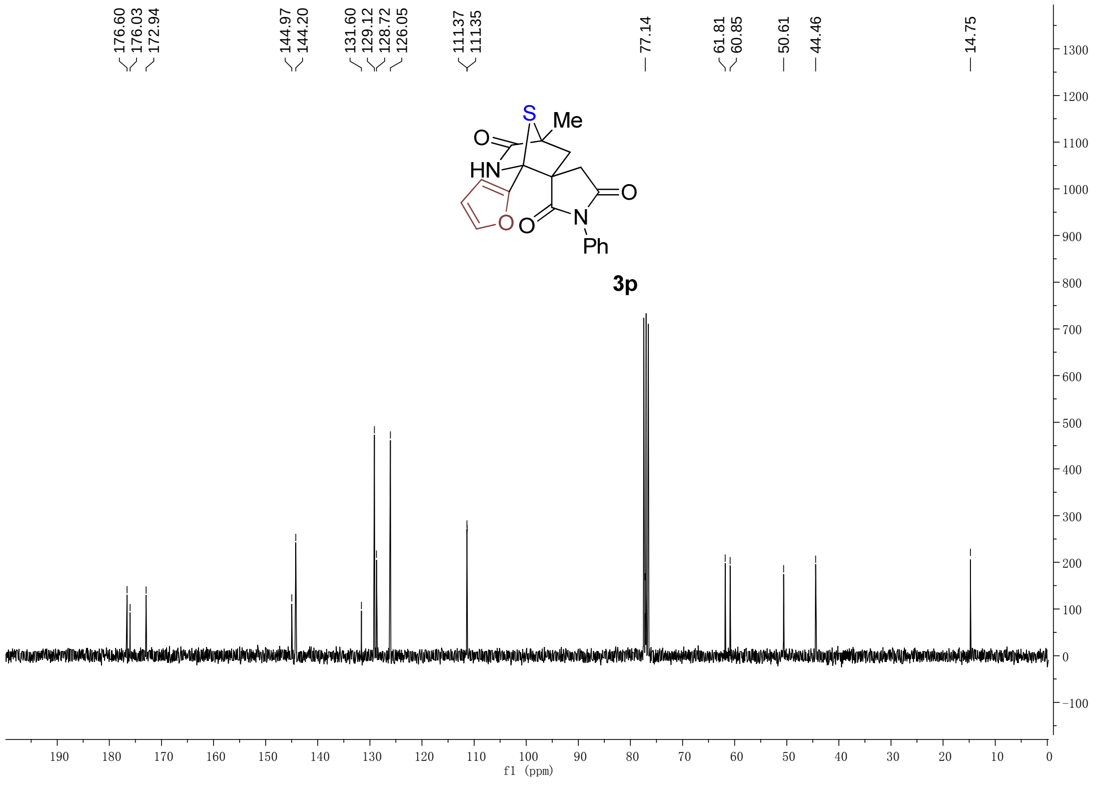
176.60
176.03
172.94
144.97
144.20
131.60
129.12
128.72
126.05
111.37
111.35
77.14
61.81
60.85
50.61
44.46
14.75
S
Me
O
HN
O
N
O
O
Ph
3p
f1 (ppm)

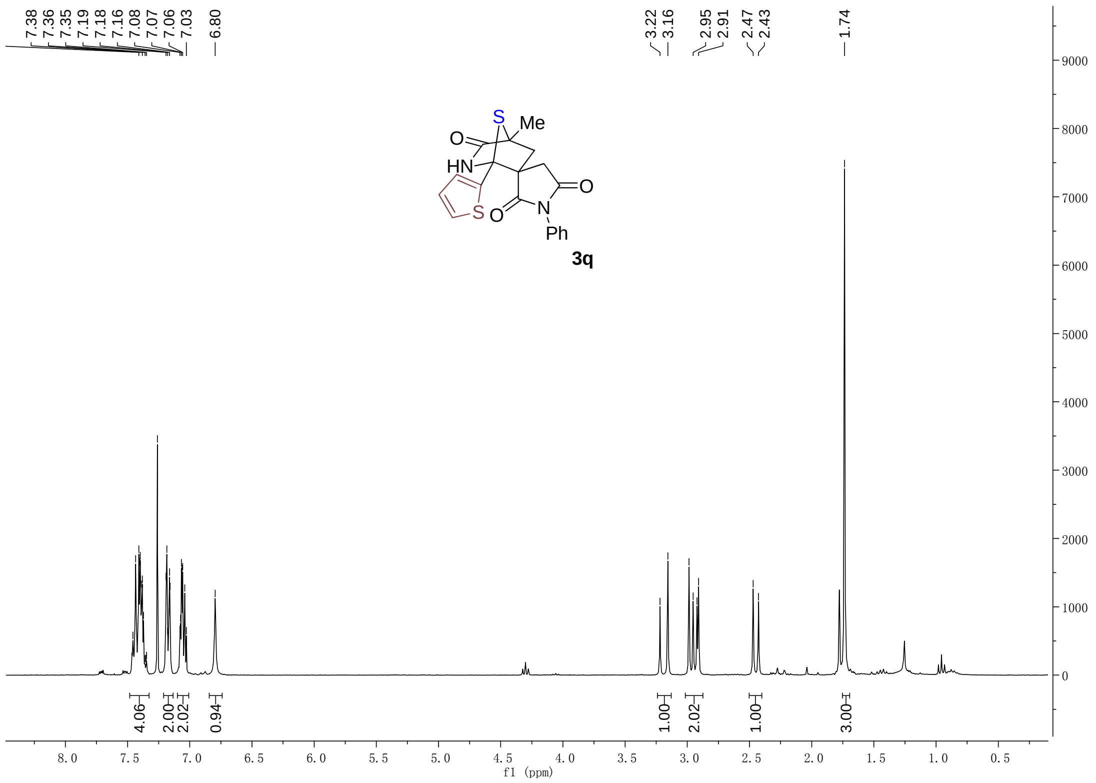
7.38
7.36
7.35
7.19
7.18
7.16
7.08
7.07
7.06
7.03
6.80
3.22
3.16
2.95
2.91
2.47
2.43
1.74
S
Me
O
HN
S
O
N
O
Ph
3q
4.06
2.00
2.02
0.94
1.00
2.02
1.00
3.00
f1 (ppm)

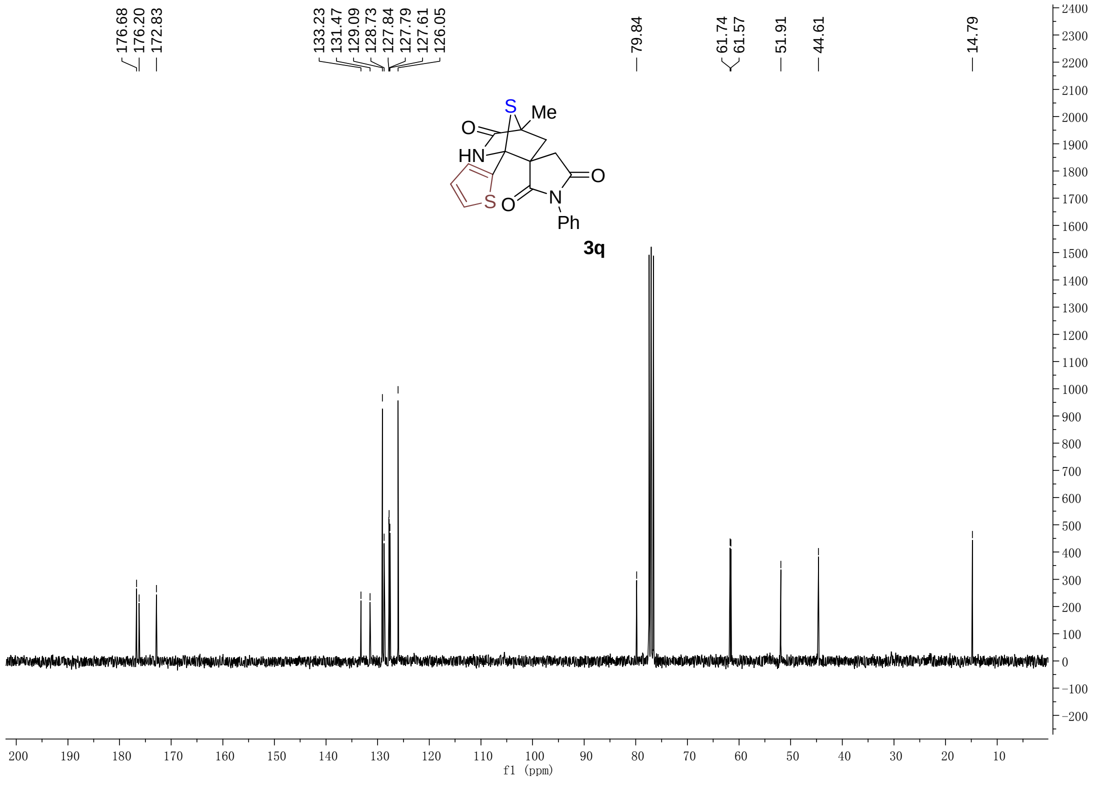
176.68
176.20
172.83
133.23
131.47
129.09
128.73
127.84
127.79
127.61
126.05
79.84
61.74
61.57
51.91
44.61
14.79
S
Me
O
HN
S
O
N
O
Ph
3q
f1 (ppm)

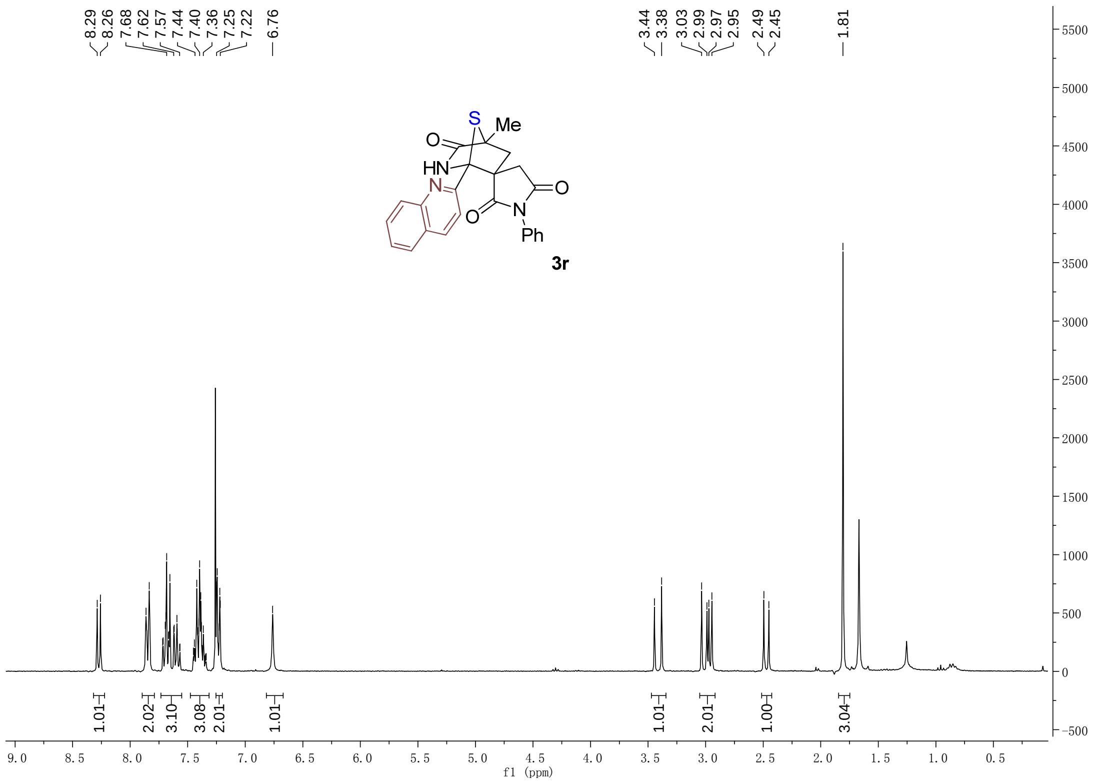

8.29
8.26
7.68
7.62
7.57
7.44
7.40
7.36
7.25
7.22
6.76
3.44
3.38
3.03
2.99
2.97
2.95
2.49
2.45
1.81
S
Me
O
HN
N
O
O
N
Ph
3r
1.01
2.02
3.10
3.08
2.01
1.01
1.01
2.01
1.00
3.04
f1 (ppm)

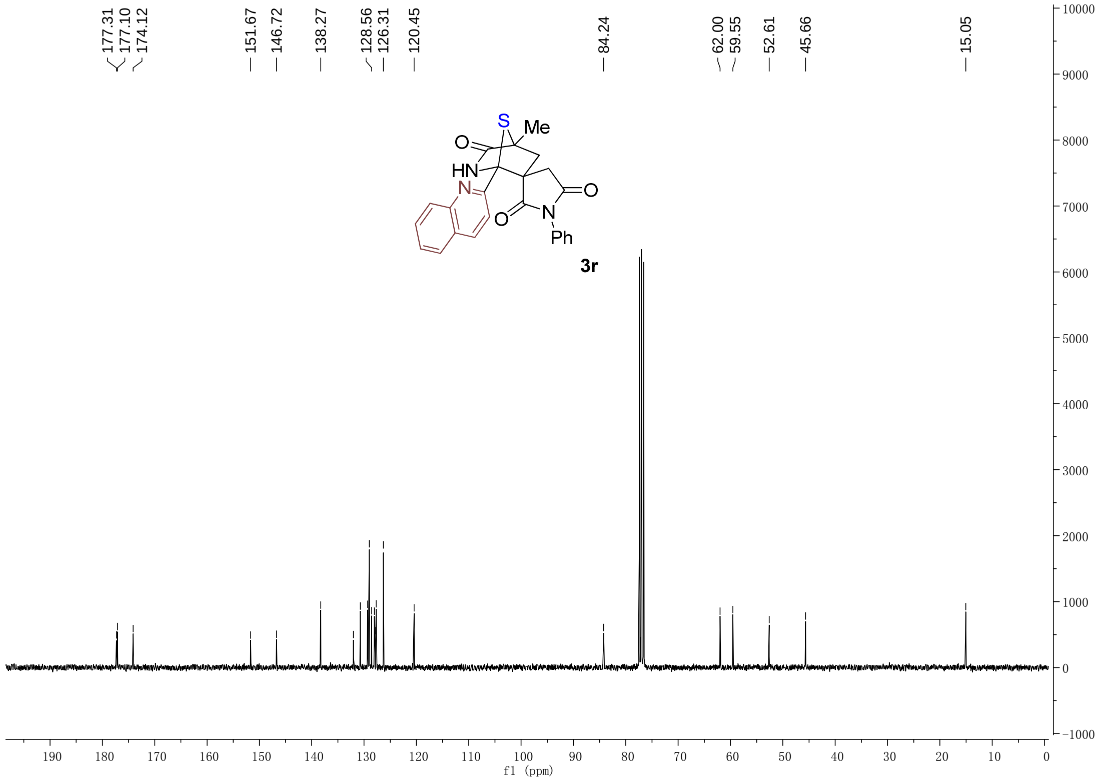

177.31
177.10
174.12
151.67
146.72
138.27
128.56
126.31
120.45
84.24
62.00
59.55
52.61
45.66
15.05
S
Me
O
HN
N
O
O
N
Ph
3r
f1 (ppm)

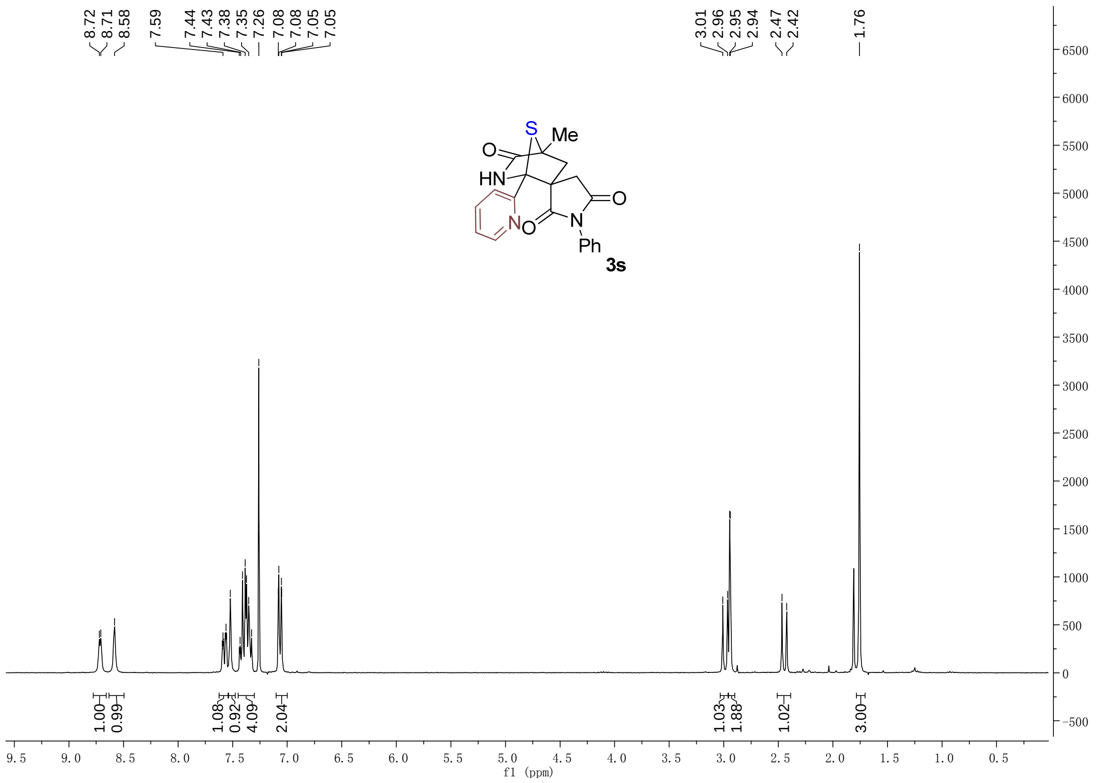
8.72
8.71
8.58
7.59
7.44
7.43
7.38
7.35
7.26
7.08
7.08
7.05
7.05
3.01
2.96
2.95
2.94
2.47
2.42
1.76
S
Me
O
HN
N
O
O
N
Ph
3s
1.00
0.99
1.08
0.92
4.09
2.04
1.03
1.88
1.02
3.00
f1 (ppm)

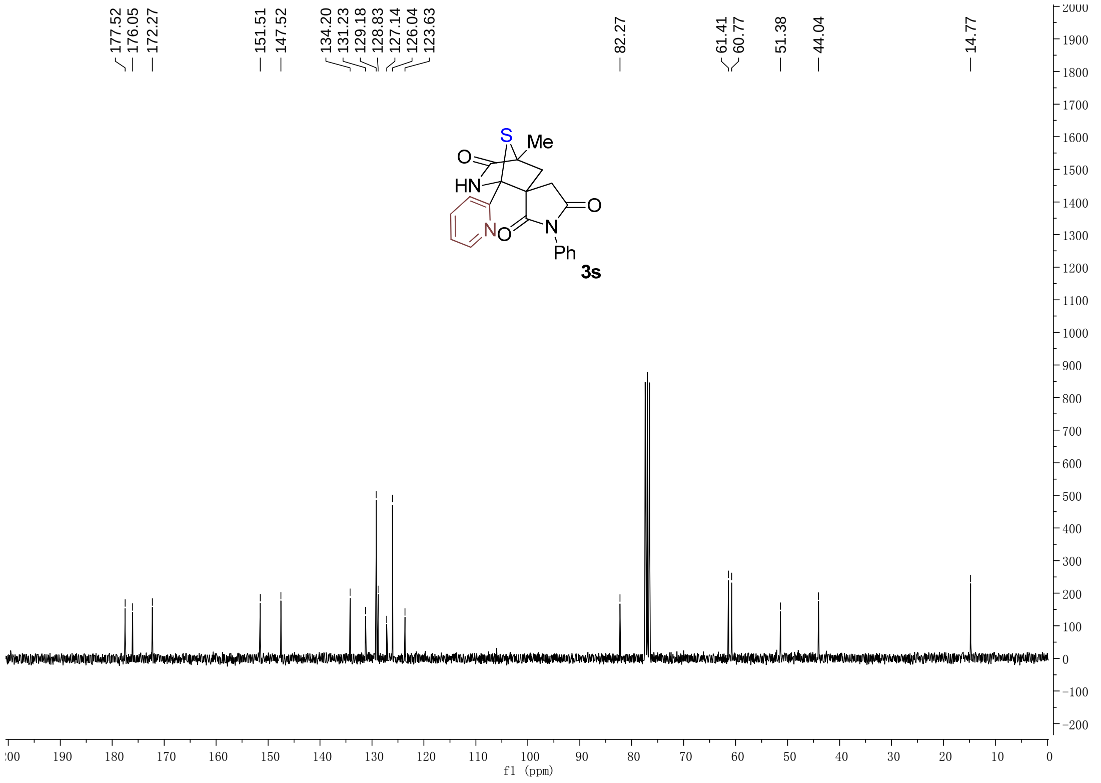
177.52
176.05
172.27
151.51
147.52
134.20
131.23
129.18
128.83
127.14
126.04
123.63
82.27
61.41
60.77
51.38
44.04
14.77
S
Me
O
HN
N
O
O
N
Ph
3s
f1 (ppm)

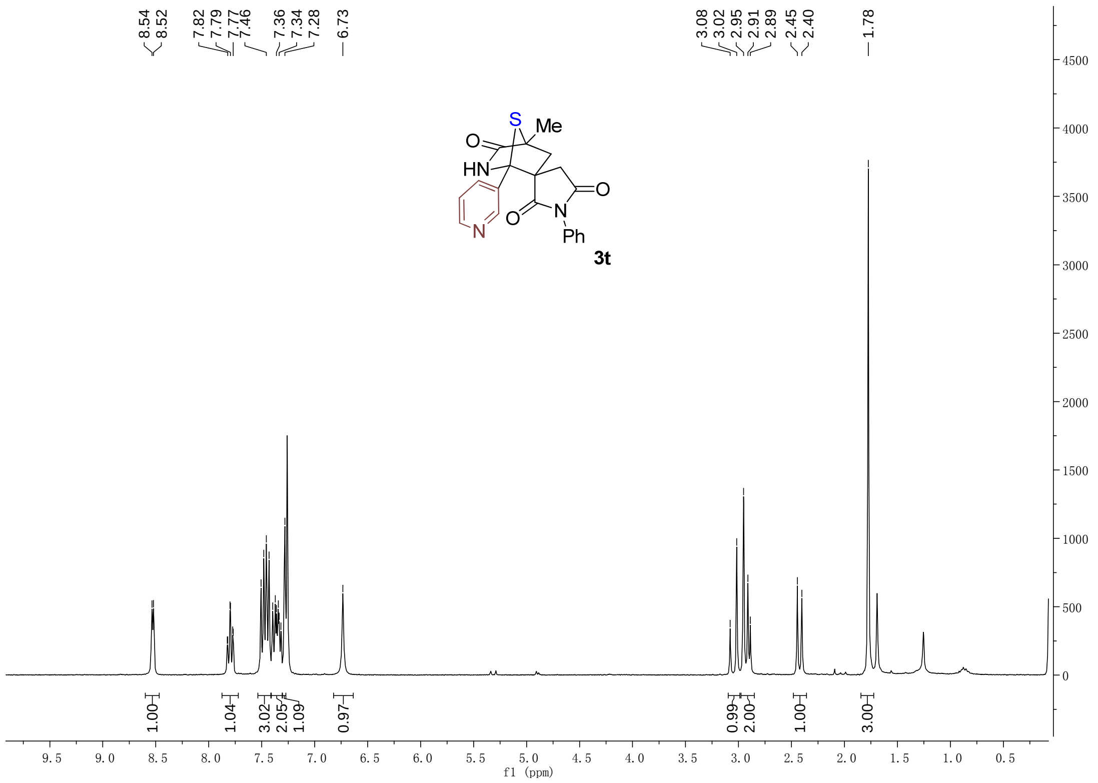
8.54
8.52
7.82
7.79
7.77
7.46
7.36
7.34
7.28
6.73
3.08
3.02
2.95
2.91
2.89
2.45
2.40
1.78
S
Me
O
HN
N
O
N
O
Ph
3t
1.00
1.04
3.02
2.05
1.09
0.97
0.99
2.00
1.00
3.00
f1 (ppm)

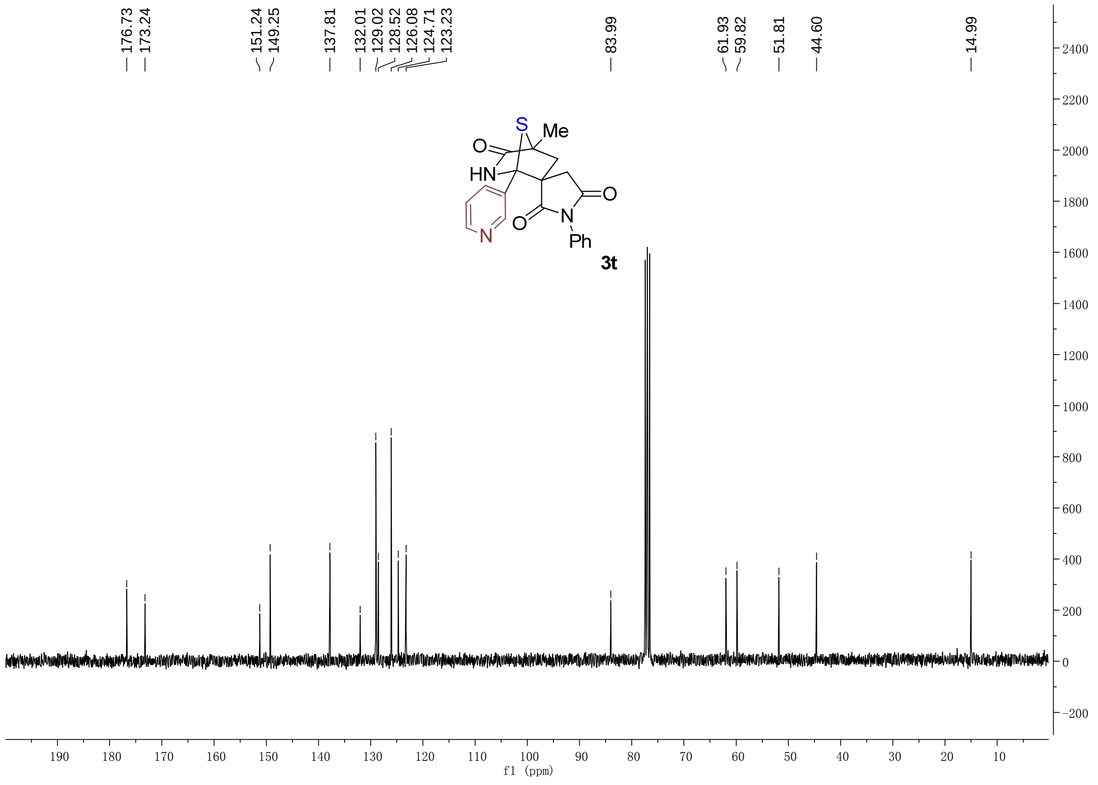
176.73
173.24
151.24
149.25
137.81
132.01
129.02
128.52
126.08
124.71
123.23
83.99
61.93
59.82
51.81
44.60
14.99
S
Me
O
HN
N
O
N
O
Ph
3t
f1 (ppm)

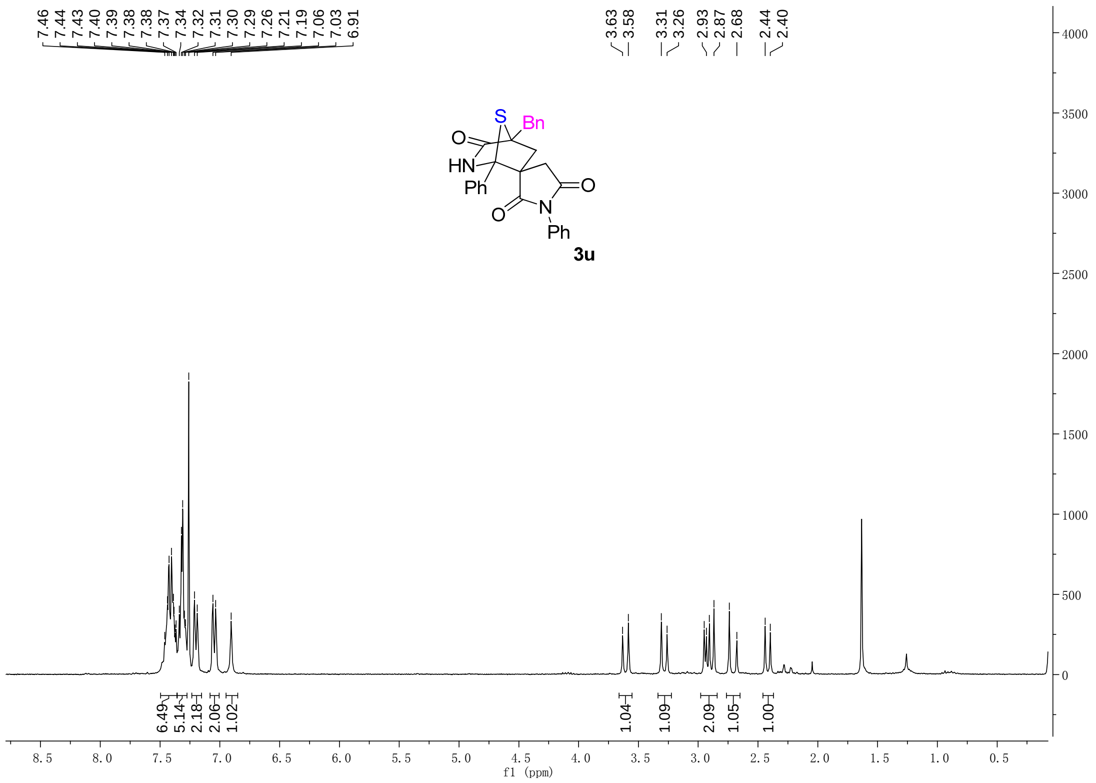
7.46 7.44 7.43 7.40 7.39 7.38 7.38 7.37 7.34 7.32 7.31 7.30 7.29 7.26 7.21 7.19 7.06 7.03 6.91
3.63 3.58 3.31 3.26 2.93 2.87 2.68 2.44 2.40
S
Bn
O
HN
Ph
O
N
O
Ph
3u
6.49 5.14 2.18 2.06 1.02
1.04 1.09 2.09 1.05 1.00
f1 (ppm)

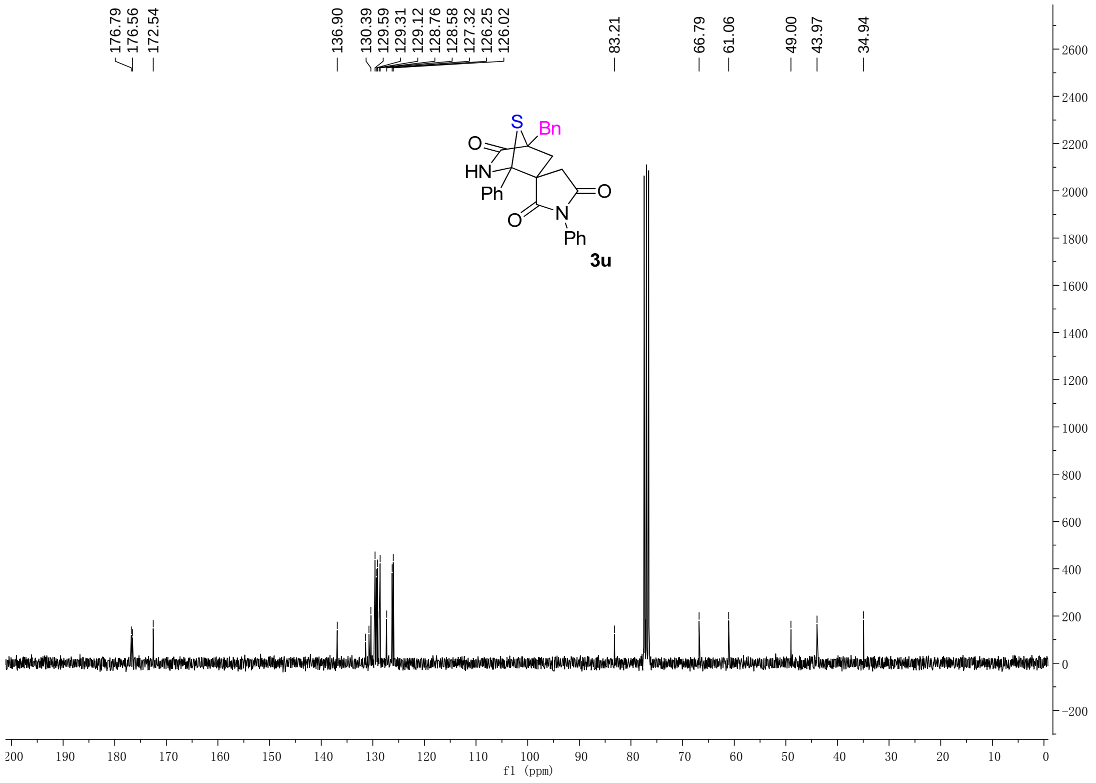
176.79 176.56 172.54
136.90 130.39 129.59 129.31 129.12 128.76 128.58 127.32 126.25 126.02
83.21 66.79 61.06 49.00 43.97 34.94
S
Bn
O
HN
Ph
O
N
O
Ph
3u
f1 (ppm)

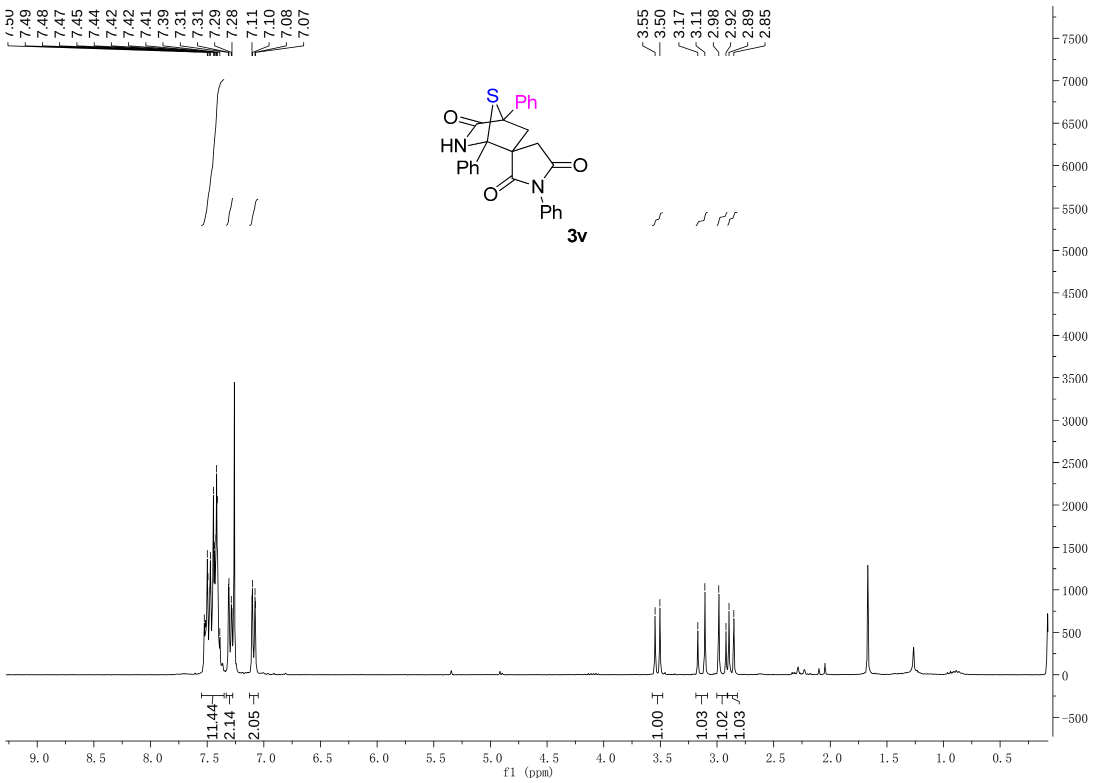
7.50
7.49
7.48
7.47
7.45
7.44
7.42
7.42
7.41
7.39
7.31
7.31
7.29
7.28
7.11
7.10
7.08
7.07
3.55
3.50
3.17
3.11
2.98
2.92
2.89
2.85
S
Ph
O
HN
Ph
O
N
O
Ph
3v
11.44
2.14
2.05
1.00
1.03
1.02
1.03
f1 (ppm)

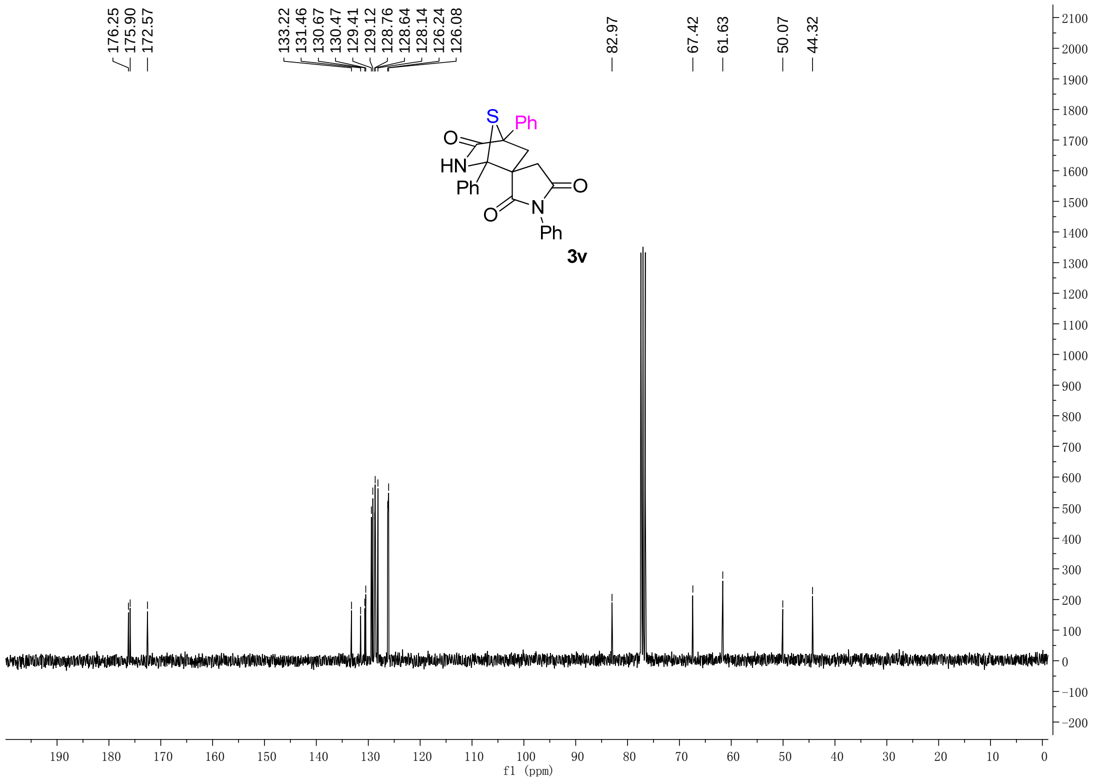
176.25
175.90
172.57
133.22
131.46
130.67
130.47
129.41
129.12
128.76
128.64
128.14
126.24
126.08
82.97
67.42
61.63
50.07
44.32
S
Ph
O
HN
Ph
O
N
O
Ph
3v
f1 (ppm)

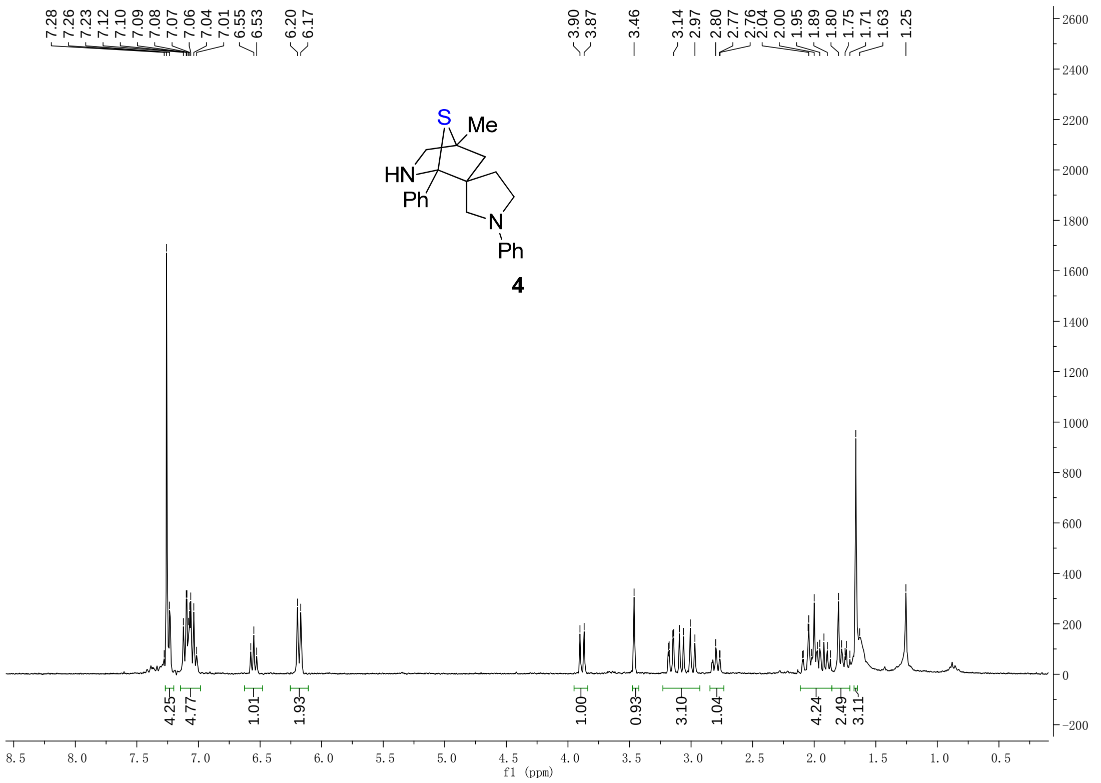
7.28
7.26
7.23
7.12
7.10
7.09
7.08
7.07
7.06
7.04
7.01
6.55
6.53
6.20
6.17
3.90
3.87
3.46
3.14
2.97
2.80
2.77
2.76
2.04
2.00
1.95
1.89
1.80
1.75
1.71
1.63
1.25
S
Me
HN
Ph
N
Ph
4
4.25
4.77
1.01
1.93
1.00
0.93
3.10
1.04
4.24
2.49
3.11
f1 (ppm)

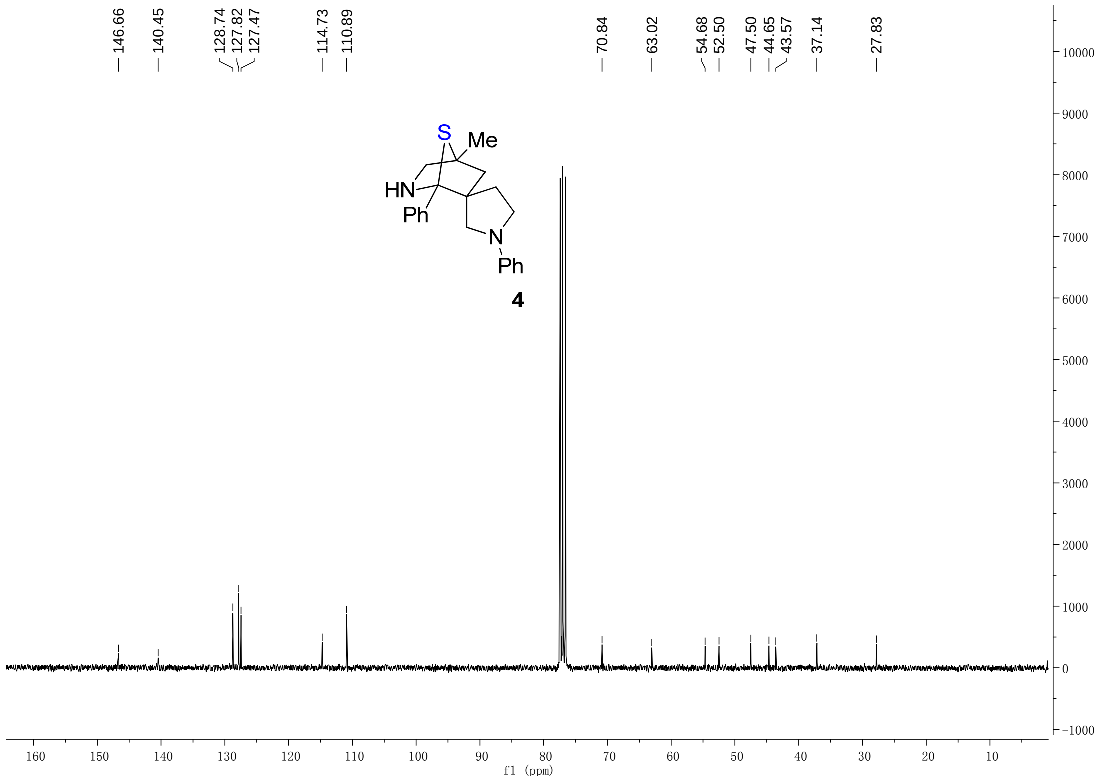
146.66
140.45
128.74
127.82
127.47
114.73
110.89
70.84
63.02
54.68
52.50
47.50
44.65
43.57
37.14
27.83
S
Me
HN
Ph
N
Ph
4
f1 (ppm)
